# Maintaining function and participation through tailored 24-hour movement behaviours for people living with multiple long-term conditions and frailty (The PERSONAL-AGILITY study): Protocol for a randomised controlled feasibility trial

**DOI:** 10.1371/journal.pone.0348372

**Published:** 2026-05-18

**Authors:** Martha Thomas, Louisa Y. Herring, Melanie J. Davies, Lucina Wilde, Vicky Hall, Laura J. Gray, Sharlene A. Greenwood, Michelle Hadjiconstantinou, Malak Hamza, Andrew Harris, Jemma Hawkins, Kamlesh Khunti, Matthew Maddocks, Atulkumar Rajpara, Alex Rowlands, Priscilla Sarkar, Avan A. Sayer, Sam Seidu, Tom Yates, Hannah M. L. Young

**Affiliations:** 1 NIHR Leicester Biomedical Research Centre, University of Leicester and University Hospitals of Leicester NHS Trust, Leicester, United Kingdom; 2 Diabetes Research Centre, College of Life Sciences, University of Leicester, Leicester, United Kingdom; 3 Leicester Diabetes Centre, University Hospitals of Leicester NHS Trust, Leicester, United Kingdom; 4 Active Together, Leicestershire County Council, Glenfield, Leicester, United Kingdom; 5 Division of Public Health and Epidemiology, University of Leicester, Leicester, United Kingdom; 6 NIHR Applied Research Collaboration East Midlands, University of Leicester, Leicester, United Kingdom; 7 Leicester British Heart Foundation Centre of Research Excellence, University of Leicester, Leicester, United Kingdom; 8 Department of Renal Medicine, King’s College Hospital NHS Trust, London, United Kingdom; 9 Renal Sciences, Faculty of Life Sciences and Medicine, King’s College London, London, United Kingdom; 10 Centre for Development, Evaluation, Complexity and Implementation in Public Health Improvement (DECIPHer), School of Social Sciences, Cardiff University, Cardiff, United Kingdom; 11 Wolfson Centre for Young People’s Mental Health, Cardiff University, Cardiff, United Kingdom; 12 Cicely Saunders Institute of Palliative Care, Policy & Rehabilitation, Kings College London, England; 13 AGE Research Group, NIHR Newcastle Biomedical Research Centre, Translational and Clinical Research Institute, Faculty of Medical Sciences, Newcastle University and Newcastle Upon Tyne Hospitals NHS Foundation Trust, Newcastle-Upon-Tyne, United Kingdom; PLOS: Public Library of Science, UNITED STATES OF AMERICA

## Abstract

**Introduction:**

Maintaining function and participation through tailored 24-hour movement behaviours for people living with multiple long-term conditions (MLTC) and frailty (The PERSONAL-AGILITY study) is a research programme which aims to develop and evaluate a complex behaviour change intervention. The intervention uses a range of approaches tailored for individuals living with MLTC and frailty, and their carers, incorporating novel technology to improve 24-hour movement behaviours and, consequently, quality of life and physical function. This paper outlines the protocol for a randomised controlled trial (RCT) evaluating the feasibility and acceptability of the PERSONAL-AGILITY intervention, study design and procedures.

**Methods and analysis:**

A feasibility RCT, with 2:1 allocation to the PERSONAL-AGILITY intervention or usual care control. The primary outcome is the feasibility and acceptability of a future definitive RCT investigating the effectiveness of the PERSONAL-AGILITY intervention, assessed against prespecified progression criteria, including eligibility, recruitment, retention, and outcome measure completion rates. The trial will be conducted at the University Hospitals of Leicester and comprise a 24-week tailored behaviour change intervention delivered by members of the PERSONAL-AGILITY research team. A target sample of 50 people with MLTC and frailty, and their carer (optional), will be recruited. A range of secondary outcomes including body composition, blood pressure, physical activity, sleep, physical function, and quality of life will be assessed at baseline, 12, and 24 weeks to help identify the primary or co-primary outcomes for participants with MLTC and frailty and their carers. A mixed methods process evaluation will examine the acceptability, feasibility and fidelity of intervention delivery and trial procedures. This study will inform the feasibility and design of a future definitive RCT.

**Ethics and dissemination:**

The trial has been approved by South Central Oxford B Research Ethics Committee (Ref: 24/SC/0367). Participants will be asked to provide consent to take part in the PERSONAL-AGILITY study by a trained member of the research team. Findings will be disseminated via peer reviewed journals, presentations and communications with health care professionals, patients and the public. Trial registration: International Standard Randomised Controlled Trial Number 14362764. URL to registry record: https://www.isrctn.com/ISRCTN14362764. Date of registration: 25^th^ November 2024.

## Introduction

An 86% increase in the number of people living with two or more long-term conditions (multiple long-term conditions, MLTC or multimorbidity) [1] is predicted by 2035 [2]. Compared with individuals living with a single condition, those with MLTC are more likely to experience a range of adverse health outcomes, including reduced quality of life, diminished physical function [2,3], and an increased risk of premature mortality.[4] Living with MLTC also increases the risk of frailty, a syndrome characterised by decreased physiological reserve leading to increased susceptibility to minor health stressors [5]. People living with both MLTC and frailty are especially vulnerable to poor outcomes such as loss of independence and reduced social participation [6–8].

Up to 90% of people living with MLTC and frailty fail to meet current physical activity guidelines [9], however, structured exercise interventions can benefit individuals living with MLTC and frailty [10], although, adherence is often low [11]. Increasingly, the importance of 24-hour movement behaviours, including physical activity, sedentary behaviour, and sleep, are being recognised for people both with [12] and without long-term conditions [13]. Individuals with MLTC and frailty typically accumulate high levels of sedentary behaviour [14], experience poor sleep [15,16], and engage in low levels of physical activity [9,14]. A 24-hour movement approach may therefore offer additional, more flexible opportunities to support health and wellbeing in this population [10,14,17]. Furthermore, the National Health Service (NHS) 10-year health plan aims to shift healthcare towards greater use of digital tools and services to support self-management and patient empowerment [17]. This approach aligns well with the diverse and variable needs of this population and with guidance from the National Institute for Health and Care Excellence, which emphasises shared decision making (SDM) as a key element of high-quality healthcare [18]. Despite this, research in this area remains limited [14], a finding consistent with the 2021 Cochrane Systematic Review, which concluded that there was a limited evidence-base for interventions targeting people living with MLTC overall [19].

The World Health Organisation also recommends a socioecological approach to increasing physical activity [20], which has also been advocated in the context of MLTC [21]. Despite this, most physical activity interventions continue to focus primarily at the individual level [22]. More sustained impact is likely when individual-level strategies are combined with interpersonal (e.g., carer) and community support.[20] Carers play a crucial role in supporting physical activity uptake and adherence among those they care for [23]. However, caregiving is associated with poor physical and mental health [24–27], and carers themselves are at increased risk of developing MLTC [28] and frailty [29,30]. Evidence suggests that care recipients and carers mutually influence each other’s behaviours, highlighting the need for interventions that engage both groups [31–34]. Furthermore, embedding activity into meaningful, enjoyable, and culturally appropriate activities and linking individuals with community-based opportunities may help them to sustain positive behaviours [23,35].

To date, there have been no published RCTs that have employed a socioecological, SDM-based intervention to address 24-hour movement behaviours among people living with MLTC and frailty [14,36].

### Aims and objectives

#### Aim.

The PERSONAL-AGILITY Study, funded by the National Institute for Health and Care Research (NIHR), aims to evaluate the feasibility and acceptability of a holistic and flexible 24-hour movement behaviour intervention, supported by SDM, digital tools, and linking with community-based physical activity options, for people living with MLTC and frailty and their carers.

### Objectives

- To determine the feasibility of an RCT investigating the effectiveness of the PERSONAL-AGILITY intervention for people living with MLTC and frailty and their carers.
- To assess the acceptability of the PERSONAL-AGILITY intervention for people living with MLTC, their carers, and healthcare professionals involved in its delivery, including exploration of key influences relevant to future implementation.
- To examine the potential effectiveness of the PERSONAL-AGILITY intervention for individuals living with MLTC, and their carers, compared to usual care, on a range of secondary outcomes at 12 and 24 weeks.
- To evaluate the implementation of the PERSONAL-AGILITY intervention, including fidelity, potential mechanisms of impact and the influence of contextual factors.

## Methods and analysis

This protocol is reported in accordance with the Standard Protocol Items Recommendations for Interventional Trials (SPIRIT 2025) guidance (S1 Table) [37,38] and template for intervention description and replication (TIDieR) checklist and guidance (S2 Table) [39]. The latest version of the protocol at the time of publication (version 4 (25th November 2025)) is included in S1 File.

### Study design

**Fig 1:**
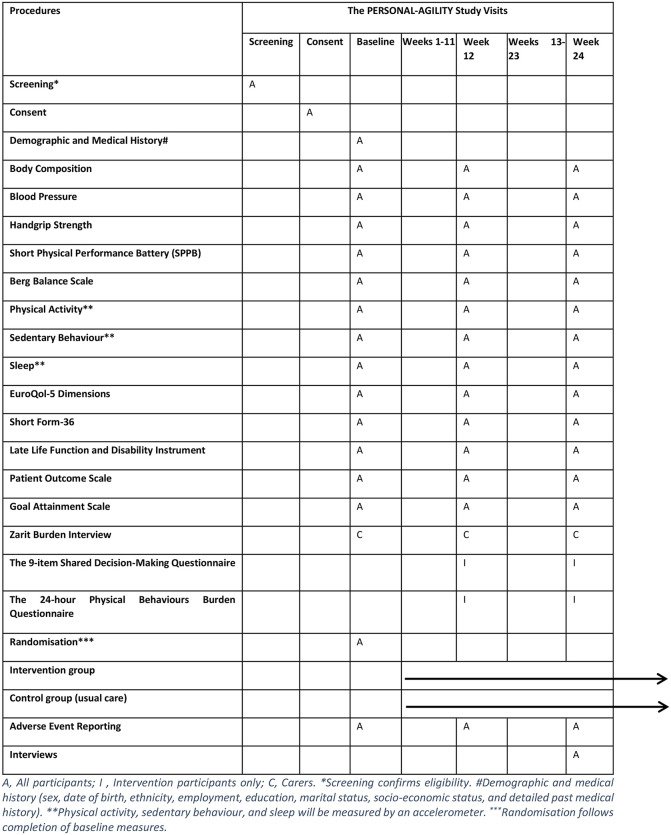
The PERSONAL-AGILITY SPIRIT schedule of procedures.

**Fig 2:**
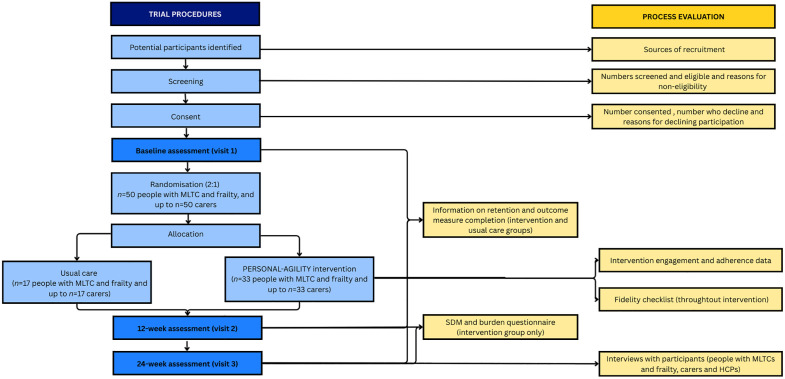
The PERSONAL-AGILITY study flow chart. Abbreviations: MLTC, Multiple long-term conditions; HCPs, Health care professional.

The trial is a 24-week feasibility RCT with a nested mixed methods process evaluation. A SPIRIT statement is provided in Fig 1. An overview of the trial design, core trial visits, and process evaluation are provided in Fig 2. The World Health Organisation Trial Registration Data Set is included in S3 Table.

### Trial setting

Participants with MLTC and frailty are being recruited through multiple pathways, including primary and secondary care settings (e.g., participant identification centre sites, outpatient clinics, and community health centres) within the United Kingdom, England. Additional recruitment routes include volunteer databases, engagement with community and charity organisations, and public advertisement, including social media.

To maximise recruitment efficiency, electronic health record searches will be conducted as a single, comprehensive search using predefined eligibility criteria. Within primary care, the clinical team will conduct database searches on electronic health records/ clinical systems (i.e., SystmOne) to identify potential participants. Where appropriate, GP practices may use electronic record prompts and identify eligible patients opportunistically during routine consultations to support recruitment.

Eligible individuals will be sent a study invitation letter, brief participant information leaflet, a QR code linking to the study website, and a reply slip. Within secondary care, the study team will attend relevant secondary care clinics to discuss the study with interested individuals following discussions with their healthcare providers.

### Eligibility criteria

All participants must meet the following criteria:

- Adults (≥18 years).
- Mobile (able to walk 5m with or without walking aids).
- Able and willing to provide informed consent.
- Able and willing to use digital/online tools with support as part of the intervention.

Additional criteria for participants living with MLTC and frailty:

- Living with MLTC (defined as any ≥ 2 long-term conditions for which exercise has demonstrated evidence of benefit or an unclear effect) [10]. Initially, participants were required to have a diagnosis of Type 2 Diabetes Mellitus (T2DM), confirmed by medical history or a Haemoglobin A1c (HbA1c) measurement of ≥6.0% (≥42 mmol/mol) within three months prior to enrolment. Following the randomisation of seven participants (four allocated to the intervention group), it became evident that this requirement inadvertently framed T2DM as the ‘index’ condition, shifting the trial focus toward comorbidities. To better align with the study’s aim of giving equal importance to all LTCs, the protocol was amended to remove T2DM as a requirement.
- Living with frailty, defined as a Clinical Frailty Scale (CFS) score of 4–7 (very mild frailty to severe frailty) or electronic frailty index (eFI) >0.12.

One carer per participant with MLTC and frailty, as identified by the person with MLTC, may participate alongside the participant.

Additional inclusion criteria for carers:

- Providing regular informal care to someone living with MLTC and frailty for ≥3 months. Informal caring is defined as including (but not limited to) emotional support, prompting with taking medications, getting prescriptions, managing, and organising appointments and care tasks, encouraging participation in social events and physical activity, helping with household tasks, or providing physical care.

Exclusion criteria (all participants):

- Unable to provide informed consent.
- Unable to communicate in English.
- Known contraindications to exercise (as defined by the American College of Sports Medicine) [40].
- Current participation in an ongoing clinical trial (as determined by the study investigator).
- Significant cognitive impairment (Mini-Mental State Examination score <24) [41] or unstable psychiatric disorder limiting participation.
- Serious illness or event with life expectancy <1year, active malignancy (on chemotherapy/radiotherapy), or other significant illness that, in the opinion of a study clinician, precludes involvement.
- Self-reportedly already engaging in ≥150 minutes of moderate-to-vigorous physical activity per week.

Additional exclusion criteria for carers:

- Providing paid or professional care

### Consent

Written informed consent will be obtained by members of the research team who are trained and authorised to undertake this duty.

Consent may be taken either in person or remotely (via telephone or secure video call) to minimise participant burden. Participants will be given sufficient time (at least 24-hours) to review the participant information sheet and ask any questions.

Participants are free to withdraw from the study at any time for any reason without prejudice to future care. Data collected prior to withdrawal will be retained and used in the study analysis.

### Randomisation

Randomisation and allocation will be performed by a member of the PERSONAL-AGILITY study team after baseline assessments using a validated password-protected wed-based system from a third-party service (Sealed Envelope Ltd). Randomisation will use variable block sizes. Participants will be stratified based on frailty status (very mild and mild/ moderate and severe).

Participants with MLTC and frailty will be randomly allocated in a 2:1 ratio, in favour of the intervention group, to either receive the PERSONAL-AGILITY intervention (intervention) or ongoing usual care (control). Unequal randomisation will provide more opportunity to collect data about acceptability and feasibility of delivering the intervention. Allocation will be stratified according to frailty status. Carers, where included, will be randomised as a dyad with the participant with MLTC and frailty. The trial aims to recruit 50 participants (excluding carers); 33 will be allocated to the intervention and 17 to the control group.

### Intervention

The Medical Research Council framework for complex interventions was selected as the overarching framework, guiding the decision to focus on feasibility testing to address uncertainties related to both trial and intervention design and to inform future refinement [42]. INDEX guidance for intervention development was used to guide the development of the PERSONAL-AGILITY intervention [43]. In line with this guidance, Intervention Mapping [44] and the Person-Based Approach [45] were selected as the development approaches.

Intervention Mapping was selected because it offers a comprehensive, systematic, and iterative approach to intervention development and has previously been used successfully to develop complex interventions in people with a range of long-term conditions [44,46–48]. The first four steps of Intervention Mapping were purposefully followed, as these focus specifically on intervention development.[44] At the end of the initial development phase, think-aloud interviews, advocated within the Person-Based Approach, were integrated to refine the intervention [45]. Think-aloud interviews have been used in a range of behaviour change interventions for people with MLTC and carers, [49,50] including those which focus on physical activity and the use of digital tools [51]. This approach enabled us to explore how a range of users engaged with the digital tools (see below) included in the intervention, helping to make them easier to use and more appealing and relevant, thereby enhancing acceptability at an early stage.[48,45].

INDEX guidance, Intervention Mapping and The Person-Based Approach all advocate the involvement of a diverse range of interest-holders (including those involved in intervention development or delivery, target users, and professionals with relevant experience) throughout [43,44,45]. In line with this, PERSONAL-AGILITY was co-produced with people with MLTC, carers, and health care professionals (HCPs). The unique contributions of interest-holders’ perspectives, experience and expertise were considered of equal value [52,53]. Throughout the development process, a range of strategies were used to ensure transparency, inclusivity, sharing of power, reciprocity, and relationship building. Further details of the development phase will be reported in a future publication.

**Fig 3:**
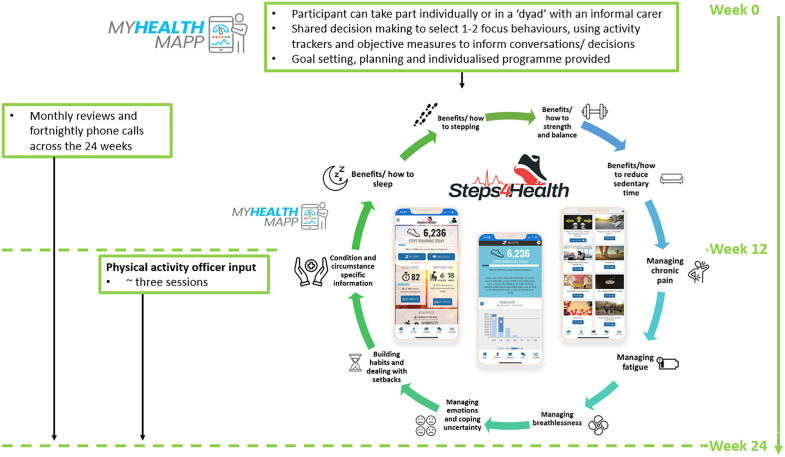
Outline of the PERSONAL-AGILITY Intervention.

PERSONAL-AGILITY is a theoretically-informed complex intervention, summarised in S2 Table and outlined in Fig 3. The aim of the intervention is to support participants to improve one or more of the 24-hour movement behaviours (sleep, physical activity, and sedentary behaviour), subsequently impacting physical function and quality of life. The intervention uses a range of approaches tailored to individual needs and is supported by technology (MyHealthMapp and Steps4Health) to enhance, rather than replace, face to face interactions.

The 24-week intervention will be delivered by physiotherapists and exercise specialists (who are members of the research team) and physical activity officers based in the local authority through a range of delivery methods (in person, by phone, or online) as one-on-one sessions. Intervention providers will not be blinded to allocation due to the nature of the intervention. Participants in a carer dyad will have the option to attend sessions together or separately, according to the participant’s preference. Intervention appointments will be conducted in person or remotely (via telephone or video call), according to participant preference. The intervention will commence with a holistic assessment, using a SDM approach to select movement behaviour(s) most appropriate to the individual and what matters to them [54]. SDM is a collaborative process in which the individual and their HCP jointly make decisions about care that best meets the individual’s needs [54]. Each decision and intervention goal will take into account participant’s preferences and values, HCP knowledge, and the evidence around treatment options [54].

SDM will be facilitated using a patient decision aid provided to participants before their first intervention appointment. The aid is designed to support engagement and SDM by eliciting what matters most to them; weighing up the personal benefits and risks of 24-hour movement behaviours; addressing common worries; identifying who may support them throughout the intervention; and explaining what SDM is and how it will be used during their appointments. Participants in a carer dyad will also receive a guide to help them work together effectively, including understanding each other’s goals, priorities, and challenges. Information gathered will be used to determine participant goals and to formulate an individualised intervention plan.

To support participants throughout the programme, wearable technology (e.g., Fitbits) will be provided. Participant confidence and familiarity with digital tools will be determined at baseline using the 3-Item Digital Health Care Literacy Scale [55]. Intervention participants scoring six or below will receive additional support to use digital tools as needed. Where necessary, participants will also be provided with an iPad and/or internet access. Data from wearable technology, manual entry and from patient-reported outcome measures (separate to the trial outcome measures) will be captured by MyHealthMapp. MyHealthMapp is a web-based tool originally developed for people with type 2 diabetes, designed to support SDM conversations between people living with LTCs and their healthcare professionals about 24-hour movement behaviours.[56,57].

MyHealthMapp compares personal data with population recommendations and presents them using a red-amber-green (RAG) rating to aid interpretation. It also provides brief educational videos and generates personalised targets (e.g., step goals) based on prior results, supporting the tracking of health, wellbeing and 24-hour movement behaviour patterns over time. Adaptations for people with MLTC and frailty and carers include the collection of additional measures relevant to this population (number of falls, a measure of balance, confidence in balance, symptom burden and distress) and optimisation of the tool to support user engagement and understanding.

MyHealthMapp can generate personalised videos to summarise individual data of interest to the participant, explaining what the results mean in clear, accessible language. The videos can be paused or re-watched, shared with family or carers, and used to help participants reflect on priorities and prepare for their appointment [58–61]. Their aim is to enable participants to take a more active role in their care, support informed discussions, allowing more time for questions, and supporting SDM and goal setting [58–61].

Participants will also be given access to Steps4Health, [62] a web-based physical activity tool designed to support 24-hour movement behaviours in people who want to become more active or who are living with LTCs. The platform includes personalised targets (e.g., stepping, sitting, sleep, sweating and strengthening), on-demand exercise sessions for all abilities, daily activity tracking, expert support, interactive educational content, challenges and rewards, and a peer support chat forum. Participants can use Steps4Health *ad libitum* and may be prompted by the research team to explore specific parts of the programme when relevant. Adaptations made for people with MLTC and frailty and carers include the inclusion of additional educational and exercise sessions specifically targeted to the needs of this population.

As part of the intervention participants are followed up monthly, interspersed with two-weekly telephone check-ins. Monthly sessions will be individualised and are expected to last approximately 1 hour. Where feasible, personalised videos will be provided in advance of appointments to help participants reflect on priorities and prepare for their appointment [58–61]. During these appointments, data from MyHealthMapp will be used to review and, where required, revise plans and goals, using a SDM approach and embedded behaviour change techniques (BCTs). Interim calls are anticipated to last between 15 and 30 minutes and will be used to troubleshoot challenges and facilitate motivation.

At 12 weeks, participants may be linked with local community groups that either directly (e.g., an exercise class) or indirectly (e.g., a group without a physical activity focus, but which promotes movement through travel or other activities) to increase physical activity and/or reduce sedentary behaviour. This will be facilitated by physical activity officers and community providers, who will identify suitable local community groups which align with the participants goals and interests and connect and support participants to attend.

All members of the intervention delivery team will be trained in SDM and BCTs associated with the intervention and will observe peer sessions to ensure consistent intervention delivery.

### Usual care

Participants in both groups (intervention and control) will receive ongoing standard care, as delivered by the participants’ usual National Health Service (NHS) providers in primary and secondary care. Usual care will reflect routine practice in which participants may be referred into single LTC rehabilitation. Participants in the control group will receive generic written information on the 24-hour movement behaviours and offered access to Steps4Health at the end of the trial.

### Outcome measures and progression criteria

Outcome data will be collected at baseline (prior to randomisation), 12 weeks, and 24 weeks post randomisation. Participants will be re-imbursed for travel costs associated with data collection visits to maximise retention. All data will be collected by members of the PERSONAL-AGILITY research team who will not be blinded to group allocation, as this feasibility study does not aim to evaluate intervention effectiveness.

Baseline assessment will include participant demographics (date of birth, age, sex, gender, ethnicity, frailty status, past medical history, medications) and information on socioeconomic status (living arrangements, housing status, employment status, receipt of benefits, level of education attainment, marital status, participant postcode to calculate participants deprivation decile). Where relevant, additional information will be collected from carers (relationship to the care recipient, length of time in current caring role, number of hours of care provided per week, caring tasks performed)

### Primary feasibility outcomes

To determine the acceptability and feasibility of a future RCT investigating the PERSONAL-AGILITY intervention. The following outcomes will be measured at 24-weeks using data from a range of resources, including screening and recruitment logs and case report forms (CRFs):

### Eligibility rate

Eligibility rates and reasons for ineligibility. Eligibility rate will be calculated as the number of participants meeting inclusion criteria divided by the number of participants approached, presented as a percentage. Sources of recruitment will be documented to explore the most successful avenues for recruitment to a future definitive trial.

### Recruitment rate

Recruitment rates will be calculated as: (1) the number of people consented divided by the number of people eligible, presented as a percentage, to provide an overall recruitment rate; (2) the number of carers consented divided by the number of people with MLTC and frailty who consent, presented as a percentage, to provide a carer recruitment rate; (3) the number of people with MLTC and frailty from ethnic minority backgrounds consented divided by the number of people with MLTC and frailty who consent, presented as a percentage, to provide a recruitment rate for people from ethnic minority backgrounds; and (4) the number of people with MLTC and frailty from socioeconomically deprived backgrounds consented divided by the number of people with MLTC and frailty who consent, presented as a percentage, to provide a recruitment rate for people from socioeconomically deprived backgrounds. The basic characteristics (age, sex, ethnicity, and frailty status if known) of those who decline to participate and reasons for declining will also be reported.

### Retention rate

Retention is defined as providing at least one complete secondary outcome measure at 24 weeks. Retention rate will be calculated as the total number of participants providing at least one outcome measure at 24 weeks divided by the total number of participants randomised, presented as a percentage.

### Outcome measure completion rates

Completion rates for all secondary outcome measures and reasons for missing data (if known). Completion rates for all secondary outcome measures will be calculated as the number of valid completions divided by the number of people available for follow up at each time point, presented as a percentage.

### Other feasibility measures

The feasibility of co-primary outcomes for carers will be explored based on recruitment and completion rates of carers and their feedback gathered from interviews (see process evaluation).

### Secondary outcomes

A number of secondary outcomes will be collected at baseline, 12 and 24 weeks to help identify the primary or co-primary outcome for both participants with MLTC and frailty and their carers. This decision will be informed by feasibility data on outcome measure completion rates at the end of this feasibility study, as well as input from the Patient and Public Involvement and Engagement (PPIE) group. A trained member of the research team will follow standard operating procedures for objective tests to ensure consistency, and all participants will complete the tests, unless exclusions apply.

To test the potential effectiveness of the PERSONAL-AGILITY intervention for people with MLTC (and their carers) compared to usual care, the following outcomes will be collected:

### Body composition

Body weight and height will be measured to the nearest 0.1 kg/cm. Body mass index (BMI) (kg.m^2^) will be calculated using body weight and height. Body composition, including fat mass, lean mass, appendicular lean mass and body fat percentage will be estimated using Bioelectrical Impedance Analysis.

### Blood pressure and heart rate

An automated sphygmomanometer will be used to measure arterial blood pressure (mmHg) and heart rate (bpm) after participants have been seated for at least 5 minutes. Three measurements will be taken and the average of the last two measurements will be recorded.

### Handgrip strength

A handheld dynamometer will be used to measure handgrip strength (kg), a method shown to have high test–retest reliability and strong validity for assessing maximal isometric grip force [63]. Three measurements will be taken from each side, making sure the forearm is in a neutral position and the elbow is flexed at a right angle [64]. The highest reading will be recorded as maximum grip strength (kg).

### Short physical performance battery

Physical function will be measured using the Short Physical Performance Battery (SPPB). The SPPB includes three tests: a hierarchical test of standing balance, usual gait speed and the ability to stand from a chair five times [65]. Each component of the SPPB is scored using arbitrary units individually, and the scores are then summed to provide a total ranging from 0 (worst performance) to 12 (best performance). The SPPB demonstrates good test-retest reliability in older people [65,66] and is responsive to change following an intervention in lower functioning older patients.[66,67].

### Berg balance scale

The Berg Balance Scale will be used to assess balance [68]. The test shows high levels of validity and reliability in a range of LTCs and in older populations [69,70]. The scale comprises 14 tasks which evaluates both dynamic and static balance. Each task is scored using a 5-point ordinal scale yielding a maximum total score of 56 [70].

### Accelerometer and inclinometer measured movement behaviours (physical activity, sedentary behaviour and sleep)

Participants will wear two devices for 9 consecutive days (24 hours/day) to capture 3–4 days with >16 hours of valid data [71]. A wrist-worn GENEActiv accelerometer on the non-dominant wrist will record triaxial acceleration [72] using GGIR software [73]. A thigh-worn ActivPAL inclinometer, attached midline on the anterior thigh, will quantify sitting, standing, and walking using ProcessingPAL software [74]. The ActivPAL is a validated tool for posture during daily activities [75]. Participants will complete a daily log of sleep times and monitor any removal to aid data analysis.

### EuroQol dimensions (EQ-5D-5L)

The EQ-5D-5L is a validated, preference (utility) based generic health related quality of life (HR-QoL) questionnaire [75,76]. The EQ-5D-5L assesses self-reported health across five domains and generates a single health status index ranging from values considered worse than death (negative values) to perfect health (1) [77].

### Short form-36 (SF-36)

The SF-36 is a valid and reliable 36-item measure of HR-QoL across multiple domains [78]. A computer-based scoring algorithm will be used to calculate scores. Scores ranged from 0–100, with higher scores reflecting better HR-QoL.

### Late-life function and disability instrument (Late-life FDI)

The Late-life FDI is a valid and reliable measure of function and disability, which has been shown to be responsive to change [79]. The measure consists of two sections: 1) disability, which assesses socially defined tasks and evaluates activities of daily living, and 2) function, which measures the ability to perform discrete actions and activities [80]. Higher scores indicate greater frequency of participation in life tasks (disability component) or higher functional ability (function component) [80].

### Patient outcome scale (POS)

The POS will be used to assess physical symptoms, psychological, emotional, and spiritual information, and support needs [81]. It is a widely used, acceptable and valid multidimensional 11 item measure of quality of life for people with LTCs [81]. Items are scored on a 5-point Likert scale, except question 9, which uses a 3-point scale and includes open-ended questions on main problems experienced by participants [81]. The total score is out of 40, with 0 indicating better quality of life and 40 representing poorer quality of life [81].

### Goal attainment scale (GAS)

The GAS is widely used in healthcare, including rehabilitation, physical therapy, and care of older populations and is well suited to an MLTC population where intervention goals and components might differ between individuals [82]. The GAS is a person-centred approach to measuring progress that involves setting individualised goals, defining expected outcomes, and rating achievement on a 5-point scale from −2 to +2 relative to those expectations [82].

### Zarit burden interview

The Zarit Burden Interview will be used for carers only. It is a validated and reliable 22-item questionnaire used to assess carers perceived burden of caregiving using a 5-point Likert scale [83]. The total score is out of 88, with higher scores indicating greater burden [83].

### Safety reporting

All adverse events (AEs) and serious adverse events (SAEs) will be recorded from the time a participant enters the study to the final study visit. Each AE will be considered for severity, causality and expectedness and may be reclassified as an SAE depending on the circumstances.

Due to the nature of the population included, participants are likely to experience adverse events throughout the course of the trial. People with MLTC and frailty have a large burden of disease and are likely to experience acute illness resulting in hospitalisations, new medical problems and deterioration of existing medical problems. Some AEs, such as outpatient appointments, or treatments for ongoing conditions present at the start of the study, are expected to occur due to the study population and will therefore, not be reported as outlined below.

An SAE is any untoward medical occurrence that:

- is life threatening
- requires prolonged existing hospitalisation or new inpatient hospitalisation
- results in persistent or significant incapacity or disability
- consists of a congenital anomaly or birth defect or other significant medical events
- results in death

All SAEs (from randomisation) will be reported to sponsor within 24-hours of becoming aware of the event including information on the description of the event, severity, date of onset and end date, assessment of relatedness to the study, and action taken/followed until resolution or when a final outcome has been reached.

An AE is a clinical investigation or any untoward medical occurrence in a participant which does not necessarily have a causal relationship with the trial. An AE is any unfavourable sign, symptom, or disease temporally associated with the study, whether or not considered related to it. Adverse Events that are considered to be directly related to the PERSONAL-AGILITY Study that are observed by the research team or reported by the participant (from randomisation) will be recorded including information on the description of the event, severity, date of onset and end date, assessment of relatedness to study and action taken/followed until resolution or when the event is considered stable.

### Sample size

Power calculations are not appropriate to determine sample size for a feasibility trial [84,85]. Sample sizes of 24–50 participants are generally considered adequate for addressing key uncertainties related to feasibility [86–90]. A total of 50 participants living with MLTC and frailty will be randomised.

### Process evaluation

#### Design.

An embedded mixed-methods process evaluation will be conducted according to the NIHR/ MRC guidelines [91,92]. Normalisation Process Theory (NPT) will serve as a sensitising framework to guide qualitative data collection and analysis of interviews [93,94].

#### Aim.

The aim of the process evaluation is to support the refinement of the intervention and trial design in readiness for a future definitive RCT by examining: (1) intervention implementation (fidelity to intervention delivery, adherence by the participants and reach), (2) intervention acceptability, (3) potential mechanisms of impact, (4) trial acceptability (procedures, outcomes, unintended harms and consequences) and (5) the influence of contextual factors across all of these domains.

#### Methods.

Table 1 outlines data collection methods and sources relevant to the process evaluation.

**Table 1:** Methods used and data collected within the PERSONAL-AGILITY trial process evaluation.

COMPONENT	IMPLEMENTATION			MECHANISM OF IMPACT	CONTEXT		INTERVENTION ACCEPTABILITY		TRIAL ACCEPTABILITY
PARTICIPANTS		HCPs*	Participants	Participants♯	HCPs*	Participants♯	HCPs*	Participants♯	Participants♯
**METHODS AND DATA COLLECTED**	Fidelity	Audio recording of sessions and Fidelity checklist SDM-Q-9 Questionnaire data from the intervention group	Engagement/ adherence process data^+^	Interview	Interview Site contextual information	Interview	Interview	Interview 24-hour Physical Behaviours Burden Questionnaire	Interview
	adherence	Process data on number of: reviews physical activity officer sessions community groups attended	Engagement/ adherence process data	Secondary outcome data					
	Reach	Process data on: characteristics of participants receiving the intervention characteristics of those retained in the trial and those who drop out							

Abbreviations: HCP, health and care professionals; SDM-Q-9, 9-Item Shared Decision-Making Questionnaire. *’HCP’ refers to those providers who are involved in the intervention delivery outside of the research delivery team, including physical activity officers and community providers. ♯ ‘Participants’ refers to people living with MLTC and frailty and carers with the intervention group and includes those who have withdrawn voluntarily

### Implementation

Assessments of implementation will include evaluations of fidelity, adherence, and reach. Fidelity will be assessed to determine the extent to which the core functions of the intervention were delivered as intended. Adherence will be examined through quantification of participant engagement with the different components of the PERSONAL-AGILITY intervention. Reach will be evaluated to ascertain the extent to which the intervention was delivered to the intended target population.

#### Implementation fidelity assessment.

All intervention sessions will be audio-recorded, with written consent from the participants. Audio-recordings will be used to code whether core intervention functions (HCP behaviours and core BCTs) were delivered, using a fidelity checklist and coding manual developed *a priori*. The development of the fidelity checklists was informed by guidance from Walton et al. (2020) [95].

Intervention materials were coded to identify key components, including the core functions, HCP behaviours, and BCTs identified during the intervention development phase from the BCT Taxonomy v1 [96]. Components were grouped and an intervention framework was developed, which was then reviewed to identify which functions and BCTs should be delivered in each intervention session by relevant intervention providers. For each intervention session type, intervention components to be delivered were listed sequentially, forming the basis for the development of five fidelity checklists. These checklists include both standardised components that all participants should receive and optional or tailored elements specific to participants (carer, MLTC and frailty or dyads). These checklists were reviewed by members of the study team to remove jargon and redundant items.

A coding manual was developed to provide guidance on each checklist item and how to appraise it, with examples, and training will be provided to ensure consistent use [97]. Components will be rated as: “definitely occurred”, “possibly occurred”, “did not occur”, “not applicable”, “missing”, or “unclear” [95]. An additional column will enable users to provide a rationale for scoring if the component was not rated as “definitely occurred” [95].

Two members of the PERSONAL-AGILITY research team will first pilot the checklist and coding manual on three audio recordings. Following refinement, further fidelity assessments will be conducted, with inter-rater reliability evaluated to establish agreement. Inter-rater agreement will be calculated using weighted kappa and percentage agreement [98,99]. If raters do not achieve weighted kappa scores ≥0.61 (good agreement), [95] for three consecutive audio recordings of the same type of appointment, additional recordings will be coded and discussed until agreement is reached. To minimise assessment bias, team members will not code their own appointments.

Approximately 18 audio-recordings will be randomly selected from all intervention sessions, including interim phone calls and physical activity officer sessions (where applicable). This represents approximately 10% of the intervention sessions delivered, which is consistent with recommendations [95]. A mixture of sessions from across the duration of the study will be selected to account for intervention provider practice effects.

In addition to these fidelity checklists intervention participants will complete the 9-item SDM questionnaire (SDM-Q-9) at weeks 12, and 24 to assess the HCP and participant behaviours during decision making and establish if SDM took place [100]. The SDM-Q-9 uses a 6-point Likert and has good acceptability and reliability [100].

#### Adherence.

Given that PERSONAL-AGILITY is a complex intervention comprising multiple components, a range of measures will be used to indicate participants’ uptake, engagement, and adherence to each component of the intervention:

- MyHealthMapp and Steps4Health

Data relating to uptake and engagement, including MyHealthMapp and Steps4Health uptake will be measured as the proportion of participants randomised to the intervention who access each of the tools at least once. Engagement will be measured by the number of times users logged-in to the tools over 24-weeks, and mean session log-in duration.

- Rates of adherence to the intervention

Adherence is defined as the amount of intervention received by people with MLTCs and carers [101]. Given that the intervention may focus on a range of potential movement behaviours which will be selected during a SDM process, and linking to and attendance at community services adherence will focus on a) the number and percentage of intervention reviews received (which also form one of the trial progression criteria, see below), b) adherence to the agreed plan as part of these reviews and c) uptake and adherence to community sessions.

Data on the number of intervention and percentage of reviews received will be gathered from intervention records. Data on adherence to the agreed plan as part of these reviews will be gathered from participants using a bespoke diary [91]. The diary will focus on the frequency with which the agreed plan developed as part of the intervention is carried out. This reflects the fact that plans are primarily carried out at home and to reduce participant burden. Uptake of community sessions will be measured as the proportion of participants randomised to the intervention who access at least one community session. Adherence will be measured by the number of community sessions attended across weeks 12–24 of the intervention. Data relating to community sessions will be collected from intervention records and a bespoke data capture form completed by the physical activity officer at 24 weeks.

#### Reach.

Information on the reach of the intervention will be obtained from participant characteristics drawn from baseline trial data as described earlier.

### Potential mechanisms of impact

Potential mechanisms of impact will be descriptive in nature and explored by analysing changes in secondary outcomes and through participant interviews.

### Contextual influences

In addition to the qualitative interviews described below, local authority profile questionnaires, informed by the Context and Implementation of Complex Interventions (CICI) framework, [102] will be collected prior to commencement of the study. These questionnaires will provide contextual information for each area and help situate the analysis and interpretation of findings, contributing to a better understanding of the context in which the intervention is delivered.

### Acceptability of trial methodology and intervention

The acceptability of the trial and intervention will be measured both quantitatively and qualitatively. Quantitative adherence data (described previously) will help to determine the acceptability of the PERSONAL-AGILITY intervention, based on the assumption that greater levels of adherence suggest higher levels of acceptability. In addition, the 24-hour Physical Behaviour Burden Questionnaire (adapted from the exercise therapy burden questionnaire [103]) will be used to assess intervention burden for participants.

### Qualitative data collection

Qualitative data on intervention and trial acceptability and potential mechanisms of impact will be gathered via semi-structured interviews. These data will be supplemented with other data from the trial as outlined previously.

Participants and carers enrolled in the trial, who are randomised to the intervention group, will be eligible to take part in the qualitative data collection for the process evaluation, including participants who voluntarily withdraw, and will be recruited as described for the feasibility trial. Healthcare professionals, including physical activity officers and other community providers, involved in the delivery of the PERSONAL-AGILITY intervention will also be eligible provided they are aged ≥18 years and able to provide written informed consent. These participants will be identified by the study team.

Qualitative process data will include semi-structured interviews with approximately 15 intervention participants, ~ *n* = 15 carers and ~*n* = 15 community providers involved in the intervention (subsequently collectively termed HCPs). Information power will be assessed throughout, and the final sample size determined by its ability to provide rich data which addresses the stated aims [104].

Maximum variation sampling of participants will be guided primarily by key characteristics relevant to each participant group. For individuals living with MLTC and frailty, sampling will be based on level of frailty. For carers, sampling will consider caring circumstances and the nature of caring relationships. For HCPs, sampling will be informed by their role and level of experience. These characteristics will be derived from demographic data collected via the questionnaire completed by participants following the consent process.

One-to-one semi-structured interviews will be used to gather qualitative data as they offer an open and flexible method for exploring the participants’ individual experiences in-depth. Interviews will be conducted in-person, or remotely via telephone or secure online software by a trained researcher at an appointment separate from the other trial assessments. Researchers will not interview participants to whom they delivered the intervention, in order to minimise the risk of influencing participants’ responses.

For MLTC and carer participants interviews will occur after the 24-week visit or when participants withdraw from the study, if they remain willing to participate in an interview. People with MLTC and frailty and carer interviews will be conducted separately. For HCPs interviews will occur at the end of the study for the physical activity officers and after the 24-week assessment for community providers.

Separate topic guides for each participant group have been developed in partnership with the PPIE group, guided by the PERSONAL-AGILITY programme theory and NPT. NPT is a middle-range implementation theory that has been used extensively to explore the processes underpinning implementation, embedding and integration of service innovation, and is well suited to multi-level intervention evaluation [93,94]. Therefore, topics guides are structured around the core constructs within NPT (coherence; cognitive participation; collective action; and reflexive monitoring). The first three interviews will act as pilots (but will be included within the overall analyses) and following these the guide may be adjusted. Guides will also be subject to adjustment as the trial progresses and the intervention is refined.

### Progression criteria

*A priori* progression criteria were developed by the study team and PPIE group to provide criteria on which to base the decision about whether to proceed to a definitive trial. These criteria are outlined in Table 2, and are based on eligibility (rates and reason for ineligibility), recruitment (rates, participant characteristics and reason for declining), retention (rates and reason for withdrawal), and secondary outcome measures (completion rates and reason for missing data).

**Table 2:** Progression criteria.

		GREEN	AMBER	RED
Eligibility: Percentage (%) of people approached with MLTC and frailty who are eligible to participate. Eligibility rate will be calculated as the number of participants meeting inclusion criteria divided by the number of participants approached, presented as a percentage.		≥70%	69−51%	≤50%
Recruitment	% of people living with MLTC and frailty who were eligible and consent to take part in the trial. Recruitment rate will be calculated as the number of people consented divided by the number of people eligible, presented as a percentage.	≥50%	49−29%	≤30%
	% of people living with MLTC and frailty who identify a carer who is subsequently recruited into the trial. Carer recruitment rate will be calculated as the number of carers consented divided by the number of people with MLTC and frailty who consent, presented as a percentage.	≥50%	49−19%	≤20%
	% of participants who are from an ethnic minority background. Ethnic minority recruitment rate will be calculated as the number of people with MLTC and frailty from ethnic minority backgrounds consented divided by the number of people with MLTC and frailty who consent, presented as a percentage.	≥20%	19−6%	≤5%
	% of participants who are from a socioeconomically deprived backgrounds, reflecting rates in the general population [106,107]. Socioeconomic deprivation recruitment rate will be calculated as the number of people with MLTC and frailty from socioeconomically deprived backgrounds consented divided by the number of people with MLTC and frailty who consent, presented as a percentage.	≥30%	29−8%	≤7%
Retention % of participants retained at 24 weeks (providing at least one outcome measure at this timepoint). Retention rate will be calculated as the total number of participants providing at least one outcome measure at 24 weeks divided by the total number of participants randomised, presented as a percentage.		≥70%	69−51%	≤50%
Intervention implementation % of intervention reviews complete, including those delivered by physical activity officers (focusing specifically on the number of intervention reviews completed rather than use of the tools: MyHealthMapp and Steps4Health). Implementation rate will be calculated as the total number of reviews received divided by the total number of reviews intended, presented as a percentage.		≥70%	69−51%	≤50%
Intervention adherence Participants enacts their agreed treatment plan across the intervention period (24 weeks).		3/week on average	2/week on average	1/week on average
Outcome measure completion % of outcome measures completed at 12 and 24 weeks. Completion rates for all secondary outcome measures will be calculated as the number of valid completions divided by the number of people available for follow up at each time point, presented as a percentage.		≥80%	79−59%	≤60%

Red, do not progress to full RCT; Amber, change is required to progress; Green, no issues that may impede success of RCT.

Failure to achieve these pre-established criteria will not necessarily indicate that a definitive trial is not viable. For each criterion, the development of ‘stop’ and ‘go’ thresholds have been pre-specified [105]. The ‘stop’ thresholds indicate when there are issues that may not be resolved, and ‘go’ thresholds when there are no issues that may impede the success of a trial. Results falling between these thresholds indicate that ‘change’ is required, and will allow the research team to identify where there are issues that may be remedied, rendering a definitive RCT viable [90,105].

## Data analysis

### Quantitative data

#### Primary feasibility outcomes.

Primary feasibility outcomes will be reported using descriptive statistics and 95% confidence intervals and presented within a CONSORT diagram [108,109]. The number and percentage of people living with MLTC and frailty and carers who are screened, eligible, ineligible, consented, and retained at 12 and 24 weeks will be reported. To determine outcome acceptability, the proportion of missing data (number, %) will be reported for each secondary outcome. Reasons for missing data will be reported where possible.

#### Secondary outcomes and safety data.

Secondary outcomes at 12 and 24-weeks will be summarised overall and by group to inform decisions about future primary/ co-primary outcomes and potential mechanisms of impact. No imputation will be performed to account for this missing data. Data will be reported using descriptive statistics (mean (SD) or median (IQR) for continuous variables and count (percentage) for categorical variables). The number of SAEs will be reported both overall and by randomised group. Given the study is a feasibility trial, all exploratory analyses will be considered hypothesis generating.

### Intervention fidelity

Data from the fidelity checklist will be reported using descriptive statistics (mean, mean %, range % and number and proportion of analysed interactions where fidelity is high, medium or low). Descriptive statistics will quantify the proportion of standardised components delivered as intended, with total and percentage scores calculated [95]. Fidelity will be classified as high (80–100%), moderate (51–80%), or low (<50%) [95]. For participant-specific and tailored content or BCTs, the mean number delivered per session will be reported [95]. The SDM-Q-9 will be examined descriptively, by reporting at scores at 12- and 24-weeks follow-up, using mean (SD) or median (IQR).

### Adherence

Adherence data will be reported descriptively using number and percentage of interventions reviews received and the number and percentage of participants who access at least one community session. Adherence to the intervention plans and community sessions will be summarised using descriptive statistics, reported as means (SD) or medians (IQR), as appropriate. Data from the 24-hour Physical Behaviour Burden Questionnaire will be also examined descriptively, by reporting mean (SD) or median (IQR) at 12- and 24-weeks follow-up.

### Reach

Baseline characteristics will be summarised overall and by treatment group by number (percentage) for categorical variables and mean (standard deviation) for continuous variables. If data is skewed median (interquartile range) will be reported. No statistical testing will be conducted to compare baseline characteristics between groups, but clinically meaningful differences will be described to support an assessment of reach [108].

### Contextual data

Contextual data from site profile questionnaires will be reported narratively and using descriptive statistics as described above for the other measures.

### Qualitative data

Qualitative data will be collected and analysed concurrently to quantitative analyses, to allow iterative refinement of the intervention to occur [91,110]. The qualitative analysis will adopt an interpretivist, constructivist perspective using reflexive thematic analysis [111,112].

Qualitative data will be analysed using the six stages of reflexive thematic analysis. The research team will read and re-read the data to become familiar with it, making notes and analytic observations. Succinct labels (codes) that capture and evoke important features of the data that might be relevant to addressing the research question will be devised [113]. These will be agreed with the research team through discussion before being applied across the dataset [113]. Multiple rounds of coding may be undertaken and codes revised throughout this process [113]. Codes will then be examined and collated into broader patterns of meaning (potential themes) [113]. Data relating to each candidate theme will be collated. Candidate themes will be checked against the coded data and the entire dataset, to determine fit with the data and ability to address the study aims [113]. Themes will be further developed, combined, or discarded during this phase [113]. A final detailed analysis of each theme will be developed, establishing the scope and focus of each [113]. NVivo (QSR International, 2022, Version 12) will be used to facilitate data organisation [111,112].

It is recognised that codes and themes will be developed through the researchers’ interpretations of the data, shaped by their values, backgrounds, prior experiences, and engagement with participants’ perspectives [112,114]. To enhance reflexivity, researchers will maintain field notes and a reflexive diary after each interview, documenting how their positions may influence all stages of the research process, including interpretation [115].

### Data integration

Following separate qualitative and quantitative data analyses for the trial and process evaluation, all results will be integrated and compared against the progression criteria to facilitate a comprehensive understanding of: the acceptability of the PERSONAL-AGILITY intervention, how the initial intervention programme theory needs to be refined, the feasibility and optimisation of a definitive trial. Integrated analyses will be facilitated by a ‘joint display’, which combines the findings in a tabulated form, alongside the progression criteria previously outlined [116,117]. Following this, decision-making relating to progression to a definitive trial, will be facilitated by the ADePT (A process for Decision-making after Pilot and Feasibility Trials) tool, which was developed to make decision-making regarding feasibility explicit [118].

### Post-trial care

At the final review, participants will be informed that they can continue to use tools identified as part of the intervention following completion of the trial. Control group participants will be offered access to Steps4Health at the end of their final assessment visit.

### Patient and public involvement and engagement

A diverse PPIE group has been convened to guide the design and delivery of the PERSONAL-AGILITY intervention and trial. The group includes twenty-one representatives who have lived experienced of living with (*n* = 13), or caring for someone (*n* = 8), with MLTC and frailty (Table 3). To date the group have contributed to the preparation of the PERSONAL-AGILITY study funding application, the development of the intervention, and the study protocol, including the selection of the outcome measures, and the development of the progression criteria and topic guides. They have also reviewed all participant facing documents and will be involved in interpretation of the trial findings and their subsequent dissemination. A detailed outline of their contribution to date is provided in S4 Table.

**Table 3:** PPIE demographics.

		People with MLTC and frailty (*n* = 13)	Carers (*n* = 8)	Total (*n* = 21)
**Age mean (SD)**		65 (3)	60 (3)	
**Sex N (%)**	**Female**	12 (92)	5 (63)	17 (81)
	**Male**	1 (8)	3 (37)	4 (19)
**Ethnicity N (%)**	**Asian British**	6 (46)	6 (75)	12 (57)
	**Black Caribbean**	3 (23)	1 (12.5)	4 (19)
	**Black British**	2 (15)	0 (0)	2 (9.5)
	**Somalian**	2 (15)	0 (0)	2 (9.5)
	**White British**	0 (0)	1 (12.5)	1 (5)
**Number of LTCs *Median (IQR)***		3 (2-4)	2 (0-3)	
**Clinical Frailty Scale *N (%)***	**Fit (2)**	0 (0)	2 (25)	2 (10)
	**Managing well (3)**	1 (8)	0 (0)	1 (5)
	**Very mildly frail (4)**	1 (8)	4 (50)	5 (24)
	**Mildly frail (5)**	4 (31)	1 (12.5)	5 (24)
	**Moderately frail (6)**	4 (31)	1 (12.5)	5 (24)
	**Severely frail (7)**	2 (15)	0 (0)	2 (9)
**Employment status *N (%)***	**Retired**	9 (69)	3 (37.5)	12 (57)
	**Unemployed**	3 (23)	0 (0)	3 (14)
	**Full time employed**	1 (8)	0 (0)	1 (5)
	**Full time carer**	0 (0)	4 (50)	4 (19)
	**Sick leave**	0 (0)	1 (12.5)	1 (5)
**Highest educational level *N (%)***	**Primary**	2 (15)	1 (12.5)	3 (14)
	**Secondary**	5 (38.5)	3 (37.5)	8 (38)
	**Undergraduate**	1 (8)	2 (25)	3 (14)
	**BTEC**	0 (0)	1 (12.5)	1 (5)
	**No information**	5 (38.5)	1 (12.5)	6 (29)

Abbreviations: MLTC, Multiple long-term conditions.

### Data management

The PERSONAL-AGILITY study team will be responsible for data management for the trial. Source data (medical records, CRFs and patient-reported outcome questionnaires and physical activity officer data collection forms will be stored in a locked cabinet at the Leicester Diabetes Centre and be made available to the appropriate regulatory authorities, the Sponsor, and NHS host organisation as required. Trained members of the PERSONAL-AGILITY team will enter data directly into Redcap (https://www.project-redcap.org), hosted on a University of Leicester virtual ‘LAMP’ server. Quality control checks of the source data and data entered into the trial database will be completed by a second member of the research team and overseen by the CI. All hard and electric copies of data will be identified by a unique study identification code. Electronic data will be password protected, with access restricted to members of the PERSONAL-AGILITY team. A summary of trial data flow is provided in S1 Fig.

### Trial management

The Principal Investigator (PI) and core delivery staff for the PERSONAL-AGILITY team will meet weekly to facilitate day-to-day trial co-ordination and conduct. A trial steering committee (TSC), independent of the sponsor and funder has been established. The TSC consists of the PI, an Independent Chair and statistician (not involved in the trial), and a PPIE representative. The TSC will meet every six months during the trial to monitor study progress, recruitment, and data quality, review participant safety and well-being, advise on any ethical issues or concerns, provide advice on whether the study should continue, be modified, or stopped and help determine whether progression to a full trial is appropriate.

### Trial monitoring

The trial will be conducted in compliance with the approved protocol and will be monitored in accordance with GCP guidelines, the Sponsor, SOPs and any other regulatory requirements. Protocol deviations will be recorded. Major deviations will be reported in line with Sponsor SOPs.

### Trial status

Participant recruitment began in June 2025. At the time of this manuscript (March 2026) 15 participants have been randomised.

### Ethics and dissemination

#### Research ethics approval.

The trial is approved by South Central Oxford B Research Ethics Committee (Ref: 24/SC/0367). The trial protocol has been agreed and approved, and the Principal Investigator has committed to conducting the trial in full compliance with the approved protocol and in accordance with the principles outlined in the current revision of the Declaration of Helsinki (last amended October 2000, with additional footnotes added 2002 and 2004) and the UK Policy Framework for Health and Social Care Research (2017). It will also be conducted according to ICH-GCP relevant regulations. The trial is sponsored by University of Hospitals of Leicester (UHL) NHS Trust (Ref: 73771). The sponsor has reviewed and approved the study protocol and documents. The sponsor will not be involved in data collection, management, analysis, interpretation, or publication. Usual care will resume for participants following completion of the trial, with any compensation for harm provided through sponsor’s insurance. Informed consent will be gathered from all participants prior to enrolment. Amendments to the protocol will be submitted to the Sponsor for review and approval. The chief investigator, in agreement with the Sponsor, will submit the amendment to the appropriate body for approval. Amendments will only be implemented once Sponsor green light is received.

### Dissemination

The anonymised data that support the findings of this study will be openly available in an open access repository. Findings will be published in peer reviewed journals and presented at local and national meetings and conferences, supported by members of the research team. Results will also be communicated to community groups, charities, PPIE groups, and via social media using a variety of formats including short written reports, culturally tailored infographics, and animation videos. All participants will receive a written report and will be able to access the full range of dissemination activities identified above.

## Discussion

This paper outlines the protocol for a RCT which aims to evaluate the feasibility and acceptability of the PERSONAL-AGILITY intervention, including study design and procedures. The trial addresses several importance gaps in the management of MLTC and frailty, including the need for more personalised care, SDM, and the involvement of carers. It incorporates novel technology and links with community groups and services to help overcome challenges in sustainable behaviour change. The findings of this study will inform the feasibility and design of a future definitive RCT of the PERSONAL-AGILITY intervention. A future definitive RCT will establish the effectiveness of the PERSONAL-AGILITY intervention for people living with MLTC and frailty, and their carers, with the potential to inform future care.

## Supporting information

S1 Table Standard Protocol Items Recommendations for Interventional Trials (SPIRIT 2025) guidance. Abbreviations: N/A, not applicable.
(PDF)

S2 Table Template for intervention description and replication (TIDieR) checklist and guidance. Abbreviations: N/A, not applicable; SDM, Shared decision making; MLTC, Multiple long-term conditions.
(PDF)

S3 Table Relevant items from The World Health Organisation Trial Registration Data Set. Abbreviations: SDM, Shared decision making; MLTC, Multiple long-term conditions; NHS, National health service; CFS, Clinical frailty scale; eFI, electronic frailty index; EQ-5D-5L, EuroQol 5 Dimensions 5 Levels; SF-36, 36-Item Short Form Survey.
(PDF)

S4 Table PPIE in the development of the PERSONAL-AGILITY intervention. Abbreviations: PPIE, patient and public involvement; GRIPP2-SF, Guidance for Reporting Involvement of Patients and the Public 2 short form; NIHR, National Institute for Health and Care Research; PROMS, Patient reported outcome measures; MLTC, Multiple long-term condition.
(PDF)

S1 File Full protocol for the study trial (version 4, 25.11.2025). (DOCX)

S1 Fig The PERSONAL-AGILITY Study data flow diagram. Abbreviations: LA, Legal Avenue; LDC, Leicester Diabetes Centre; UHL, University Hospitals of Leicester; MLTC, Multiple long-term conditions; HCP, Health care professional; UOL, University of Leicester.
(TIF)
