# Supplementary material for: Maintaining function and participation through tailored 24-hour movement behaviours for people living with multiple long-term conditions and frailty (The PERSONAL-AGILITY study): Protocol for a randomised controlled feasibility trial
Source: PLoS One. 2026 May 18;21(5):e0348372. doi: 10.1371/journal.pone.0348372 (PMC13183243; doi:10.1371/journal.pone.0348372)
Supplement: S2 Table — Abbreviations: N/A, not applicable; SDM, Shared decision making; MLTC, Multiple long-term conditions. (PDF) [file pone.0348372.s005.pdf]

Item	Item description	Page number
**1. Brief description**	The PERSONAL-AGILITY intervention is a 24-week, theoretically-informed complex intervention that incorporates SDM, personalised goal setting, and technology-based support tailored to individual needs. Developed in accordance with the Medical Research Council framework for complex intervention, and informed by intervention mapping and the Person-Based Approach, the intervention was co-produced with a diverse range of interest-holders. PERSONAL-AGILITY aims to improve participants' 24-hour movement behaviours and, in turn, physical function and quality of life.	13-18
**2. Rationale**	People living with MLTC experience poorer health outcomes and an increased risk of frailty. Exercise interventions in this population often have low adherence, and there is limited evidence of interventions tailored to their needs. Personalised interventions that engage both care recipients and carers, and embed activity within meaningful, community-based contexts, may support sustained behaviour change. To date, no RCTs have evaluated a SDM intervention targeting 24-hour movement behaviours in people with MLTC and frailty.	5-6
**3. What**	**Materials:**<br>• Participants will receive a patient decision aid prior to their first intervention appointment to support engagement and SDM. The decision aid elicits what matters most to participants, supports consideration of the personal benefits and risks of 24-hour movement behaviours, addresses common concerns, considers sources of support, and explains SDM and how it will be used during their appointments.	15-17

	• Participants taking part in a carer dyad will additionally receive a guide designed to support mutual goal setting and effective support.<br>• Participants will be provided with wearable technology (e.g., Fitbits) to support engagement throughout the intervention. Data from wearables, manual entry and patient-reported outcomes will be captured within MyHealthMapp, a web-based platform to support SDM discussions about 24-hour movement behaviours. Participants will be given access to MyHealthMapp.<br>• Participants will also have access to Steps4Health, a web-based physical activity programme offering resources such as activity trackers and interactive content, which can be used ad libitum.<br>• Where feasible, personalised videos summarising individual participant data will be provided prior to intervention appointments to explain results, support reflection on priorities and facilitate appointment preparation.	
	**Procedures:**<br>• The intervention will begin with a holistic assessment using a SDM approach to identify movement behaviours aligned with individual needs and what matters most to participants. The patient decision aid will be used to facilitate these discussions. Information gathered will inform personalised goal setting and the development of an individualised intervention plan.<br>• Participants will be followed up monthly to review success, challenges and setbacks, and to assess whether goals and plans remain meaningful, with intervention plans updated as needed.<br>• Fortnightly interim calls will be used to troubleshoot challenges and support motivation.	13-18

	• At 12-weeks, participants may be linked with local community groups that directly or indirectly support increased physical activity or reduce sedentary behaviour, aligned with participant goals. This linkage will be facilitated by physical activity officers within the local authority.	
**4. Who provided**	The intervention will be delivered by physiotherapists and exercise specialists. All intervention delivery staff will be trained in SDM and behaviour change techniques, and will observe peer-led sessions to support consistent and fidelity of delivery.	14
**5. How**	The intervention will be delivered as one-on-one sessions using a range of methods (in person, by phone, or online). Participants in a carer dyad may choose to attend sessions together or separately.	14-18
**6. Where**	Study and intervention visits will be scheduled around participants' convenience and delivered via multiple methods, including telephone, online, in-person at the Leicester Diabetes Centre, or at participants' homes or in the community where feasible.	15
**7. When and how much**	Intervention participants will be followed up monthly for 24 weeks, with additional fortnightly telephone check-ins. Monthly sessions will be individualised and last approximately 1 hour, while interim calls are expected to last 15-30 minutes.	14-18
**8. Tailoring**	The intervention will be personalised to the participant using MyHealthMapp and SDM to identify what matters most to participants and to weigh the personal benefits and risks of 24-hour movement behaviours.	15-16
**9. Modifications**		N/A
**10. How well**	**Planned:**	29-30

	A mixed methods process evaluation will be conducted by trained research team members to examine intervention implementation (fidelity, adherence, reach), acceptability, and potential mechanisms of impact. Fidelity will be assessed by two trained PERSONAL-AGILITY team members, with inter-rater reliability evaluated to establish agreement. Once sufficient agreement is achieved, subsequent assessments may be conducted by a single team member.	
	**Actual:**	N/A
