# Supplementary material for: Maintaining function and participation through tailored 24-hour movement behaviours for people living with multiple long-term conditions and frailty (The PERSONAL-AGILITY study): Protocol for a randomised controlled feasibility trial
Source: PLoS One. 2026 May 18;21(5):e0348372. doi: 10.1371/journal.pone.0348372 (PMC13183243; doi:10.1371/journal.pone.0348372)
Supplement: S3 Table — Abbreviations: SDM, Shared decision making; MLTC, Multiple long-term conditions; NHS, National health service; CFS, Clinical frailty scale; eFI, electronic frailty index; EQ-5D-5L, EuroQol 5 Dimensions 5 Levels; SF-36, 36-Item Short Form Survey. (PDF) [file pone.0348372.s006.pdf]

Primary registry and trial identifying number	The UK's Clinical Study Registry<br>ISRCTN14362764
Secondary identifying numbers	IRAS: 347586<br>Protocol serial number: NIHR302926, CPMS 65146
Source(s) of monetary or material support	National Institute for Health and Care Research
Primary sponsor	University Hospitals of Leicester NHS Trust<br>Leicester Royal Infirmary<br>Infirmary Square<br>Leicester<br>LE1 5WW<br>United Kingdom
Contact for public queries	The PERSONAL-AGILITY Study<br>Phone: 07483362142 or 0116 2584323<br>Email: uhl-tr.personalagility@nhs.net<br>Leicester Diabetes Centre<br>Leicester General Hospital<br>Gwendolen Road<br>Leicester<br>LE5 4PW<br>United Kingdom
Contact for scientific queries	Dr Hannah Young<br>*Public, Scientific, Principal investigator*<br>Phone: 0116 2584323<br>Email: hy162@leicester.ac.uk

	Leicester Diabetes Centre<br>Leicester General Hospital<br>Gwendolen Road<br>Leicester<br>LE5 4PW<br>United Kingdom
Public title	The PERSONAL-AGILITY study
Scientific title	Maintaining function and participation through tailored 24-hour physical behaviours for people living with multiple conditions and frailty (The PERSONAL-AGILITY study)
Countries of recruitment	United Kingdom<br>England
Health condition(s) or problem(s) studied	Multiple long-term conditions and frailty
Intervention(s)	Current interventions (as of 27/10/2025)<br>Intervention arm: The PERSONAL-AGILITY intervention is designed to support people living with MLTC and frailty using a personalised approach, digital tools and SDM. Wearable devices (e.g., Fitbit, Apple Watch) and web applications (*MyHealthMapp* and *Steps4Health)* are used to collect and monitor activity data and to promote improvements in 24-hour movement behaviours (physical activity, sleep, and sedentary behaviour). The intervention also incorporates face-to-face support, referral to community activities, and accommodates individual preferences for inclusive delivery beyond digital tools.<br>Duration: 24 weeks.<br><br>Usual care arm: Participants receive ongoing standard NHS care and generic information on the 24-hour movement behaviours.<br>Duration: 24 weeks.<br>Participants (and carers, if included) will be randomised 2:1 to the intervention or usual-care arm. A higher allocation to the intervention group increases exposure to, and refinement of, the intervention ahead of a future

	definitive trial. Randomisation will be stratified by frailty status (very mild; mild/moderate/severe) and conducted by a third-party service.<br><br>**Previous interventions**<br>The PERSONAL-AGILITY intervention and usual care were delivered as described above, with the only difference being a **26-week** treatment duration. Randomisation used the same 2:1 ratio and frailty-based stratification but employed variable block sizes of 6 and 9.
Key inclusion criteria	Current inclusion criteria (as of 27/10/2025)<br>All patient and carer participants must be:<br>1. Adults ≥18 years<br>2. Mobile (able to walk 5m including with the use of walking aids)<br>3. Able and willing to provide informed consent<br>4. Able and willing to use digital and online tools with support as part of the intervention.<br><br>People living with MLTC and frailty:<br>1. Living with ≥2 long-term non-communicable conditions<br>2. Living with frailty, defined as a Clinical Frailty Scale score of 4-7 (very mildly frail to severely frail)<br><br>Carers:<br>Providing regular informal care to someone living with MLTC and frailty as defined above for ≥3 months<br>This includes physical and/or emotional support, without professional payment.<br><br>Healthcare professionals:<br>1. Staff delivering the PERSONAL-AGILITY study<br>2. Aged ≥ 18 years<br>3. Able to provide written informed consent<br><br>Previous inclusion criteria:<br>The inclusion criteria is as described above, plus:

	People living with MLTC and frailty must have ≥2 long-term non-communicable conditions, one of which should be Type 2 Diabetes Mellitus (confirmed by medical history or HbA1c ≥6.0% (≥42 mmol/mol) within 3 months of trial enrolment).
Key exclusion criteria	For all participants:<br>1. Unable to provide informed consent<br>2. Unable to communicate in English<br>3. Known contraindications to exercise (as defined by the American College of Sports Medicine) including:<br>a. Unstable cardiac condition<br>b. Active infection<br>c. Significant aortic aneurysm (more than 5.5 cm)<br>d. Any other condition in which the investigator feel exercise may be contraindicated.<br>4.Current participation in competing clinical trial (as determined by study investigator of this trial).<br>5. Significant cognitive impairment (Mini mental state examination score of <24) or unstable psychiatric disorder that limits active participation.<br>6. Serious illness or event with life-expectancy <1year, active malignancy (on chemotherapy/radiotherapy) or other significant illness which, in the opinion of a study clinician, precludes involvement.<br>7. Already regularly engaging in at least 150 minutes of moderate-to-vigorous physical activity per week (self-reported).<br><br>People with MLTC and frailty:<br>• Not frail as defined by an eFI: 0 - 0.12, or a CFS score of 1 - 3 (very fit to managing well).<br>• Not living with 2 or more long-term conditions.<br><br>Carers:<br>• Providing paid/professional care.
Study type	Mixed methods feasibility study
Date of first enrolment	30/04/2025

Sample size	50
Primary outcome(s)	Current primary outcome measures (as of 27/10/2025):<br>Primary outcome measures will assess the feasibility of conducting a future RCT of the PERSONAL-AGILITY intervention.<br>Qualitative data from the process evaluation will be used to contextualise and enhance understanding of these feasibility outcomes.<br><br>1. Eligibility rates (number of eligible participants from screening logs)<br>2. Recruitment rates (number of eligible participants who consented, separated by carer and patient groups from recruitment logs)<br>3. Withdrawal rates (rates of attrition at each stage of the study (screening, consent, randomisation by patient and carer participant group from recruitment logs)<br>4. Outcome measure completion (number of completed measures for all secondary measures from CRF measured at baseline,12 and 24 weeks)<br>5. Rates of uptake, engagement and adherence to each component of the PERSONAL-AGILITY intervention (bespoke diary measured at 12 and 24 weeks).<br>6. Burden associated with the intervention (24-hour Physical Behaviour Burden Questionnaire measured at 12 and 24 weeks).<br>7. The participants' perception of the shared decision-making process (Shared decision-making questionnaire measured at 12 and 24 weeks).<br><br>Previous primary outcome measures:<br>The primary outcome measures are the same as described above, with the only difference being that they were previously collected at 12 and **26-weeks, rather than 12 and 24 weeks.**
Key secondary outcome(s)	Semi-structured interviews will be used to explore:<br>1. The acceptability of key trial procedures and outcome measures, including any unintended harms or consequences.

	2. The acceptability of the intervention, including:<br>2.1. The influence of interactions between participants taking part as a dyad.<br>2.2. Experiences of social prescribing and engagement with community groups.<br>2.3. Barriers and facilitators to engagement.<br>3. Potential mechanisms of intervention impact.<br>4. How contextual factors shaped intervention delivery and impact, including any unintended consequences.<br>5. Participants' perceptions of PERSONAL-AGILITY, including views on delivery and need for adaptations.<br>6. How contextual influences hindered or facilitated implementation.<br>Quantitative data will be analysed using descriptive statistics, and qualitative data will be analysed using a reflexive thematic approach.<br><br>Added 27/10/2025:<br>The following measures will be collected at baseline, 12 weeks, and 24 weeks:<br>1. Body composition (bioimpedance analysis).<br>2. Blood pressure (automated sphygmomanometer).<br>3. Handgrip strength (hand dynamometry).<br>4. Physical function (Short Physical Performance Battery).<br>5. Balance (Berg Balance Scale).<br>6. 24-hour movement behaviours, including stepping, sweating, strengthening, sitting, and sleep (accelerometers and inclinometers).<br>7. Quality of life (EQ-5D-5L and SF-36).<br>8. Life participation (Late Life Function and Disability Instrument).<br>9. Symptoms (Patient Outcome Scale).<br>10. Goal attainment (Goal Attainment Scale).<br>11. Care burden for carers (Zarit Burden Interview).
Ethics review	Approved 12/12/2024, South Central - Oxford B REC (Health Research Authority, 2 Redman Place, Stratford, London, E20 1JQ, United Kingdom; +44 (0)207 104 8134, +44 (0)207 104 8019; oxfordb.rec@hra.nhs.uk), ref: 24/SC/0367

Individual trial participant data sharing statement	The datasets generated and/or analysed during the current study will be available on request from Dr Hannah Young (hannah.young44@nhs.net)
Summary of amendments	The following changes were made to the study record (27/10/2025):<br>1. Participant information sheet uploaded.<br>2. Contact details, study objectives, interventions, primary and secondary outcome measures, and inclusion criteria were updated.<br>3. The public title was changed from 'Tailored 24-hour physical behaviours for people living with multiple cardiorenal metabolic conditions and frailty' to 'Tailored 24-hour physical behaviours for people living with multiple conditions and frailty'.<br>4. The scientific title was changed from 'Maintaining function and participation through tailored 24-hour physical behaviours for people living with multiple cardiorenal metabolic conditions and frailty' to 'Maintaining function and participation through tailored 24-hour physical behaviours for people living with multiple conditions and frailty'.<br>25/03/2025: The recruitment start date was changed from 31/03/2025 to 30/04/2025.<br>28/02/2025: The recruitment start date was changed from 28/02/2025 to 31/03/2025.<br>03/02/2025: Participant information sheet uploaded. The recruitment start date was changed from 30/01/2025 to 28/02/2025.<br>15/01/2025: Ethics approval details added.<br>24/09/2024: Trial's existence confirmed by the National Institute for Health and Care Research (NIHR) (UK).
