# Supplementary material for: Maintaining function and participation through tailored 24-hour movement behaviours for people living with multiple long-term conditions and frailty (The PERSONAL-AGILITY study): Protocol for a randomised controlled feasibility trial
Source: PLoS One. 2026 May 18;21(5):e0348372. doi: 10.1371/journal.pone.0348372 (PMC13183243; doi:10.1371/journal.pone.0348372)
Supplement: S4 Table — Abbreviations: PPIE, patient and public involvement; GRIPP2-SF, Guidance for Reporting Involvement of Patients and the Public 2 short form; NIHR, National Institute for Health and Care Research; PROMS, Patient reported outcome measures; MLTC, Multiple long-term condition. (PDF) [file pone.0348372.s001.pdf]

Section and topic	Item
**1. Aim**	To guide the development and delivery of research activities in the PERSONAL-AGILITY trial and intervention.
**2. Methods**	**Recruitment:** The PPIE strategy development was informed by the NIHR Standards for Public Involvement and guidance on involving carers in research. To ensure inclusivity and successfully engage with underrepresented groups, the research team is working with the community engagement officer form the Centre for Ethnic Health Research and BRC direction of inclusion. They, alongside the research team have been to events in the community to overcome barriers to engagement and maintain partnerships. They will continue to facilitate initial and ongoing recruitment to PPIE.<br><br>**PPI meetings:** The PPI group meetings are held regularly, with additional meetings scheduled as needed. Members requested the option to opt-in and out of involvement due to changes in availability and health. All members of the group are kept informed of discussions/decisions made, consulted individually where needed and offered the opportunity to input as they wish. From the outset, ground rules have been developed together with PPI members to confirm roles, responsibilities and behaviours.<br><br>PPIE meetings and activities are flexible and pragmatic to ensure activities are meaningful and inclusive to participants, including adaptation of materials for those with sensory impairments where possible. Meetings are offered at different days/times and in a range of formats, including accessible locations, to meet the needs and preferences of a diverse range of individuals.<br><br>Members are and reimbursed for their time and expertise in line with NIHR guidance. Building relationships with interest-holders is central and will be maintained through regular communication and social events where possible. Mutual sharing of knowledge and joint decision making is supported and encouraged.<br><br>**Evaluation**: Reflection logs are completed after every activity to promote evaluation of activities and their impact, guiding necessary changes to the proposed strategy. The Public and Patient Engagement Evaluation Tool will be used annually.

	**Participants:** To date, the PPIE group includes twenty-one representatives who have lived experienced of living with (n=13), or caring for someone (n=8), with MLTC and frailty (Table 4).
**3. Results to date**	The PPIE group have been actively involved in preparation for the funding application, pre-award, and were involved in the development of the protocol. To date, PPIE have contributed to the study development in several ways, including:<br>• Reviewed all patient facing documentations and lay summaries<br>• Development of a priori progression criteria, alongside the trial team<br>• Co-designing the dissemination strategy<br>• Providing feedback on proposed outcome measures to ensure they were meaningful and acceptable<br>• Option for home and community-based testing to help make the intervention more inclusive where possible<br>• Simple user guides for the online tools to help reduce the risk of digital exclusion<br>• Offered guidance around the development of preliminary semi-structured topic guides<br>Early engagement with PPIE members also highlighted potential practical barriers to participation, such as potential burden of taking part and scheduling of visits around patients' complex lives. To help reduce the potential burden of the trial and intervention the following strategies were developed through discussions with PPI:<br>• Option to opt out of the interviews<br>• Study and intervention visits to be arranged around participant's convenience, using a range of methods (telephone, online, in person, plus home and community visits where possible)<br>• Offering participants support to complete PROMs and organising in the order of priority of the research team<br>• Only selecting intervention measures that are relevant and important to participants<br>Future activities as the trial progresses include contributing to the interpretation of study findings and involvement in decision-making regarding progress to a future definitive trial.
