# Supplementary material for: Maintaining function and participation through tailored 24-hour movement behaviours for people living with multiple long-term conditions and frailty (The PERSONAL-AGILITY study): Protocol for a randomised controlled feasibility trial
Source: PLoS One. 2026 May 18;21(5):e0348372. doi: 10.1371/journal.pone.0348372 (PMC13183243; doi:10.1371/journal.pone.0348372)
Supplement: S1 File — (DOCX) [file pone.0348372.s002.docx]

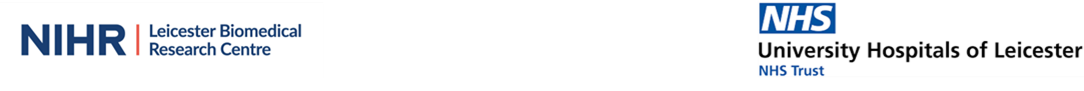

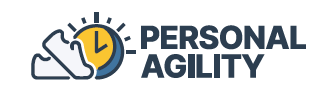


**PROTOCOL INFORMATION**

**FULL TITLE OF THE TRIAL:**

Maintaining function and participation through tailored 24-hour physical behaviours for people living with multiple conditions and frailty.

**SHORT TRIAL TITLE / ACRONYM:**

The PERSONAL-AGILITY study

**PROTOCOL VERSION NUMBER AND DATE**: 4.0 25.11.2025

**IRAS NUMBER:** 347586

**SPONSOR:** University Hospitals of Leicester NHS Trust

**SPONSOR REFERENCE NUMBER:** 173771

**TRIAL REGISTRATION**: ISRCTN14362764

**FUNDER(S):**

This study is funded by the National Institute for Health and Care Research (NIHR) under its Advanced fellowship programme (NIHR302926). The views expressed are those of the author(s) and not necessarily those of the NIHR or Department of Health and Social Care.

The study is part of the following programme: Maintaining function and participation through whole-system, tailored 24-hour physical behaviour programme for people living with multiple conditions and frailty (the PERSONAL-AGILITY study)

**Declaration of HRA protocol template use:**

This protocol has regard for the Health Research Authority (HRA) guidance and order of content, in line with Version 1.1 (March 2016) of the HRA Protocol Development Tool.

**Confidentiality Statement:**

All information contained within this protocol is regarded as, and must be kept, confidential. No part of it may be disclosed to anyone other than the Sponsor, the Investigator Team, host NHS Trust, regulatory authorities and members of the Research Ethics Committee, by any Receiving Party to any Third Party, at any time, or in any form without the express written permission from the Chief Author/Investigator and / or Sponsor.

**SIGNATURE PAGE**

The undersigned confirm that the following protocol has been agreed and accepted and that the CI agrees to conduct the study in compliance with the approved protocol and will adhere to the principles outlined in the GCP guidelines, the Sponsor’s (and any other relevant) SOPs, and other regulatory requirements.

I agree to ensure that the confidential information contained in this document will not be used for any other purpose other than the evaluation or conduct of the clinical investigation without the prior written consent of the Sponsor.

I also confirm that I will make the findings of the study publicly available through publication or other dissemination tools without any unnecessary delay and that an honest, accurate and transparent account of the trial will be given. Any discrepancies and serious breaches of GCP from the study as planned in this protocol will be explained.

| Sponsor: | |
| --- | --- |
| Signature: | Date: |
| Name (please print): |  |
| Position: |  |
| Principal Investigator | |
| Signature: | Date: |
| Name: Dr Hannah Young |  |

**KEY STUDY CONTACTS**

| Principal Investigator | Dr Hannah Young  Diabetes Research Centre  Leicester Diabetes Centre  Leicester General Hospital  Gwendolen Road  Leicester, LE5 4PW  Tel: 0116 258 4323  Email: [hannah.young@uhl-tr.nhs.uk](mailto:hannah.young@uhl-tr.nhs.uk) |
| --- | --- |
| Main Trial Contact | Dr Hannah Young  Diabetes Research Centre  Leicester Diabetes Centre  Leicester General Hospital  Gwendolen Road  Leicester, LE5 4PW  Tel: 0116 258 4323  Email: [hannah.young@uhl-tr.nhs.uk](mailto:hannah.young@uhl-tr.nhs.uk) |
| Sponsor | Mrs Carolyn Maloney  Head of Research Operations  Research Office. Trust Headquarters. Level 3 Balmoral Building.  Leicester Royal Infirmary. Infirmary Square.  Leicester LE1 5WW  Tel: 0116 258 4109  Email: [uhlsponsor@uhl-tr.nhs.uk](mailto:uhlsponsor@uhl-tr.nhs.uk) |
| Funder(s) | NIHR Advanced Fellowship (NIHR 302926)  Martha Thomas’ studentship is supported by the Leicester Biomedical Research Centre |
| Funder start and end date(s) | May 2023- April 2028 |

**PROTOCOL CONTRIBUTORS**

| Name | Position | Role on study | Organisation |
| --- | --- | --- | --- |
| Dr Hannah Young | Specialist research physiotherapist  Honorary Associate Professor | Grant lead applicant  Chief Investigator | University Hospitals of Leicester; Leicester Diabetes Centre  University of Leicester, Diabetes Research Centre |
| Professor Melanie Davies | Professor of Diabetes Medicine | Mentor, collaborator, and co-author | University Hospitals of Leicester; Leicester Diabetes Centre  University of Leicester, Diabetes Research Centre |
| Dr Louisa Herring | Lead Research Associate | Co-author | University Hospitals of Leicester; Leicester Diabetes Centre |
| Martha Thomas | PhD student | Co-author | University of Leicester;  Diabetes Research Centre |
| Professor Laura Gray | Professor of Medical Statistics | Mentor, collaborator, and co-author | University of Leicester; Department of Population Health Sciences |
| Professor Avan Sayer | Professor of Geriatric Medicine | Mentor, collaborator, and co-author | NIHR Newcastle Biomedical Research Centre  Newcastle University |
| Professor Sharlene Greenwood | Consultant Physiotherapist | Mentor, collaborator, and co-author | Kings College Hospital London |
| Dr Adwoa Parker | Assistant Professor | Collaborator and co-author | University of York, Health Sciences |
| Dr Alex Rowlands | Associate Professor | Collaborator and co-author | University of Leicester; Department of Population Health Sciences |
| Dr Jemma Hawkins | Senior Lecturer | Collaborator and co-author | DECIPHer, School of Social Sciences, Cardiff University |
| Professor Matthew Maddocks | Professor of Health Services Research & Rehabilitation | Collaborator and co-author | Cicely Saunders Institute of Palliative Care, Policy & Rehabilitation, Kings College London. |
| Professor Tom Yates | Professor of Physical Activity, Sedentary Behaviour and Health | Collaborator and co-author | University of Leicester, Diabetes Research Centre |
| Dr Leslie Borrill | Medical Director of the Charnwood GP Network | Collaborator and co-author | Bridge Street Medical Practice, Loughborough |
| Professor Sam Seidu | Professor in Primary Care Diabetes and Cardio-metabolic Medicine | Collaborator and co-author | University Hospitals of Leicester; Leicester Diabetes Centre  University of Leicester; Diabetes Research Centre |
| Dr Michelle Hadjiconstantinou | Research Fellow | Collaborator and co-author | University Hospitals of Leicester; Leicester Diabetes Centre  University of Leicester; Diabetes Research Centre |
| Professor Kamlesh Khunti | Professor of Primary Care, Diabetes and Vascular Medicine | Collaborator and co-author | University Hospitals of Leicester; Leicester Diabetes Centre  University of Leicester; Diabetes Research Centre |

| NIHR Portfolio adopted? | Yes |
| --- | --- |

**Contents**

[1 Background and rationale 21](#_Toc174458148)

[2 Research objectives and outcome measures 25](#_Toc174458149)

[2.1 Primary objectives 25](#_Toc174458150)

[2.2 Secondary objectives 25](#_Toc174458151)

[2.3 Outcome measures 26](#_Toc174458152)

[2.3.1 Primary 26](#_Toc174458153)

[2.3.2 Secondary 26](#_Toc174458154)

[3 Trial design 27](#_Toc174458155)

[4 Trial setting 29](#_Toc174458156)

[5 Participant eligibility criteria 29](#_Toc174458157)

[5.1 Inclusion criteria 29](#_Toc174458158)

[5.2 Exclusion criteria 31](#_Toc174458159)

[6 Trial procedures 32](#_Toc174458160)

[6.1 Schedule of procedures 33](#_Toc174458161)

[6.2 Recruitment 34](#_Toc174458162)

[6.2.1 Participant identification 34](#_Toc174458163)

[6.2.2 Recruitment from primary and secondary care 34](#_Toc174458164)

[6.2.3 Recruitment through participant and volunteer database 35](#_Toc174458165)

[6.2.4 Recruitment through community engagement 35](#_Toc174458166)

[6.2.5 Recruitment through other methods 35](#_Toc174458167)

[6.2.6 Recruitment procedures 36](#_Toc174458168)

[6.2.7 Reimbursement 37](#_Toc174458169)

[6.2.8 Consent 37](#_Toc174458170)

[6.3 Screening 39](#_Toc174458171)

[6.4 Carer consent 40](#_Toc174458172)

[6.5 Baseline data 40](#_Toc174458173)

[6.6 Randomisation 42](#_Toc174458174)

[6.6.1 Method of implementing the randomisation/allocation sequence 42](#_Toc174458175)

[6.7 Blinding 43](#_Toc174458176)

[6.8 Trial intervention 43](#_Toc174458177)

[6.8.1 Technology 43](#_Toc174458178)

[6.9 Delivery of care 46](#_Toc174458179)

[6.10 Usual care 47](#_Toc174458180)

[7 Trial assessments 47](#_Toc174458181)

[7.1 Trial visit schedule 47](#_Toc174458182)

[7.1.1 Feasibility of trial methodology 48](#_Toc174458183)

[7.1.2 Acceptability of trial methodology and intervention 49](#_Toc174458184)

[7.2 Secondary outcomes 50](#_Toc174458185)

[7.2.1 Physiological measures 51](#_Toc174458186)

[7.2.2 Physical function 52](#_Toc174458187)

[7.2.3 Accelerometer and inclinometer measured physical behaviours 53](#_Toc174458188)

[7.2.4 Patient reported outcome measures (PROMS) 54](#_Toc174458189)

[7.3 Mixed methods process evaluation 55](#_Toc174458190)

[7.3.1 Participant eligibility criteria for the mixed-methods process evaluation 59](#_Toc174458191)

[7.3.2 Implementation data collection 59](#_Toc174458192)

[7.4 Qualitative interview data collection 61](#_Toc174458193)

[7.4.1 Sample size 61](#_Toc174458194)

[7.4.2 Sampling technique 62](#_Toc174458195)

[7.5 Recruitment and consent 64](#_Toc174458196)

[7.5.1 People living with MLTCs and frailty, and carer participants 64](#_Toc174458197)

[7.5.2 Healthcare professionals 64](#_Toc174458198)

[7.6 Consent procedures for the interviews 64](#_Toc174458199)

[7.7 Qualitative data collection for both people with MLTCs and frailty, and healthcare professionals 65](#_Toc174458200)

[7.8 Withdrawal criteria 67](#_Toc174458201)

[7.9 Assessment and management of risk 69](#_Toc174458202)

[7.9.1 Outcome testing 69](#_Toc174458203)

[7.9.2 Intervention 70](#_Toc174458204)

[7.9.3 Participation burden 71](#_Toc174458205)

[7.9.4 Mixed methods process evaluation 72](#_Toc174458206)

[7.10 End of trial 73](#_Toc174458207)

[8 Recording and reporting of serious adverse events (SAEs) 74](#_Toc174458208)

[8.1 Definitions 74](#_Toc174458209)

[8.2 Reporting procedures for all Adverse Events 75](#_Toc174458210)

[8.3 Expected Adverse Events and Serious Adverse Events 75](#_Toc174458211)

[8.4 Reporting Procedures for Serious Adverse Events 76](#_Toc174458212)

[9 Statistics and data analysis 77](#_Toc174458213)

[9.1 Sample size calculation 77](#_Toc174458214)

[9.2 Summary of baseline data and flow of participants 77](#_Toc174458215)

[9.3 Primary trial outcome analysis 77](#_Toc174458216)

[9.4 Progression criteria 78](#_Toc174458217)

[9.5 Secondary trial outcome analysis 81](#_Toc174458218)

[9.6 Analysis of the mixed methods process evaluation 81](#_Toc174458219)

[9.6.1 Quantitative analysis 81](#_Toc174458220)

[9.6.2 Qualitative analysis 82](#_Toc174458221)

[9.7 Mixed methods analysis 83](#_Toc174458222)

[10 Data management 84](#_Toc174458223)

[10.1 Data Flow Diagram 84](#_Toc174458224)

[10.2 Data collection and management 86](#_Toc174458225)

[10.3 RED Cap (trial database) 87](#_Toc174458226)

[10.4 Access to data 87](#_Toc174458227)

[10.5 Archiving 88](#_Toc174458228)

[11 Monitoring, order and inspection 88](#_Toc174458229)

[12 Ethical and regulatory considerations 89](#_Toc174458230)

[12.1 Research Ethics Committee (REC) review and reports 89](#_Toc174458231)

[13 Peer review 90](#_Toc174458232)

[14 Lived experience group involvement 90](#_Toc174458233)

[14.1 Overview 90](#_Toc174458234)

[14.2 PPI/E strategy 90](#_Toc174458235)

[14.3 Evaluation 92](#_Toc174458236)

[15 Regulatory compliance 92](#_Toc174458237)

[16 Protocol compliance 93](#_Toc174458238)

[17 Data protection and confidentiality 93](#_Toc174458239)

[18 Indemnity 95](#_Toc174458240)

[19 Post trial care 96](#_Toc174458241)

[20 Access to final trial data set 96](#_Toc174458242)

[21 Dissemination policy 96](#_Toc174458243)

[22 Authorship eligibility guidelines and any intended use of professional writers 97](#_Toc174458244)

[23 References 97](#_Toc174458245)

[24 Appendices 105](#_Toc174458246)

**LIST OF ABBREVIATIONS**

| ACG | Adjusted Clinical Groups |
| --- | --- |
| ACSM | American College of Sports Medicine |
| AE | Adverse Event |
| BMI | Body Mass Index |
| BRC | Biomedical Research Centre |
| CEHR | Centre for Ethnic Health Research |
| CFS | Clinical Frailty Score |
| CI | Chief Investigator |
| CONSORT | Consolidated Standards of Reporting Trials |
| CRFs | Case Report Forms |
| CTIMPs | Clinical Trials of Investigational Medicinal Products |
| eFI | Electronic frailty index |
| EM-ARC | East Midlands Applied Research Collaborative |
| EQ-5D-5L | EuroQol-5 Dimensions |
| GAS | Goal Attainment Scale |
| GCP | Good Clinical Practice |
| GDPR | General Data Protection Regulation |
| GP | General Practitioner |
|  |  |
| HCP | Health Care Professional |
| HRA | Health Research Authority |
| ICH-GCP | International Committee on Harmonization of Good Clinical Practice |
| IMD | Index of multiple deprivation |
| IQR | Interquartile Range |
| Late-life FDI | Late Life Function and Disability Instrument |
| LDC | Leicester Diabetes Centre |
| MLTCS | Multiple Long-Term Conditions |
| NHS | National Health Service |
| NICE | National Institute for Health and Care Excellence |
| NIHR | National Institute for Health and Care Research |
| NPT | Normalisation Process Theory |
| PI | Principal Investigator |
| PIC | Participant Identification Centre |
| PIS | Participant Information Sheet |
| POS | Patient Outcome Scale |
| PPI/E | Patient and Public Involvement/Engagement |
| PROM | Patient reported outcome measure |
| QALY | Quality adjusted life years |
| QoL | Quality of Life |
| RAG | Red, amber, green |
| RCT | Randomised Controlled Trial |
| RDN | Research Delivery Network |
| REC | Research Ethics Committee |
| SAEs | Serious Adverse Events |
| SD | Standard Deviation |
| SDM | Shared Decision Making |
| SF-36 | The 36-Item Short Form Survey |
| SMS | Short message service |
| SOPs | Standard Operating Procedures |
| SPPB | Short Physical Performance Battery |
| TMF | Trial master file |
| TSG | Trial Steering Group |
| UHL | University Hospitals of Leicester |
| WHO | World Health Organisation |

**KEY WORDS**

Feasibility, frailty, carers, multiple long-term conditions, physical activity, sedentary behaviour, sleep

**TRIAL SUMMARY**

| Trial Title | Maintaining function and participation through tailored 24-hour physical behaviours for people living with multiple conditions and frailty. | |
| --- | --- | --- |
| Short title | The PERSONAL-AGILITY study | |
| Study Acronym | N/A | |
| Trial Design | Feasibility randomised controlled trial (RCT), with a mixed-methods process evaluation, to determine the feasibility and acceptability of a complex intervention that uses a range of approaches tailored to individual needs, with the use of novel technology. | |
| Trial Participants | Individuals aged ≥18 years living with multiple long-term conditions (MLTCs) and frailty and their carers (optional) | |
| Planned Sample Size | **Feasibility trial**  *n*=50 people living with MLTCs and frailty and one main carer (up to *n*=50)  **Process evaluation**   - Implementation fidelity with 10% of sessions - Interviews with: - ~*n*=15 people living with MLTCs and frailty from the intervention group - ~*n*=15 carers from the intervention group - ~*n*=15 Health and care professionals (physical activity officers and other link or community providers involved in intervention delivery) | |
| Follow-up duration | 24 weeks | |
| Planned trial period | 36 months | |
|  | Objectives | End Points / Outcome Measures |
| Primary | To establish the feasibility and acceptability of a future randomised controlled trial (RCT)investigating the effectiveness the PERSONAL-AGILITY intervention | - Eligibility rate - Recruitment rate - Retention rate - Outcome measure completion rates |
| Secondary | To establish the acceptability of the PERSONAL-AGILITY intervention for people living with MLTCs, their carers, and health and care professionals involved in the intervention, including key constructs relating to future implementation | - Rates of uptake and engagement with each component of the PERSONAL-AGILITY intervention - Rates of adherence to the intervention - The 24-hour Physical Behaviours Burden Questionnaire (Intervention group only) |
|  | To test the potential effectiveness of the PERSONAL-AGILITY intervention for people living with MLTCS, their carers, compared to usual care, on a range of secondary outcomes at 12 and 24 weeks. | - Bioimpedance analysis - Blood pressure - Handgrip strength - Short Physical Performance Battery - The Berg Balance Scale - Accelerometer and inclinometer measurement of the 24-hour physical behaviours (stepping, sweating, strengthening, sitting and sleep) - EQ-5D-5L - Short Form-36 - The Late Life Function and Disability Instrument - The Patient Outcome Scale - Goal Attainment Scale - Zarit Burden Interview (carers only) |
|  | To evaluate the implementation of the PERSONAL-AGILITY intervention, its potential mechanisms of impact and the influence of context | - Implementation fidelity assessment - Intervention process data - The 9-item shared decision-making questionnaire (intervention group only) |

**FUNDING AND SUPPORT IN KIND**

| FUNDER(S) | FINANCIAL AND NON-FINANCIAL SUPPORT GIVEN |
| --- | --- |
| NIHR personal fellowship  Tel: 020 8843 8056  Email: academy-awards@nihr.ac.uk | As detailed in the award letter. The funder will be responsible for funding the study but not study conduct, data analysis and interpretation, manuscript writing, and dissemination. |

**ROLE OF TRIAL SPONSOR**

The sponsor of this research is the University of Hospitals of Leicester (UHL). UHL is registered as a research sponsor with the Department of Health and routinely takes responsibility as sponsor for research activities within the NHS. The Sponsor will not be part of the study conduct, data analysis and interpretation, manuscript writing, and dissemination of results.

**ROLE OF COLLABORATOR(S)**

The collaborators will work collectively with the trial Principal Investigator (PI) throughout the scientific development and/or delivery of the proposed work.

**ROLES AND RESPONSIBILITIES OF TRIAL MANAGEMENT COMMITTEES/GROUPS & INDIVIDUALS**

**Trial Steering Group**

A trial steering group (TSG) consisting of the PI, core delivery staff (including trial statistician) and other co-investigators and collaborators and two patient and public involvement/engagement representatives (one carer and one person living with multiple long-term conditions and frailty) will meet approximately every 3 months or more regularly as required to oversee and coordinate delivery of the trial.

**Lived experience involvement group**

A lived experience involvement group have been actively involved throughout the inception of the work and will continue to be engaged at all stages of the research. The involvement of people with MLTCs and frailty and carers is described in detail within section 14.

**TRIAL FLOW CHART**


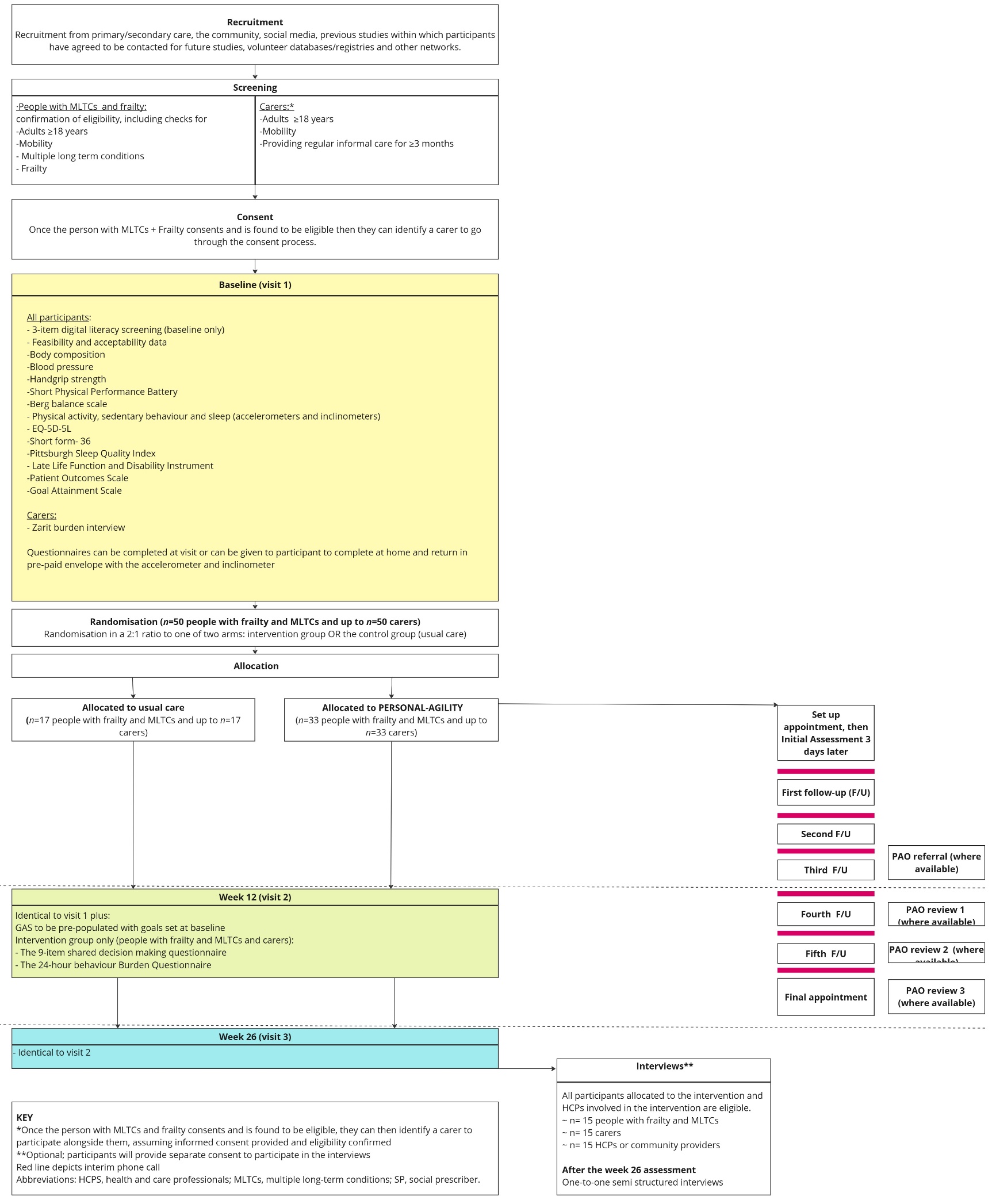


Figure 1. PERSONAL-AGILITY trial overview.

# Background and rationale

An 86% increase in the number of people living with two or more chronic conditions^1^ (multiple long-term conditions, MLTCs) is predicted by 2035^2^ ^3^. People with MLTCs are more likely to be admitted to hospital, stay for a longer duration and die prematurely, compared to those with a single LTC^4^. The burden of treatment is also greatly increased but often overlooked by health and care services ^5^. People living with MLTCs are also more likely to be frail, a multidimensional syndrome of decreased physiological reserve^6^, and are particularly vulnerable to poor outcomes including low physical function ^7,8,^ loss of independence and social participation^3, 7^. Frailty also elevates the burden of MLTCs due to increased hospitalisation and institutionalisation^9^. Consequently costing the NHS £5.8 billion annually^10^. £7 out of every £10 of England’s health and social care expenditure is spent on people with long-term conditions, with costs increasing with each additional condition present. In addition, inactivity during the COVID-19 pandemic has led to a predicted additional cost of £211 million per year to health and social care^11^.

Physical inactivity and sarcopenia are hallmarks of both MLTCs and frailty, and are associated with poor physical function, making them primary targets for intervention^12^,^13^. Current rehabilitation programmes are supervised, in-centre (face-to-face), single-disease services ^14^. They cover limited conditions^15^, may be burdensome for people with MLTCs ^16^ and show mixed effects in this group^17, 18,19^. Programmes are also rarely tailored for individuals who are also living with frailty. Indeed, some studies exclude frail participants, leading to unrepresentative samples and ongoing uncertainty about the risk: benefit ratio for people living with MLTCs and frailty^20^. One review by De Vries N et al, from 2012 has examined the effectiveness of exercise for older people living with MLTCs and frailty, concluding that multicomponent exercise programmes improve physical functioning^21^. Contemporary research indicates that the demographics of people living with MLTCs and frailty are changing, with accelerated rates of both phenomena in younger populations with emerging health issues^22, 23, 24^. Focusing upon impairments rather than diseases, and considering frailty as an interacting indicator of risk across a range of ages, may lead to more targeted, person-centred rehabilitation^25,26^. In addition, current research has predominantly focused on structured exercise programmes for people living with MLTCs and frailty^27^. Adherence to exercise is variable (~50%) in this population ^12^ and up to 90% of this group are unable to achieve current physical activity recommendations for the general population^28^. Symptom burden is high, with chronic pain affecting ~70%, fatigue ~50%, and breathlessness ~40%^29^. High symptom burden is associated with lower physical activity adherence and reduced function^29^.

The importance of physical behaviours over the 24-hour day are now more widely recognised after their inclusion in Canadian physical activity guidelines for their general population ^30^ and international recommendations for the management of Type 2 Diabetes^31^. The 24-hour physical behaviours encompass physical activity, sedentary behaviour and sleep^30^, sometimes termed the 5S’s (stepping, sweating, strengthening, sitting and sleeping) ^31^. Improvements in the 24-hour physical behaviours have been associated with a reduced risk of morbidity and mortality^30^. For example, light intensity activities, consistent with daily activities such as casual walking and household chores, have been associated with improved cardiometabolic health and reduced risk of mortality^32^. Additionally, prolonged sitting time ^33^ and sleeping too little (<6 hours) or too much (>8 hours) have been associated with unfavourable health outcomes in people with long-term conditions^34^. Frailty is also associated with longer (>8 hours) or shorter (<6 hours) sleep durations compared to those with a normal sleep duration (6-8 hours)^35^. Moreover, sedentary behaviours have also been linked to a higher risk of frailty and higher severity of frailty^36^.

The World Health Organisation (WHO) also recommends a holistic approach to increasing physical activity, combining individual, interpersonal and social change^28, 37,38^. Adopting a flexible approach, which incorporates all of the 24-hour behaviours, may be more acceptable than a traditional structured exercise programme, and lead to more sustained changes in health behaviours, but has yet to be tested^19, 39^. Traditional structured exercise may be unrealistic for individuals who are currently physically inactive or live with MLTCs. person centred interventions that incorporate the 24-hour physical behaviours provide more options for people with MLTCs and frailty to improve their health, and give participants a greater say in their own care This approach is supported by the National Institute for Health and Care Excellence (NICE) guidelines that state that shared decision making (SDM) is an essential feature of good quality healthcare^40^. Shared decision making is a collaborative process in which a person, along with their family and carer, should work together with a HCP to make decisions about care ^40^.

At the interpersonal level, as the number of conditions a person is living with increases, so too can their reliance on the people around them. Therefore, it is important to also consider the health of those caring for people living with MLTCs. Since the COVID-19 pandemic, the number of informal carers in the UK has increased by 4.5 million^41^, with 30% reporting poor health ^42^. Informal care is valued at £193 billion per annum, making carer health and wellbeing economically important to society^42^. Informal carers positively influence care recipients physical activity levels including their uptake of, and adherence to, rehabilitation^38^, indicating that the involvement of carers in an intervention may be important.

Whilst there are many positive effects of caregiving ^43,44,^ it is also associated with a range of negative impacts on physical and mental health, quality of life (QoL) and burden perception ^43,44, 45, 46^. MLTCs and frailty often occur within families ^47^, particularly among minority ethnic groups^48^, linked to inactivity ^49^, and lack of opportunity due to caregiving ^44^. Indeed, carers face a 16% higher risk of developing MLTCs than non-carers ^50^  and caring for a spouse living with frailty is an independent risk factor for frailty among older adults ^51, 52^. Given that carers and care-recipients influence each other’s behaviours^53, 54,55, 56^ and that carers are more likely to be physically inactive^57^ interventions involving and including carers as participants who will also benefit are relevant. Such interventions improve carer psychological wellbeing ^58, 59, 60, 61,^  physical health^60, 61,^ sleep^59^, habitual physical activity ^59^ and QoL ^59^, whilst those which only involve caregivers to enhance adherence may inadvertently add to this burden^27^.

Interventions which use, or are facilitated by digital tools and videos may offer a clinically and cost-effective means of addressing barriers to accessing support for physical health behaviours ^62, 63^. Increasingly they are able to offer a level of personalisation which is engaging ^62^ and can increase physical and health literacy and promote greater change in health behaviours when compared to generic information ^64, 65^. Digital tools and videos may also support shared decision making and promote person-centred care by enabling healthcare professional to monitor personal health data and use it to discuss the individual needs of those they care for^66, 64, 65^. To date, few digital tools relating to the 24 physical health behaviours appear to have been developed ^67, 68^, or to have been developed with the specific needs of people living with MLTCs and their carers in mind^69^, which may be important for engagement and effectiveness^70^.

Finally, maintaining improvements via repeated courses of intervention is limited^71^. Sustaining functional gains as a ‘by product’ of meaningful, enjoyable, and culturally appropriate activity may be more effective^38, 72^. Therefore, at the community level, harnessing local assets via social prescribing and local authority leisure support may help maintain the functional gains achieved through increased physical activity. Such an approach may also have beneficial effects on social participation, which existing rehabilitation programmes do not consistently positively impact ^73^.To date, no studies have combined a physical activity intervention with this type of approach in this population.

Therefore, a holistic approach, which combines:

- Individualised, shared decision making, allowing adapted of the focus of the intervention according to needs, preference and health status
- Interpersonal (carer and family) and community level support and
- Makes use of digital tools to support intervention delivery

may be more acceptable to people living with MLTCs and frailty.

We have developed the PERSONAL-AGILITY intervention which:

- Uses a shared decision-making approach, supported by online tools and personalised videos, to allow participants to adapt the focus of the intervention in response to needs, preferences and circumstances
- Involves carers, so that they may support adherence but also improve their health
- Uses existing support from community groups via physical activity officers based in the local authority.

This study will explore the feasibility and acceptability of this intervention, directly responding to the following James Lind Alliance priorities for research on MLTCs in later life^74^ including:

- What is the most effective…and acceptable form of exercise?
- How can the…management of frailty be improved?
- How can carers…be supported to maintain their own…wellbeing?

# Research objectives and outcome measures

## Primary objectives

To determine the feasibility of a randomised controlled trial (RCT) investigating the effectiveness of the PERSONAL-AGILITY intervention for people living with MLTCs and frailty and their carers.

## Secondary objectives

- To determine the acceptability of the PERSONAL-AGILITY intervention for people living with MLTCs, their carers, and health and care professionals involved in the intervention, including key constructs relating to future implementation.
- To test the potential effectiveness of the PERSONAL-AGILITY intervention for people living with MLTCS, their carers, compared to usual care, on a range of secondary outcomes at 12 and 24 weeks.
- To evaluate the implementation of the PERSONAL-AGILITY intervention, its potential mechanisms of impact and the influence of context.

Addressing these objectives will enable us to refine the intervention and its programme theory as well as the trial procedures in preparation for a future grant application to definitively evaluate the effectiveness of the intervention.

## Outcome measures

### Primary

The following measures will be examined to quantitatively determine the feasibility of a future RCT of the PERSONAL-AGILITY intervention:

- Eligibility rates of participants screened by the research team
- Recruitment rate
- Retention rate
- Outcome measure completion rates

Qualitative data gathered from the process evaluation (see section 7.4) will also contextualise and enhance our understanding of these feasibility outcomes.

### Secondary

Data to assess the acceptability of intervention including:

- Rates of uptake and engagement with each component of the PERSONAL-AGILITY intervention
- Rates of adherence to the intervention
- The 24-hour Physical Behaviours Burden Questionnaire (Intervention group only)

Data to assess the potential effectiveness of the PERSONAL-AGILITY intervention in adults with MLTCs and frailty and their carers, compared to usual care, will be collected at 12 weeks and 24 weeks on the following secondary outcomes:

- Bioimpedance analysis
- Blood pressure
- Handgrip strength
- Short Physical Performance Battery
- The Berg Balance Scale
- 24-hour physical behaviour measurement (accelerometer and inclinometer):

o Physical activity (Stepping, light and moderate to vigorous physical activity)

o Sedentary behaviours (total duration and breaking up inactivity)

o Sleep behaviours (Quality and quantity)

- EQ-5D-5L
- Short Form 36
- The Late Life Function and Disability Instrument
- The Patient Outcome Scale
- Goal Attainment Scale,
- Zarit Burden Interview (carers only)

Data to evaluate the implementation of the PERSONAL-AGILITY intervention, its potential mechanisms of impact and the influence of context including:

- Implementation fidelity assessment
- The 9-item shared decision-making questionnaire (Intervention group only)
- Semi-structured interviews
- Implementation process data

# Trial design

This is a 24-week feasibility randomised controlled trial, with a mixed-methods process evaluation. The trial schematic on page 19 provides an overview of the trial design and core trial visits and process evaluation.

Fifty Individuals aged ≥18 years living with MLTCs and frailty will be enrolled. Participants will be approached via diverse means (including through primary care, secondary care and public advertisement/self-referral), before undergoing eligibility and consent procedures by telephone and/or in-person as required and preferred. Carers will also be invited to take part in the trial as part of a dyad.

Participants will be allocated in a 2:1 ratio in favour of the intervention group, to one of two groups:

- The PERSONAL-AGILITY intervention
- Ongoing USUAL care

Allocation will use stratification based upon frailty status. All participants will undertake three assessments:

- Visit 1 – Week 0 – Baseline Assessments
- Visit 2 – Week 12 – Intermediate Follow-Up Assessments
- Visit 3 – Week 24 – Final Follow-Up Assessments

These visits may be combined where participants take part as a dyad or may be conducted separately if participants prefer.

An embedded mixed methods process evaluation, combining quantitative data (see section 2.3.1) gathered within the trial with observational and qualitative interview data, will provide additional insights into the feasibility and acceptability of both the intervention and the study design. Interviews will be conducted separately to ensure free expression of views and opinions.

The process evaluation will assess the:

- Trials acceptability, including recruitment, procedures and outcomes
- Intervention
  - Implementation: fidelity, dose and reach
  - Potential mechanisms of impact
- Contextual influences.

# Trial setting

The trial will be centrally coordinated from the Leicester Diabetes Centre (University Hospitals of Leicester NHS Trust) and Diabetes Research Centre (UoL) which are jointly situated at the Leicester General Hospital. The study will also be supported by the infrastructure provided through the NIHR Leicester Biomedical Research Centre and the Research Delivery Network (RDN).

Participants will be recruited from diverse settings, including direct engagement with primary, secondary and community care services (including via NIHR RDN local to each research site), as well as through a diverse range of media, engagement, advertisement and word of mouth.

# Participant eligibility criteria

## Inclusion criteria

All participants must be

- Adults ≥18 years
- Mobile (able to walk 5m including with the use of any walking aids)
- Able and willing to provide informed consent
- Able and willing to use digital and online tools with support as part of the intervention. Access to digital devices and internet is not an inclusion criterion as these resources can be provided if required to support participation.

**People living with MLTCs and frailty:**

- Living with ≥2 long-term non-communicable conditions.
- Living with frailty, defined as a Clinical Frailty Scale (CFS) score of 4-7 (very mildly frail to severely frail)

Participants identified via primary care may be identified as frail using the Electronic Frailty Index (eFI) (scores of 0.13-0.36+) or via the adjusted clinical groups (ACG) risk stratification tool. These participants will complete CFS scoring to confirm their frailty status. Given the varied routes for recruitment used, some people may express interest in the study who do not have any of these scores confirmed. These potential participants should be screened for their frailty status using the CFS (see section 6.3).

The Johns Hopkins Adjusted Clinical Groups (ACG) system is a well-established, multi-morbidity-based algorithm providing risk adjustment and predictive modelling capabilities and is widely used with primary care ^75^. ACG integrates primary and secondary care data to summarise multi-morbidity by incorporating all ICD-coded and Read-coded diagnoses that are recorded ^75^.The CFS (see Appendix A) is a risk stratification tool with good predictive abilities correlates well with the Frailty Index ^76^.

**Carers:**

- Providing regular informal care to someone living with frailty and MLTCs as defined above for ≥3 months

Informal care includes (but is not limited to) emotional support, prompting with taking medications, getting prescriptions, managing, and organising appointments and care tasks, encouraging participation in social events and physical activity, helping with household tasks, or providing physical care.

People with MLTCs and frailty and their carers may be recruited and involved in the intervention in a dyad, but carer involvement is not required for people with MLTCs and frailty to participate.

## Exclusion criteria

For all participants:

- Unable to provide informed consent
- Unable to communicate in English
- Known contraindications to exercise (as defined by the American College of Sports Medicine)^77^ to include:
  - Unstable cardiac disease (Uncontrolled arrhythmias; unstable angina or heart attack within the last 3 months; persistent uncontrolled hypertension (systolic blood pressure >180 mm Hg or diastolic blood pressure >110 mm Hg); stroke or transient ischaemic attack within the last 3 months)
  - Active infection
  - Significant aortic aneurysm (more than 5.5 cm)
  - Any other condition in which the investigator feel exercise may be contraindicated.
- Current participation in competing clinical trial (as determined by study investigator of this trial).
- Significant cognitive impairment (Mini mental state examination score of less than 24)^78^ or unstable psychiatric disorder that limits active participation in the intervention.
- Serious illness or event with life-expectancy <1year, active malignancy (on chemotherapy/radiotherapy) or other significant illness which, in the opinion of a study clinician, precludes involvement.
- Self-reportedly already regularly engaging in at least 150 minutes of moderate-to-vigorous physical activity per week.

People with MLTCs and frailty:

- Not frail as defined by:
  - eFI:0-0.12 or
  - A CFS score of 1-3 (very fit to managing well).

Carers:

- Providing paid/professional care.

# Trial procedures

This section provides a clear and concise timeline of the trial visits, enrolment process, interventions, and assessments that participants will undertake. These are also summarised in the schedule of procedures outlined below.

## Schedule of procedures

Table 1. Schedule of procedures for the PERSONAL-AGILIY study.

|  | Screening | Consent | Baseline | Week 12 | Week 24 |
| --- | --- | --- | --- | --- | --- |
| Screening* | A |  |  |  |  |
| Consent |  | A |  |  |  |
| Demographic and Medical History# |  |  | A |  |  |
| Body Composition |  |  | A | A | A |
| Blood Pressure |  |  | A | A | A |
| Handgrip Strength |  |  | A | A | A |
| Short Physical Performance Battery (SPPB) |  |  | A | A | A |
| Berg Balance Scale |  |  | A | A | A |
| Physical Activity** |  |  | A | A | A |
| Sedentary Behaviour** |  |  | A | A | A |
| Sleep** |  |  | A | A | A |
| EuroQol-5 Dimensions |  |  | A | A | A |
| Short Form-36 |  |  | A | A | A |
| Late Life Function and Disability Instrument |  |  | A | A | A |
| Patient Outcome Scale |  |  | A | A | A |
| Goal Attainment Scale |  |  | A | A | A |
| Zarit Burden Interview |  |  | C | C | C |
| The 9-item Shared Decision-Making Questionnaire (intervention group only) |  |  |  | A | A |
| The 24-hour Physical Behaviours Burden Questionnaire (intervention group only) |  |  |  | A | A |
| Randomisation*** |  |  | A |  |  |
| Adverse Event Reporting |  |  | A | A | A |
| Interviews |  |  |  |  | A |

A = All participants; P = People with MLTCs and frailty only;C = Carers only.* Screening includes checks for confirmation of eligibility, as described in section 5. #Demographic and medical history includes: sex, date of birth, ethnicity, employment, education, marital status, socio-economic status, and a detailed past medical history including previous or current diseases and surgical interventions will be recorded by the study clinician on the CRF. **Physical activity, sedentary behaviour and sleep will be measured by an accelerometer. Measures include: Device measured steps, MVPA, sedentary behaviour, sleep quality and sleep duration.^***^Randomisation will occur on completion of baseline measures.

## Recruitment

All regulatory approvals, confirmation of capacity and capability and sponsor greenlight will be in place before participants are identified and approached.

### Participant identification

The recruitment phase will commence as soon as all necessary approvals have been received. Potential participants may be identified and/or contacted in the following ways.

### Recruitment from primary and secondary care

Researchers will work closely alongside their local RDN to support recruitment from primary and secondary care, including Participant Identification Centre (PIC) sites. The RDN will support recruitment via their study support service advertising the study and gathering expressions of interest to act as a PIC from sites within LLR. Potential participants will be identified primarily through database searches on clinical systems/electronic health records (I.e., SystmOne). The database search will be completed by people with legitimate access and initially screen for eligible participants based on the criteria outlined in section 5. A study invitation letter with accompanying reply slip/, along with a brief participant information leaflet including a QR code with link to the study website (see section 6.2) will be provided to potentially eligible participants. If no response has been received, a remailing will be conducted. If willing, GP practices will add notes or reminders to medical records within Electronic Health Records to aid recruitment. The study teams will work with the RDN and GP practices to add notes and/or set-up reminders on eligible medical records for a GP or other health care provider to inform them about the trial during routine appointments. In addition, where willing, individual clinicians, GPs and other health care professionals (e.g. practice and clinic staff, social prescribers) may engage in opportunist identification of potentially eligible participants with the provision of study materials and the participant information leaflet.

Recruitment through secondary care will include identification of potentially eligible participants via clinical databases, with subsequent contact as outlined for primary care databases above. The study team will also coordinate with local secondary care services to attend relevant clinics and discuss the study with potential participants who have expressed interest after being told about the study by their health and care providers. Health and care professionals with legitimate access will screen their clinic lists for potentially eligible participants and approach potentially interested and eligible people.

After seeking consent to do so, contact details of interested people identified by health and care practitioners from either primary or secondary care will be shared with the research team, who will then contact the potential participants and share study materials. Whether approached in primary or secondary care, non-clinical members of the research team, or clinical members not involved in the person’s usual care, will not identify participants or access medical records before consent has been obtained. Researchers may however visit clinical sites to promote the study to clinical teams.

### Recruitment through participant and volunteer database

If applicable, participant and volunteer databases within the Leicester Diabetes Centre containing individuals who have consented to be informed of and invited to future research studies may be used. These may be participants that have completed studies previously or volunteers that have not previously been involved in studies but have expressed interest to be considered for future studies.

### Recruitment through community engagement

Participants may be recruited through locally organised relevant community health events. These may consist of having a stand with all the study information and/or presenting the study at these events. Awareness campaigns may also be used as a route to promote the study.

### Recruitment through other methods

The study will be promoted and advertised via several formats including, but not limited to, social media and press releases.  Participant case studies (with media consent in line with UHL Trust policies), which will be used to promote the trial on social media, websites and press releases. Study information, including a brief leaflet about the study, will be distributed by email to various mailing lists held by the University of Leicester NHS Trust and other relevant partner organisations, including but not limited to, local registries, newsletters, Trust members, public and patient involvement/engagement (PPI/E) groups, and Trust staff, local intranets and internal mailing lists.

A range of media may be used to promote the study, including (but not limited to) animations, videos, and QR codes enabling people to access more information. The study team will also distribute posters and information to publicise the study across both clinical (e.g., GP practices, local hospitals, pharmacies) and non-clinical environments (e.g., supermarkets, libraries and community centres), All methods of advertisement will contain the study acronym and logo, a description about the study and contact details of the research team.

### Recruitment procedures

Potential participants identified via primary and secondary care, and those who express interest after seeing local advertising, volunteer database and community engagement will either:

- Be sent a postal or email invitation pack, which will include a letter, participant information sheet, contact details for the research team, and a (where relevant) a reply slip with a pre-paid envelope providing consent for the research site to contact them.

Or

- Scan a QR code which will take them to further information on the study online. A study website containing information about the study will enable potential participants to contact the research team.

Remailing and further follow-up, via email, SMS messaging, phone and other appropriate methods, of non-responders will be considered according to how well recruitment targets are being met.  All potential participants who reply by post or email will be contacted to provide further explanation of the study prior to consent.

### Reimbursement

Participants travel and parking expenses for all visits associated with the study (including those for the interviews undertaken as part of the process evaluation) can be reimbursed.  Participants who are interviewed will be offered a £20 voucher in recognition of their time, and for those interviews which are conducted online, an ‘uplift’ of £5 will be offered. Caring costs will be covered for carers who are interviewed separately in line with current NIHR guidance (up to £15 per hour)^79^.

### Consent

Further information on consent procedures for the interviews can be found in Section 7.5. Participant information sheets, consent forms and any amendments will follow GCP, local regulatory requirements and legal requirements and will have been approved by Research Ethics Committee (REC), Health Research Authority (HRA), Study Sponsor and the local trust R&I department prior to implementation*.*

To allow a wider range of participants to take part should they wish and to ensure that an undue burden is not created, an approach which allows for informed consent to be taken both in-person and remotely via telephone or secure video conferencing (e.g., Zoom or Microsoft Teams) will be used.

Written informed consent will be received after individual discussion between the participant and a member of the research team, with the participant having had sufficient time (at least 24-hours) to consider the participant information sheet, confer with other parties (e.g., family members), and ask any questions related to the study. Written informed consent will be obtained by either by:

- Means of participant dated signature and dated signature of the person who completed consent procedures (for in-person consent)

or

- The researcher will initial the boxes in a bespoke consent form and sign on behalf of the participant (for telephone or online consent).

The person obtaining informed consent will be a suitably trained and competent person who, in the opinion of the Principal Investigator (PI) at each site, will be able to give a full and unbiased explanation of the study (including benefits and risk) to the potential participant. As part of the process, they will make an informed judgement on the person’s capacity to provide informed consent, by checking the participant understands:

- The purpose and nature of the research
- What the research involves, including its potential benefits risks and burdens
- The alternatives to taking part

and that they can:

- Retain the information long enough to make an effective decision
- Make a free choice
- Make this decision at the time it needs to be made.

The person obtaining consent will also have been named in the delegation log of staff as undertaking this duty and approved as study personnel by the relevant governance procedures. Written and audio versions of the participant information sheet and informed consent forms will be presented to the participants detailing no less than: the exact nature of the study; the implications and constraints of the protocol; the known potential risks involved in taking part. It will be clearly stated that the participant is free to withdraw from the study at any time for any reason without prejudice to future care, and with no obligation to give the reason for withdrawal. Participants will also be informed briefly about the process evaluation and be required to consent to being recorded as part of this if required. They will also be required to consent to be contacted about the interviews, although they will not be obliged to take part. A separate consent process will be undertaken for the interview stage (see section 6).

Each participant will be provided with a contact point where they may obtain further information about the trial, a copy of the consent form and participant information sheet. A copy will be placed in their hospital medical records, where possible, and the original copy held in the site master file. A screening log will be designed to identify trends and capture numbers of people screened, eligible, approached, randomised, and numbers accepting their randomised allocation.

The PI will retain overall responsibility for the conduct of the research, including the receipt of informed consent of participants. Where a participant is required to re-consent or new information is required to be provided to a participant it will be the responsibility of the PI to ensure this is done in a timely manner.

## Screening

Screening for eligibility will be performed by an appropriately trained delegated person and will involve telephone, online or in-person screening (according to participant preference) to confirm aspects of the inclusion and exclusion criteria ahead of consent. In-person assessment will take place at the research site, in the participant’s home, or at community locations to increase inclusivity.  Any adverse findings that come to light throughout pre-screening (or at follow-up assessments) will be reported to the participant’s GP. Anonymised information on participants who do not progress beyond each stage of the recruitment and randomisation process will be recorded for CONSORT reporting, including:

- Age
- Sex
- Ethnicity
- The reason they are not eligible for trial participation, or if they are eligible but declined.

A standardised bespoke screening form will be used to check mobility and physical activity exclusion criteria. Potential participants who have been identified as frail via an eFI score or flagged by the adjusted clinical groups (ACG) risk stratification tool at the eligibility phase, or do not have any identified frailty scoring at this stage, will have their frailty status confirmed via CFS score. Cognitive impairment will be screened for at this stage using the MMSE.

## Carer consent

Once eligibility has been confirmed, participants will be asked if they wish to invite a carer to participate with them. Interested carers will be provided with their own brief information leaflet and undertake the consent process outlined within section 6.2.8. Some participants may wish to have a carer present with them during their consent and screening procedures. In these circumstances it may be appropriate to discuss the study with both parties concurrently, and to undertake consent and screening together.

## Baseline data

Prior to randomisation, but after consent, participants will be asked to complete several questionnaires and measures, detailed in section 7. All participants will be asked for consent to access their medical records.

Baseline assessments may be undertaken in any order; although to facilitate complete data collection a preferred assessment order will be established and outlined through the visit case report form. In addition to the measures outlined in section 7, demographics will be collected via a case report form (CRF) completion. For all participants these will include:

- Date of birth (subsequently calculating age)
- Sex
- Gender
- Ethnicity
- Frailty status (CFS) if not already established during screening
- A detailed past medical history including previous or current diseases (subsequently calculating number of long-term conditions)
- Medications

Information on socioeconomic status data collection, informed by relevant NIHR guidance ^80, 81, 82^ will include:

- Living arrangements
- Housing status
- Employment status
- Receipt of benefits
- Level of education attainment
- marital status
- Participant postcode to calculate the index of multiple deprivation (IMD) score

The 3-Item Digital Health Care Literacy Scale will be used to determine participants confidence, familiarity with digital tools and devices^83^. The scale is reliable and validated for use in English speaking populations^83^. This will be undertaken as a one-off measure at baseline, allowing the research team to determine the level of support the participant may need with the digital tools included within the intervention. Participants in the intervention group with low scores (≤ 6) will be supported to access and use the tools as required.

For carers only the following additional information will be collected:

- Relationship to the care recipient
- Length of time in current caring role
- Number of hours of care provided per week
- Caring tasks performed

The baseline visit will occur preferably within 28 days of screening. Baseline assessments will be conducted on a single day as standard but may be conducted across multiple visits where required (e.g., due to participant preference, staff or equipment availability), provided they are completed within a 14-day window.

## Randomisation

Randomisation will occur after baseline assessments. Eligible participants who are living with MLTCs and frailty (and their carer if included) will be randomised in a 2:1 ratio to one of two arms:

- Intervention group: the PERSONAL-AGILITY intervention

OR

- Control group: usual care

The increased ratio of participants within the intervention group will give increased opportunity and exposure to the delivery of the intervention, which will be beneficial to refining and improving it for a future definitive trial.

Stratification will be based on one factor, frailty status, creating two strata (very mild and mild/ moderate and severe). Randomisation and treatment allocation will be performed using a third-party service using variable block sizes of 3 and 6.

### Method of implementing the randomisation/allocation sequence

Allocation will be performed using a validated web-based system (Sealed Envelope Ltd). A letter will be sent to the participant’s GP, notifying them of their patient’s participation in the study and confirming randomisation assignment.

Carers will be randomised as a dyad with the person with MLTCs and frailty. Each participant will be given a unique participant identification (ID) number at randomisation. For people undertaking the intervention with a carer, these ID numbers will be linked.  Participant ID numbers will be used to identify the individual participant throughout the study and will not be re-assigned to any other participant. Due to the nature of the intervention, blinding of the participants and the study team to allocation is not possible. The allocation will be documented within the trial master file and, where possible, participant medical notes.

## Blinding

It is not possible to blind participants and intervention providers to allocation, due to the nature of the intervention. Furthermore, outcome assessors will not be blinded as this feasibility study does not aim to evaluate intervention effectiveness. Scripts for helping participants who need support to complete PROMs will be developed to minimise response bias.

## Trial intervention

PERSONAL-AGILITY is a complex intervention that uses a range of approaches tailored to individual needs. This study will have the option to use different technology that can be used at home and in the community to help adapt to individual needs and preferences.

### Technology

The PERSONAL AGILITY intervention will use novel technology where it reduces burden and adds value for the individual, including bespoke interactive digital platforms and wearable technology (e.g., physical activity monitors, such as Fitbits or apple watches), to support:

- Collection and review of clinical information; by both participants and health and care professional (HCPs) involved in the delivery of PERSONAL AGILITY
- Intervention delivery; some areas may use existing services
- Ongoing monitoring/support; including provision of feedback, reminders/alerts, virtual consultations and peer support.

Importantly, the use of technology will complement, not replace, face-to-face contact. Many individuals may prefer the personal nature of face-to-face discussions with their HCPs and personal contact may facilitate conversations that would not be had via purely digital means. A solely digital approach may not be inclusive for all individuals, so alternative non-digital means of engagement will be available where appropriate/preferred. PERSONAL AGILITY technology will involve the following key platforms, which we have specifically refined to meet the needs of people living with MLTCs and frailty, and those who care for them:

#### MyHealthMapp

MyHealthMapp is a tool designed to facilitate a conversation around the 24-hour physical behaviours between people living with long-term conditions and their health care professional. MyHealthMapp is an online tool (web application) designed to track/log health, wellbeing and 24-hour physical behaviour data (trend of data, changes, patterns of activity) automatically via a physical activity device and/or entered manually. MyHealthMapp compares the data to personalised population norm values and generates a RAG (red, amber, green) rating to help users and their HCPs understand and interpret their results. MyHealthMapp also hosts short information videos for all measurements on the platform to help people understand what the physical activity and health variables mean. MyHealthMapp generates personalised targets for certain variables, i.e., step targets, based on participants previous results. Data stored on MyHealthMapp includes:

- daily stepping
- fitness
- non-stepping activity
- physical function
- strength
- sitting and sleep.
- glucose levels
- body weight
- wellbeing and holistic health.

#### Steps4Health

Steps4Health is a physical activity web application targeting 24-hour physical behaviours for people wanting to become active or living with a long-term condition. Steps4Health provides a personalised physical activity programme and ongoing support to help improve users’ health by becoming more active (focused on stepping, sweating [moderate to vigorous intensity physical activity] and strengthening) whilst also promoting improved sleep and reduce sitting timeSteps4Health includes:

- A personalised physical activity programme tailored to the user’s fitness and mobility level
- A walking programme to help the user increase their steps gradually for health benefits
- Online exercise sessions catered to all abilities, from seated workouts to full-body exercises. These sessions are available on demand for use at a time that suits the user
- Daily activity trackers including walking, sitting, workouts, and sleep
- Daily activity goals and notifications
- Direct access to the physical activity experts at the Leicester Diabetes Centre
- Competitions, challenges, and award notifications
- Interactive content about getting and staying active
- A chat forum offering vital peer support

#### Personalised videos

Videos, populated with data personalised to each participant and clinically approved health information, will be used to provide participants with a short summary of their current health and physical activity data, with a brief description of what the data means, before their intervention appointments. These videos will be used to help people:

- To break down complex terminology and enable them to understand their own results.
- Re-watch/ pause the video to help them process the information or write anything down.
- Show the video to others (i.e. family and/or carers)^84, 85, 86, 87^.
- Consider what they would like to speak about and any questions they may have ^84, 85, 86, 87^.

The aim of personalised videos are to improve the quality and experience of clinical appointments by generating informed discussions and allowing more time for participant questions and planning of goals/next steps ^84, 85, 86, 87^. Together with MyHealthMapp, personalised videos will facilitate shared decision making (see section 6.8) between the person and their HCP and enable them to be more active in their own care.

## Delivery of care

The PERSONAL-AGILITY intervention will be delivered by the research team and HCPs trained to deliver the PERSONAL-AGILITY intervention, using a range of delivery methods (in person, phone, online). The focus of the intervention (improvement of sleep, physical activity and reduction of sedentary behaviour) will be guided by a holistic assessment, facilitated by MyHealthMapp, using a shared decision-making (SDM) approach.

Shared decision making is a collaborative process in which the person and health care provider work together to make healthcare decisions that are best for their needs and circumstances^88^. Each decision will take into account the HCP knowledge/ experience and evidence around the treatment options as well as the participant’s preferences and values^88^. The SDM intervention will follow the 5 steps to SDM from the “SHARED approach” as outlined below ^88^:

- Seek the participant’s participation
- Help the participant explore and compare treatment options
- Assess the participant’s values and preferences
- Reach a decision with the participant
- Evaluate the participants decision

To help facilitate the SDM process the HCP will create a safe environment to support the conversation and utilise tools to facilitate the conversation (e.g., personalised goal setting) and may include the use of personal decision-aids and other documents designed to support participant engagement.

Linking participants with community groups within their locality will also be a feature of the intervention (this may not be available to all participants), and where possible, they will be supported to access activities groups and services in their community that either directly or indirectly reduce their sedentary behaviour and increase physical activity. ‘Direct’ refers to groups which focus on increasing physical activity e.g., a seated exercise class. ‘Indirect’ refers to groups which have a non-physical activity focus but increase participation and may increase physical activity through the functional activity needed to prepare for going to the group, travelling to the group, and movement during the group.

## Usual care

Ongoing standard care (for participants in the control group) will continue to be delivered by participants’ usual NHS service providers in primary and secondary care and will not be altered or standardised as part of the trial. Usual care will reflect routine practice where participants may be referred into single disease rehabilitation. Attendance at any rehabilitation programme (or similar) will be recorded. Participants in the control group will receive generic information on the 24-hour physical behaviours.

# Trial assessments

## Trial visit schedule

Trial procedures and study outcomes related to the trial will be distinct from measures undertaken as part of the PERSONAL-AGILITY intervention. Trial assessments measured at research visits will be used to examine the feasibility and acceptability of the PERSONAL-AGILITY intervention on the outcomes outlined in sections 7.1.1 and 7.1.2.

Visits will occur at weeks 0, 12 and 24. As per baseline assessments, it is intended that all assessments at each follow-up time point will be completed on a single day but may occur across multiple visits provided they are completed with a maximum 14-day window. At each time point, willingness to continue will be confirmed and documented on the CRF, and participants will be asked to undertake all the measures outlined. Relevant assessments will be performed at each visit as described. There will also be an option for home and community-based testing in line with PPI suggestions to help make the intervention more inclusive where possible.

An unscheduled visit can be used when a participant misses their appointment, is outside of their visit window due to unforeseen circumstances, or repeat assessments are required, but this will be logged accordingly as being outside of intended trial timelines and/or additional assessments (e.g., protocol deviation).

### Feasibility of trial methodology

The following measures will be examined to determine the feasibility of a future RCT of the PERSONAL-AGILITY intervention:

**Eligibility rate**

- Eligibility rates
- Reasons for ineligibility

**Recruitment rate**

- Recruitment rates
- Recruitment rates amongst people from minority ethnic and socioeconomically deprived backgrounds
- Basic characteristics (age, sex, ethnicity, frailty status if known) of those who decline
- Reasons for declining

**Retention**

- Rates of attrition at each stage of the study (screening, consent, randomisation, baseline, follow up (12 and 24 weeks)
- Reasons for withdrawal

**Trial outcome measures**

- Outcome measure completion rates for all secondary measures

Time taken to complete outcome measures

- Reasons for missing data (if known)

**Other feasibility measures**

The feasibility of having a co-primary outcome specifically for carers in the definitive trial will be explored using data based on rates of recruitment of carers, carer completion rates and their feedback gathered as part of the process evaluation/ assessment of acceptability.

Quantitative feasibility data will be gathered from a range of sources, including screening and recruitment logs and CRFs.

### Acceptability of trial methodology and intervention

The acceptability of the trial and intervention will be measured both quantitatively and qualitatively. This section outlines the components of acceptability that will arise directly from trial and intervention data. Section 7.3 provides further information about qualitative acceptability data. This will be complemented and expanded by additional data from a mixed methods process evaluation data, which is discussed in detail in section 7.3.

Quantitative data relating to acceptability includes:

- Rates of uptake and engagement with each component of the PERSONAL-AGILITY intervention, including but not limited to:
  - MyHealthMapp
  - Steps4Health
  - Linking to community services and groups
  - Attendance at community services and groups

These data will be gathered from reports from the digital tools, trial documents and a data capture form which will be completed by the physical activity officer (A draft example is provided in appendix B).

- Rates of adherence to the intervention

Intervention adherence will be captured using a bespoke diary, including:

- Frequency, duration, intensity, time, type and consistency of physical activity
- Bouts of non-sedentary time
- Reasons for non-adherence

The above two measures will provide an indication of participants engagement with the intervention received ^89^.

- The 24-hour Physical Behaviour Burden Questionnaire

At weeks 12 and 24, the 24-hour physical behaviour burden questionnaire will be used to assess burden in participants in the intervention group, as a PROM relating to intervention acceptability. This is a modified from the exercise therapy burden questionnaire to establish burden across all of the 24-hour physical behaviours. The original exercise therapy burden questionnaire is a 10-item instrument that measures intervention burden in participants with long term conditions.^90^ The measure has been modified in the absence of any other appropriate measure of burden.

## Secondary outcomes

The selection of a primary outcome measures for an intervention which:

- Allows for individualisation amongst participants in the intervention group, in line with a SDM approach
- Allows for the focus of the intervention to change within a single individual participant over time, also in line with a SDM approach
- Allows for the inclusion of a carer as an equal participant within the intervention

May present a challenge for the selection of an appropriate, sensitive and responsive primary outcome measure which is also meaningful to this population. We will gather data on a range of objective and patient reported outcomes (PROMS) to establish which measure(s) may be the most appropriate primary measure in a future definitive trial.

The following secondary outcomes will be gathered to inform the selection of a primary outcome measure/ co-primary outcomes for carers and people with MLTCs and frailty. This determination will be made alongside feasibility data relating to outcome measure completion rates. Objective tests will all have standardised standard operating procedures (SOPs) to ensure consistency of measurement. All participant groups (carers and people with MLTCs and frailty) will do all the tests, except where a specific population is identified. PROMS may be completed with the support of researchers (using standardised scripts) or may be completed by the participants at the study appointments, or taken home and returned at a later date.

### Physiological measures

- **Body composition**

At baseline, weeks 12 and 24, body weight will be collected (to the nearest 0.1 kg respectively). BMI will be calculated based on body weight and height. Bioelectrical Impedance Analysis (BIA) will be used to estimate body composition, including body fat percentage and lean body mass and fat mass.

- **Blood pressure (systolic and diastolic)**

At baseline, weeks 12 and 24, arterial blood pressure will be measured in the seated position using an automated sphygmomanometer after participants have been resting for ~5min. Three measurements will be obtained and the average of the last two measurements will be used.

- **Handgrip strength**

At baseline, weeks 12 and 24, handgrip strength will be measured using a handheld dynamometer three times on each side, with the elbow flexed at a right angle and the forearm in neutral position ^92^. The maximum of the readings generated is taken as the maximum grip strength.

### Physical function

- **Short Physical Performance Battery**

At baseline, weeks 12 and 24, the Short Physical Performance Battery (SPPB) will be used to objectively measure physical function. The SPPB measures balance, gait speed and ability to stand from a chair^93^. It comprises three tests outlined below, each scored out of 4 (for a maximum total score of 12) in accordance with available guidance:

- Chair sit-to-stand: The participant will start from a seated position on a hard, upright chair (such as a dining chair) of standardised height, with the feet flat on the floor and the knees bent at 90°. For the test, the time taken for the participant to stand up fully and then return to sitting 5 times without using the hands is measured.
- Standing balance: Static standing balance is tested in three progressive positions. If the participant can maintain the specified position for at least 10 seconds, then the position is progressed to the next stage:
  - Feet together
  - Semi-tandem
  - Tandem
- Gait speed: Gait speed is calculated from the time taken for the participant to walk 4 metres at their usual speed on a level course. It is measured twice with a short break in between, and the best score used.
- **Berg Balance Scale**

At baseline, weeks 12 and 24, the berg balance scale will be used to assess the participants balance^94^. The berg balance scale includes 14 tasks that are scored on a 5 point scale, with 0 as the lowest level of function and 4 as the highest^94^, with the total score being out of 56. The berg balance scale is a valid and reliable to assess balance^95^.

### Accelerometer and inclinometer measured physical behaviours

At baseline, weeks 12 and 24, we will aim to assess physical activity, sedentary behaviour and sleep parameters objectively using accelerometery worn for approximately 9 full days (24-hours per day) to capture 3-4 days with >10 hours of valid wear time^96^. Two devices (one wrist-worn, GeneActiv (accelerometer), on the non-dominant side, and the other, activPAL (inclinometer) placed on the thigh) will be worn. The GeneActiv will capture triaxial acceleration data and data will be processed using an open-source R programme (GGIR) according to SOPs developed for the NIHR Leicester Biomedical Research Centre^97^. Postural allocation (sitting and standing) and walking will be quantified using an activPAL physical activity monitor (PAL Technologies, Glasgow, Scotland). The activPAL is a single-unit monitor based on a triaxial accelerometer that is worn midline on the anterior aspect of the thigh and attached directly onto the skin using medical dressing. The monitor produces a signal related to thigh inclination and has been shown to be a valid and reliable measurement tool for determining posture during activities of daily living in a healthy population. Participants will be given a daily log sheet to record their sleep times each day and any times that they may have removed the monitor, to facilitate future analyses.

### Patient reported outcome measures (PROMS)

- **EuroQol-5 Dimensions (EQ-5D-5L)**

At baseline, weeks 12 and 24, self-reported quality of life will be assessed using the EQ-5D-5L questionnaire. The EQ-5D-5L is used as a standardised measure of self-reported health status and quality of life and is considered to have a good discriminatory power and validity. The EQ-5D-5L will be used to determine health state descriptions for five components combined with preference-weighted health-related quality of life index scores (as approved by NICE) to generate Quality Adjusted Life Year (QALY) profiles for the cost-effectiveness analysis^98^.

- **Short Form-36**

The Short Form 36 Health Survey Questionnaire (SF-36) is 36 item measure of health-related quality of life across multiple domains assessing functional status and wellbeing^99^. It has shown to be an acceptable, valid and reliable measure of measuring health-related quality of life. The questionnaire will be scored in accordance with available guidance.

- **Late Life Function and Disability Instrument (Late-life FDI)**

At baseline, weeks 12 and 24, the late-life function and disability instrument (late-life FDI) will be used to assess changes in function and disability^100^. The late-life FDI is split into disability that looks at socially defined tasks and functions that involves activities of daily living^101^. The disability section is 16 items and each question should be rated on a scale of 5-1 for a) frequency (i.e. “how often…”) and b) limitation (i.e. “to what extent do you feel limited in…”)^101^. The function section is 32 items and each question is rated on a scale of 5-1 ^101^.

- **Patient Outcome Scale (POS)**

At baseline, weeks 12 and 24, the Patient Outcome Scale (POS) will be used to measure the physical symptoms, psychology, emotional, spiritual information and support needed for people with long term conditions^102^. The POS is a widely used, multidimensional, 11 item assessment of quality of life for people with long term conditions^102^. The answers are on a 5 point Likert scale (aside from question 9 which has 3 points and an open question around main problems experienced by participants)^102^. The total score is out of 40, with 0 indicating a better quality of life and 40 indicating a lower quality of life^102^.

- **Goal Attainment Scale (GAS)**

At baseline, weeks 12 and 24, the goal attainment scale (GAS) will be completed by participants.  GAS is a way to measure to what extent participants' goals have been achieved during the intervention on a standardised 5-point scale and converted to a T-score to provide a single figure that reflects the overall extent of achievement and allows for analysis.^103^. The GAS is widely used in healthcare, including areas such as rehabilitation, physical therapy and care in an older populations ^103^.

- **Zarit Burden Interview (carers only)**

At baseline, weeks 12 and 24, the Zarit Burden interview will be used for carers only. The Zarit burden interview is a 22 item questionnaire used to assess carers perceived burden of providing care using a 5 point Likert scale  ^104^. Scores are added up (out of 88), with higher scores suggesting greater burden  ^104^. The Zarit burden interview is one of the most used measures of burden and is a valid and reliable instrument to measure carer burden ^104^.

## Mixed methods process evaluation

An embedded mixed methods process evaluation, combining relevant data gathered from multiple sources will be conducted according to the NIHR/ Medical Research Council guidelines^89, 105.^ Normalisation Process Theory (NPT) will provide an additional sensitising framework for data collection and analyses of the interviews^106, 107^. Further information about NPT is provided in section 7.7.

The process evaluation will focus on the following priority areas which have been identified by the research team as areas of greatest importance ^89^:

**1.Intervention:**

**Implementation**

Assessments of implementation will include establishing:

- Fidelity
  - Whether the key functions of the intervention were delivered as intended according to the fidelity checklist
  - The quality of intervention delivery according to the fidelity checklist and questionnaire data (see section 7.3.2)
- Dose
  - The number of reviews attended
  - The number of physical activity officer sessions attended (where applicable)
  - The number of community groups sessions attended (where applicable)
- Reach
  - Whether the intended audience encounters the intervention, and how

**Mechanisms of impact**

Potential mechanisms of intervention effectiveness and of participant engagement with the intervention

**Context**

How contextual factors influenced intervention adoption and impact, including any unintended consequences.

**Acceptability**

**Including:**

- - exploration of the influence of interaction between participants who took part as a ‘dyad’
  - The inclusion of physical activity officer support and engagement with community groups, where this has been possible.
  - barriers and facilitators to engagement

**2 Trial:**

The acceptability of key trial procedures, outcome measures and exploration of any unintended harms and consequences

These methods used to address these areas (including quantitative intervention process data described earlier in section 7.1.1), and with which stakeholder groups are outlined in Table 2.

Table 2. Methods used within the process evaluation.

| **COMPONENT** | **IMPLEMENTATION** | | | **MECHANISM OF IMPACT** | **CONTEXT** | | **INTERVENTION ACCEPTABILITY** | | **TRIAL ACCEPTABILITY** |
| --- | --- | --- | --- | --- | --- | --- | --- | --- | --- |
| **PARTICIPANTS** |  | HCPs* | Participants | Participants♯ | HCPs* | Participants♯ | HCPs* | Participants♯ | Participants♯ |
| **METHODS AND DATA COLLECTED** | Fidelity | - Audio recording of sessions and Fidelity checklist - SDM questionnaire data from the intervention group | Engagement/ adherence process data^+^ | Interview | - Interview - Site contextual information | Interview | Interview | - Interview - Engagement/ adherence data^+^ - 24 hour physical behaviours questionnaire | Interview |
|  | Dose | Process data on number of:   - reviews - physical activity officer sessions - community groups   attended | Engagement/ adherence process data^+^ | Secondary outcome data** |  |  |  |  |  |
|  | Reach | Process data on:   - characteristics of participants receiving the intervention - completers and non-completers - those with high engagement vs low engagement^+^ | |  |  |  |  |  |  |

Abbreviations: HCP, health and care professionals; SDM-Q-9, 9-Item Shared Decision-Making Questionnaire. *’HCP’ refers to those providers who are involved in the intervention delivery, including physical activity officers and community providers. ** secondary outcome data is detailed in section 7.2. ^+^ Engagement and adherence data is detailed in section 7.1.2. ♯ ‘Participants’ refers to people living with MLTC and frailty and carers with the intervention group (see section 7.3.1) and includes those who have withdrawn voluntarily.

### Participant eligibility criteria for the mixed-methods process evaluation

Participants and their carers who are enrolled in the trial and randomised to the intervention arm will be eligible to participate in the process evaluation. Those who voluntarily withdrew, but not those who were withdrawn for reasons of ill-health, illness, injury, progression of disease etc, from the intervention will remain eligible for interview.

Health and care professionals (defined as physical activity officers and other link or community workers who are involved in the delivery of the physical activity officer components of the PERSONAL-AGILITY intervention, or the local community groups that participants choose to attend as part of the intervention) will also be eligible provided, they are:

- Clinical and non-clinical staff delivering the PERSONAL-AGILITY study or community-based sessions the participant has accessed as part of the intervention.
- Aged ≥ 18 years
- Able to provide written informed consent

### Data collection

In addition to the quantitative acceptability data outlined in section 7.1.3, quantitative process data will include assessment of intervention fidelity. Due to the feasibility nature of this study, the intervention (apart from the physical activity officer sessions which will be delivered by the physical activity officers and community providers) will be delivered by the research team. Given that the intervention:

- Is complex and targets multiple behaviours (sleep, physical activity and sedentary behaviour) which are selected by the participants in line with a shared decision-making approach
- Can include both carers and people with MLTCs in a ‘dyad’

an assessment of intervention fidelity for those sessions provided by the research team will still provide useful data that will inform the future implementation of the intervention within a definitive trial and to refine a training package for intervention providers within this definitive trial.

Informed consent to audio record PERSONAL-AGILITY sessions will be sought from all participants. Audio-recording all sessions is the current gold standard for intervention fidelity assessment^108^. The fidelity assessment will be completed using audio recorded sessions and a fidelity checklist and coding manual developed by the study team. and will be undertaken by trained observers. This checklist and manual will be developed *a priori* by two researchers to ensure it accurately reflects the PERSONAL-AGILITY intervention, but will include the following areas:

- Duration and focus of the intervention
- Intervention providers’ behaviours consistent with a shared decision-making approach
- Behaviour change techniques used as intended
- Key messages, or content of the intervention is delivered as intended
- Digital tools and other resources and materials used as intended
- Adaptations made to the intervention

Where possible, two people will undertake the fidelity assessments and inter-rater reliability assessed. Once agreement is established, intervention fidelity assessments may be carried out by one researcher.

All sessions will be audio-recorded with audio-recordings from initial and follow-up assessments, physical activity officer sessions (where applicable) and follow-up phone calls included. Around 10% of the programme delivered will be analysed, which is consistent with recommendations within the literature ^108^.

Sessions analysed will purposively include a mixture of early (after the first six months of recruitment) mid (midway through recruitment) and late sessions (within the last six months of the study) as per the qualitative interviews (see section 7.7), from across the duration of the study to account for intervention provider practice effects. This will also account for the fact that emerging results from the process evaluation will be used to iteratively improve delivery of the intervention and the study, so fidelity may vary over time for this reason^89^.

In addition to the fidelity assessments, at weeks 12 and 24, the 9-item shared decision-making questionnaire (SDM-Q-9) will be completed by participants within the intervention group. The questionnaire is used to assess the HCP and participant behaviours during decision making using a 6-point Likert scale ranging from “completely disagree” to “completely agree” in response to statements such as: “My doctor explained the advantages and disadvantages of the treatment options”^109^. The 9-item shared decision making questionnaire has good reliability, validity and feasibility ^109^.

Information on dose and reach of the intervention will be gathered from intervention records and participant characteristics from the trial. Local authority profile questionnaires, informed by the Context and Implementation of Complex Interventions (CICI) framework, will be collected to provide contextual information for each area and situate the analysis and interpretation of findings; these will contribute to better understanding the context in which the intervention is delivered. An initial outline for the profile questionnaire is included in Appendix C.

## Qualitative interview data collection

Data on mechanisms of impact, context, intervention and trial acceptability will be gathered via semi-structured interview. These data will be supplemented with other data from the trial as outlined within Table 2.

### Sample size

For the qualitative interviews we anticipate sampling:

- ~ *n*=15 People living with MLTCs and frailty
- ~*n*=15 Carers
- ~*n*=15 HCPs (physical activity officers and other link workers or community providers involved in the intervention)

As we are examining variation in data, data saturation is not an appropriate criterion for judging the point at which data collection might cease. Information power will be assessed throughout, and the final sample size determined by its ability to provide rich data which addresses the stated aims^110^.

### Sampling technique

Maximum variation sampling of participants will primarily be guided by:

- Level of frailty for people living with MLTCs and frailty (the latter dichotomised as ≥2 LTCs or ≥3 LTCs, the latter being an indicator of complex multimorbidity)^111^.
- Caring circumstances (e.g., multigenerational households) and relationships (e.g., spouses, adult children) for carers
- Role and level of experience for HCPs

These characteristics will be determined from the demographic questionnaire that participants will be asked to complete following consent^112^. As an initial guide for participant recruitment across the different populations of interest, Table 3 shows the preliminary sampling frame developed, with the quotas of participants required in each category to guide recruitment^113^. For all groups, other important characteristics (e.g. sex, age, ethnicity, level of engagement with the intervention (carers and people with MLTCs) and location (areas of high and low socioeconomic deprivation for HCPs) will be monitored on an ongoing basis as recruitment and analysis progresses^112^.

Table 3 Preliminary sampling frame for the PERSONAL-AGILITY trial.

| **Population** | **Criteria** |  | **Proposed quota within the sample** |
| --- | --- | --- | --- |
| People living with MLTCs and frailty | Frailty status | Very mild/mild (CFS 4-5 or eFI >0.12–0.24) | 5 |
|  |  | Moderate  (CFS 6 or eFI >0.24–0.36) | 5 |
|  |  | Severe  (CFS 7 or eFI >0.36) | 5 |
| Carers | Caring circumstances and relationship | Spouse | 5 |
|  |  | Adult child | 5 |
|  |  | Part of a multi-generational household | 5 |
| Health and care professionals | Role | physical activity officer or associated role | 2-7 |
|  |  | Community provider or associated role | 7-12 |

## Recruitment and consent

### People living with MLTCs and frailty, and carer participants

People living with MLTCs and frailty and carer participants will be identified and recruited as described for the feasibility trial (see section 6.2). As part of the consent process for the feasibility trial participants will be informed that they may be contacted regarding their participation in an interview, but that this is optional, and they may decline to take part in this aspect of the study if they wish. The details of the interviews will be provided to participants in a separate participant information sheet to that used for the main study. People living with MLTCs and frailty and carers will be consented separately for the interviews.

### Health and care professionals

Eligible HCPs physical activity officers and other link workers or community providers involved in the intervention) will be approached by the qualitative research team and invited to take part in a semi-structured interview. Those interested in taking part will be provided with an information sheet. When a potential participant has indicated willingness to be interviewed, the researcher will arrange a convenient time and location to obtain informed consent.

## Consent procedures for the interviews

Consent procedures for the interviews will mirror those for the feasibility trial. Given the low-risk nature of the interviews, a proportionate approach to obtaining informed consent that allows for in person or online/telephone consent (as described in section 6.2) for the trial will be taken. This will allow a wider range of participants to take part and will also ensure that an undue burden is not created.

A PIS relating to the interviews will be provided (via email, post or in-person) to potential participants who have expressed an interest in participating in this part of the study. They will also be provided with the researchers’ contact details and encouraged to contact the researchers to discuss any questions or concerns they may have. The researchers will contact potential participants (via email, telephone, in person, or online via secure software e.g. Zoom or Teams) after at least 24-hours to discuss any questions or concerns they may have, confirm their eligibility, and to seek verbal consent to participate.

For in person interviews consent will be obtained by a trained researcher prior to commencing data collection. The researchers will begin by explaining the purpose of the study and details of what participation is required and then participants will be given the opportunity to ask questions and clarify their understanding before being asked to sign a consent form in the presence of the researchers; the participant will be given a copy of the information sheet and consent form to keep for their records and the researchers will keep a signed consent form.

For telephone or online individual interviews, participants will be talked through the consent form prior to commencing data collection. The researcher will initial the boxes in a bespoke consent form and sign on behalf of the participant. A copy of the completed form will be sent to the participant and the original retained in the trial master file. A separate consent form has been created for this process.

## Qualitative data collection for both people with MLTCs and frailty, and health and care professionals

One-to-one semi-structured interviews will be used to gather qualitative data as they offer an open and flexible method for exploring the participants’ individual experiences in-depth. For people living with MLTCs and frailty and their carers, interviews will explore:

- The acceptability of key trial procedures, outcome measures and exploration of any unintended harms and consequences
- The acceptability of the intervention including, where applicable:
  - exploration of the influence of interaction between participants who took part as a ‘dyad’
  - the inclusion of physical activity officer and engagement with community groups
  - barriers and facilitators to engagement
- potential mechanisms of intervention impact
- how contextual factors influenced intervention impact, including any unintended consequences

 Health and care professional interviews will explore:

- Their perceptions of PERSONAL-AGILITY, including its delivery and the need for any adaptations
- How contextual influences hindered or facilitated implementation

Basic demographic data, including (but not limited to) age, gender, ethnicity, profession and years of experience will be collected for HCPs. For individual participants interviews will occur after the 24-week visit or when participants withdraw from the study, if they remain willing to participate in an interview. Participants will have had sufficient experience of the PERSONAL-AGILITY intervention at this stage. Interviews with people with MLTCs and frailty and carer will be conducted separately to allow free expression of views.

Interviews will occur at three time points within the trial:

- After the first six months of recruitment, to identify initial challenges
- Midway through recruitment
- Within the last six months of the study, to identify any ongoing challenges.

Four different preliminary semi-structured topic guides have been developed reflecting the different groups we wish to include in the interviews: people living with MLTCs and frailty (appendix D), carers (appendix E) and physical activity officers (appendix F) and community providers (appendix G). These topic guides have been developed with the PPIE group and the TSG and guided by the PERSONAL-AGILITY programme theory and Normalisation Process Theory (NPT). Normalisation Process Theory is a middle-range implementation theory that has been used extensively to explore the processes underpinning implementation, embedding and integration of service innovation, and is well suited to multi-level intervention evaluation ^107^, ^106^. The first three interviews will act as pilots (but will be included within the overall analyses) and following these the guide may be adjusted. Guides will also be subject to adjustment as the trial progresses and the intervention is refined.

Interviews will be conducted by a trained researcher at an appointment separate from the other trial assessments. They will be arranged around participants’ personal and work commitments. Where possible, participants will be offered an opportunity to choose where the interview takes place either face-to-face, or remotely via telephone or secure online software. Interviews will be led by trained members of the research team. During the interviews, the researcher will introduce the process, explaining the background, how the interview will proceed, and details of audio-recording and note-taking. Participants will be reassured about the preservation of their anonymity and confidentiality as well as being given the opportunity to ask questions before audio-recording commences.

Interviews are anticipated to last approximately 60-90 minutes, depending on what participants wish to share, and the depth of discussion. Researchers will keep field notes during the interviews and observe non-verbal cues and facial expressions which may prompt further questions or follow-up. At the end of each interview, participants will be given another opportunity to ask questions, or seek clarification and the voluntary nature of their participation will be reiterated. Should any participant request further information or highlight any concerns during the interview then these will be discussed with the PI, for appropriate onward referral as required. All participants will be made fully aware of the contact details for the research team, should they have any concerns following the interview.

## Withdrawal criteria

Participants may withdraw from (a) complying with the allocated study treatment and/or (b) providing data to the study, at any time for any reason without affecting their usual care. Participants may withdraw from the process evaluation but remain within the main trial, should they wish. For participants who enrol as a dyad either individual may continue to be involved in the trial should the other person opt to withdraw.

In addition, the PI may discontinue a participant from the study at any time if considered necessary for any reason including:

- Discovered ineligibility which was overlooked at screening/baseline
- Major protocol deviation
- Significant non-compliance with treatment regimen or study requirements
- Participation in another intervention research study
- An AE which results in inability to continue to comply with study procedures
- Disease progression which results in inability to continue to comply with study procedures
- Prolonged or serious hospital admission (at the discretion of review by a study clinician)
- Development of any of the conditions or circumstances outline in the exclusion criteria

Withdrawals from the trial will be recorded in the CRF, database and medical records. Participants do not have to give a reason for withdrawal, however if they do provide a reason for leaving the study, this will be documented. If the participant is withdrawn due to an AE, the investigator will arrange for safety follow-up visits or telephone calls until the adverse event has resolved or stabilised.

They will be sent a letter thanking them for their participation and informing them that the data collected up to the time point they withdrew will be included in the trial analysis and that they will not be contacted again with regards to this trial, unless they consent to withdraw from active intervention but remain enrolled for subsequent follow-up. They will not be asked to complete any further study measures. A letter will also be sent to their GP to inform them of their withdrawal in the trial. For participants who are not contactable or fail to return questionnaires/accelerometer, the research team will make reasonable efforts to re-contact the participants (e.g., contacting informal carer if involved in the study or GP, reviewing available health care databases) and to determine their health status. Attempts to contact such participants will be documented in the participant’s records.

If a participant is withdrawn from the study due to a loss of capacity to provide informed consent, then all research data that has been collected prior to withdrawal will be retained. The participant will not be contacted again with regards to the trial, and no further follow-up data will be collected.

Each participant will have a copy of the consent form and participant information leaflet placed in their hospital medical records. A standard label will be used on the front of the medical notes to highlight to any reviewer that this individual is taking part in the study. As this is a feasibility study, one of the primary outcomes will be the proportion of, and reasons for withdrawal and therefore we will not be attempting to replace participants that withdraw.

## Assessment and management of risk

There are minimal risks associated with taking part in the PERSONAL-AGILITY study. Participants will be made fully aware of any potential risks before consenting. All research investigations are detailed in the participant information sheets and will be explained to the participant before each investigation to ensure that they are willing to undertake each one.

### Outcome testing

For frailer participants with lower functional ability, and who may be at increased risk of falls, objective tests of physical function such as the SPPB may make some people feel uncomfortable or unsteady, because it asks them to perform at a level that they may be unaccustomed to. Considering this, function tests will be performed in the presence of another person (staff, relative or carer where possible) and participants may defer the test if they do not feel safe. The participant will be able to cease testing whenever they wish.

For the measurement of movement behaviours, although the accelerometer is a lightweight and non-invasive device some participants may find these uncomfortable to wear over 9 days. Should this be the case, participants will be able to remove the device whenever they wish.

Finally, the PROMS may bring to light conditions and concerns requiring onward referral and ongoing support. PROMs used in both the trial and as part of the intervention will be reviewed immediately after collection and values raising concern will be discussed with the participant and, where necessary, flagged with their GP. Indicators of concern may include (but are not limited to):

- High levels of anxiety and depression
- New or worsening symptoms, including changes in health or new injury or trauma
- High levels of emotional distress
- High levels of carer burden

Testing conducted in participants homes may be subject to increased risk for the researcher. These risks will be minimised by adhering to the UHL lone working policy.

### Intervention

The study population will include people with MLTCs and frailty and consequently these participants may be at increased risk of falls or have a previous history of falls. Intervention providers and health and care practitioners will be made aware of falls risk and history and advice and interventions will be tailored to minimise the risk of future falls as far as possible.

The PERSONAL-AGILITY intervention will use digital technology in synergy with face-to-face contact to compliment and provide flexibility whilst maintaining the personal touch of direct face-to-face interaction between the service user and the HCPs. Whilst digital platforms are neither geographically-nor time-restrained and therefore may be particularly appealing for adults with MLTCs conditions and frailty and their carers, given their circumstances and priorities, there are potential risks associated. Steps for health includes a discussion forum, which could leave users open to adverse behaviours such as bullying, and provision of misinformation between users. To minimise any potential risks, we will utilise approaches from our previous programmes and extensive expertise in developing and monitoring online support groups. These include:

- Outlining an ‘acceptable use policy’ at registration
- Using an experienced moderator (someone who oversees the discussion forum on Steps for health and reviews content to ensure every post meets the site's standards, restricting any offensive behaviour, content, or writing. The whole platform including the chat forum is regularly checked/ monitored by designated LDC staff

As the PERSONAL-AGILITY study will use digital technology some participants may be at risk of digital exclusion. To limit the risk, technology will be explained in face-to-face appointments, and individualised support provided as required. Furthermore, participants will be given details (phone and email) of who to contact for IT support. Simple user guides will also be created to support use, in line with PPI feedback.

Where the intervention involves support from a physical activity officer, we will request that participants are only referred into safe and appropriate groups for their level of health and physical ability.

There may be some risks associated with becoming more active and sitting less. These include a slightly elevated risk of falls for people who have had a previous fall. Overall, previous research tells us that the benefits of being more physically active far outweigh the risks of active, and that in the long run sitting more does not make people at risk of falls safer. We will ensure that participants are well enough to take part in the programme as part of the screening process, and we will also make sure that they receive a programme which takes into account any previous falls you may have had to ensure that it is right for your needs. Some physical activities can be associated with short-term muscle aches which are normal, but can be uncomfortable, particularly if participants are not used to being physically active.

There are minimal risks associated with the interventions we will use to support participants to improve their sleep, but for some people with severe insomnia they may not be effective and could lead to frustration, or possibly worsening sleep habits. We will track this closely throughout the intervention, and refer these participants back to their GP if we suspect that they may need more in-depth support, or that they might have an underlying condition which requires treatment.

### Participation burden

Whilst the PERSONAL-AGILITY intervention aims to minimise the burden of treatment, using a person-centred, shared decision-making, approach, we acknowledge that any burdens associated with the intervention may only come to light during or after their involvement. To understand this, participants will be asked to participate in an interview as part of the mixed methods process evaluation. The focus of these interviews will be to explore the dynamic of user experience and implications for burden. We will also use a questionnaire to measure the 24-hour physical behaviours burden to further explore the burden of treatment associated with the PERSONAL-AGILITY intervention. This will allow further exploration of the burden of treatment within long term conditions, frailty and carer burden and complement the understanding gathered through our qualitative work.

Individuals with multiple long term conditions and frailty as well as their carers, often have complex lives, marked by a wide range of appointments and tasks associated with managing their conditions, or the conditions of those they care for ^114^. The time commitment needed to take part in this study may represent a burden to participants. The burden may be greatest to those within the intervention group, as this group will be asked to complete both the PERSONAL-AGILITY intervention and the assessment visits. This has been discussed extensively with the study PPI/E group, and consequently we will use a range of strategies to both understand and reduce the potential burden to participants. These include:

- Offering participants, the ability to opt out of the interviews
- Arranging study visits and interviews at the participant’s convenience, using a range of options (in person, telephone or online) to support participation and avoid the need for an additional visit to the hospital. Locations for in-person visits may also include the home and community locations where possible
- Organising the secondary PROM outcome in order of priority to the research team, and offering participants support to complete the PROMs
- Selecting only intervention measures which are relevant and important to the participant.

### Mixed methods process evaluation

Interviews will be in-depth and will follow a topic guide but also be semi-structured to allow for the exploration of other areas that are important to or significant for the participant, in relation to the research questions. Interviews may include discussion of health issues which significantly impact upon participants health and may mean that participants discuss emotive and distressing topics, or issues which they feel strongly about. The researcher will remain sensitive to the signs of distress and discomfort during the interviews and ask the participant if they wish to continue or would prefer to end the interview or move on to a new topic. In the case of significant distress, the participant will be referred to their care provider for further support.

The small number of participants involved in the qualitative study may lead to concerns about individual identification. Responses will be confidential, and data anonymised and this will be made clear to participants in the PIS, during the consent procedures and reiterated prior to the interview commencing. Finally, to enable assessment of fidelity, PERSONAL-AGILITY study sessions or assessments will be audio recorded. All participants will be asked for their consent to be recorded.

## End of trial

End of study will be defined as 12 months following the collection of outcome data at the 24 weeks visit of the last participant to allow sufficient time for sample analysis. The sponsor must notify the REC of the end of a clinical trial within 90 days of its completion.

# Recording and reporting of serious adverse events (SAEs)

## Definitions

Table 4.Definitions for SAEs

| **Term** | **Definition** |
| --- | --- |
| Adverse Event (AE) | Any untoward medical occurrence in a participant or clinical investigation, which does not necessarily have to have a causal relationship with this treatment.  An AE can therefore be any unfavourable and unintended sign (including an abnormal laboratory finding), symptom or disease temporally associated with the study, whether or not considered related to the study. |
| Serious Adverse Event (SAE) | A serious adverse event is any untoward medical occurrence that:   - Results in death - Is life-threatening* - Requires inpatient hospitalisation or prolongation of existing hospitalisation - Results in persistent or significant disability/incapacity - Consists of a congenital anomaly or birth defect - Other important medical events**   *The term "life-threatening" in the definition of "serious" refers to an event in which the participant was at risk of death at the time of the event; it does not refer to an event which hypothetically might have caused death if it were more severe.  **Other ‘important medical events’ may also be considered serious if they jeopardise the participant or require an intervention to prevent one of the above consequences. |

## Reporting procedures for all Adverse Events

AEs that are deemed directly related to study activities which are observed by the investigator or reported by the participant will be recorded in the participant’s medical records and an AE log from the time the participant is randomised. The following information will be recorded: description, date of onset and end date, severity, assessment of relatedness to study, and action taken. Additional follow-up information should be provided as necessary. AEs considered related to the study as judged by a medically qualified investigator or the sponsor will be followed until resolution, or the event is considered stable.  All related AEs that result in a participant’s withdrawal from the study or are present at the end of the study, should be followed up until a satisfactory resolution occurs.

It will be left to the investigator’s clinical judgement whether an AE is of sufficient severity to require the participant’s removal from treatment. A participant may also voluntarily withdraw from treatment due to what he or she perceives as an intolerable AE.  If either of these occurs, the participant must undergo an end of study assessment and be given appropriate care under medical supervision until symptoms cease or the condition becomes stable. The severity of events will be assessed using the following scale: 1 = mild, 2 = moderate, 3 = severe. AE’s will be recorded within the participant’s medical records and an AE log. Any safety concerns that arise because of this, will be reported to the sponsor as soon as possible.

## Expected Adverse Events and Serious Adverse Events

Due to the population of the study sample, the following events could be expected to occur throughout the duration of the trial and will therefore not be collected or reported to the sponsor:

- Outpatient appointments, including GP appointments, or treatments for ongoing conditions that were present at the start of the study

## Reporting Procedures for Serious Adverse Events

All SAEs (except from those outlined as expected) occurring from the time of randomisation until the final study visit must be reported to the Sponsor immediately and within 24-hours of becoming aware of the event. The SAE will be reported using appropriate forms and according to the Sponsor SOP for reporting serious adverse events. Additional information will be provided if requested to the Sponsor and main Research Ethics Committee (REC). The PI or a delegated physician (as agreed by the Sponsor) is responsible for the review and sign off of the SAE and the assessment of causality (i.e., whether an event is related to a study procedure or intervention).

The Sponsor will perform an initial check of the information and ensure that the SAE line listing is reviewed by the Director of Research & Innovation. All SAE information must be recorded on an SAE form and sent to the Sponsor. Additional information received for a case (follow-up or corrections to the original case) needs to be detailed on a new SAE form and sent to the Sponsor. Copies of all documentation and correspondence relating to SAEs will be stored in the trial master file (TMF).

For each **SAE** the following information will be collected:

- Full details in medical terms and case description
- Event duration (start and end dates, if applicable)
- Action taken
- Outcome
- Seriousness criteria
- Relationship to the study procedure or intervention

Any change of condition or other follow-up information should be emailed to the Sponsor immediately and within 24-hours of the information becoming available. Events will be followed up until the event has resolved or a final outcome has been reached.

# Statistics and data analysis

In line with a convergent parallel mixed methods design, quantitative and qualitative data will be analysed separately before being integrated within a mixed methods analysis.^115^.

## Sample size calculation

Determinations of sample size from a power calculation around a primary outcome are not relevant to a feasibility study^116^ ^117^. Sample sizes of 24-50 are generally considered sufficient to assess the key uncertainties relating to feasibility^118,119^ ^120^ ^121^ ^122^. A sample size of *n*=50 people living with MLTCs and frailty is appropriate to the aims of this study.

## Summary of baseline data and flow of participants

A CONSORT diagram, based on the extension to randomised pilot and feasibility trials, showing the flow of participants through the study will be produced^123^. Data will be checked for outliers and missing values and validated using the defined score ranges. Baseline demographics will be summarised by treatment group and for the total population using number (percentage) for categorical variables and mean (standard deviation) for continuous variables (unless they are found to be skewed in which case median and interquartile range will be presented). Clinically meaningful differences between groups at baseline will be described, but no statistical testing will be conducted to compare baseline characteristics by treatment group^124^. The basic baseline characteristics (age, gender, frailty status and ethnicity) of those who participated and declined will be explored where possible.

## Primary trial outcome analysis

The number and percentage of people living with MLTCs and frailty and carers:

- Screened
- Eligible
- Ineligible
- Consented
- Retained at 12 and 24 weeks

Will be calculated separately and described using descriptive statistics and 95% confidence intervals. and presented within a CONSORT diagram ^124^,^125^

## Progression criteria

Judgement regarding the feasibility of a future definitive trial and the acceptability of the trial processes and intervention will be established using a set of *a priori*  ‘progression criteria’ for each of the  feasibility and acceptability outcomes ^126^, ^121^. Failure to achieve these pre-established criteria will not necessarily indicate that a full-scale trial is not viable. For each criterion, the development of ‘stop’ and ‘go’ thresholds have been pre-specified ^127^. The ‘stop’ thresholds indicate when there are issues that cannot be resolved, and ‘go’ thresholds when there are no issues that may impede the success of a trial. Results falling between these thresholds indicate that ‘change’ is required, and will allow the research team to identify where there are issues that may be remedied, rendering a definitive RCT viable^121^, ^127^. Overall decisions about the viability of a future definitive trial will be informed by all available data (including process data), as outlined in section 9.7.

Table 5.Progression criteria

|  | **GREEN** | **AMBER** | **RED** |
| --- | --- | --- | --- |
| **Eligibility** | ≥ 70% of people living with MLTCs and frailty are eligible | 69-51% of people living with MLTCs and frailty are eligible | ≤ 50% of people living with MLTCs and frailty are eligible |
| **Recruitment** | ≥ 50% of people living with MLTCs and frailty who were eligible consented to take part in the trial | 49-29% of people living with MLTCs and frailty who were eligible consented to take part in the trial | ≤ 30% of people living with MLTCs and frailty who were eligible consented to take part in the trial |
|  | ≥ 50% of people living with MLTCs and frailty identify a carer who is subsequently recruited into the trial | 49-19% of people living with MLTCs and frailty identify a carer who is subsequently recruited into the trial | ≤ 20% of people living with MLTCs and frailty identify a carer who is subsequently recruited into the trial |
|  | ≥ 20% of participants are from a minority ethnic background | 19-6% of participants are from a minority ethnic background | ≤ 5% of participants are from a minority ethnic background |
|  | ≥ 30% of participants are from socioeconomically deprived backgrounds.  These rates reflect the general population ^128^, ^129^. | 29-8% of participants are from socioeconomically deprived backgrounds. | ≤ 7% of participants are from socioeconomically deprived backgrounds. |
| **Retention** | ≥ 70% of participants retained at 24 weeks | 69-51% of participants retained at 24 weeks | ≤ 50% of participants retained at 24 weeks |
| **Intervention implementation** | ≥ 70% of face-to-face intervention reviews were complete without additional correspondence from the intervention providers | 69-51% of face-to-face intervention reviews were complete without additional correspondence from the intervention providers | ≤ 50% of face-to-face intervention reviews were complete without additional correspondence from intervention providers |
| **Intervention engagement** | Enacts the agreed treatment plan 3 times a week or more on average across the intervention period | Enacts the agreed treatment plan twice weekly on average across the intervention period | Enacts the agreed treatment plan once a week on average across the intervention period |
| **Outcome measure completion** | ≥ 80% of outcome measures completed at 12 and 24 weeks | 79-59% of outcome measures are completed at 12 and 24 weeks | ≤ 60% of outcome measures completed at 12 and 24 weeks |

## Secondary trial outcome analysis

Outcomes at 12- and 24-weeks follow-up will be summarised by randomised group and overall using mean (SD) and median (IQR) for continuous variables and count (percentage) for categorical variables. The number of SAEs will be presented overall and by randomised group.  All exploratory analyses will be considered hypothesis generating rather than providing firm conclusions and no p values will be reported.

Outcome acceptability will be determined by quantifying the amount of missing data across the secondary outcomes. The proportion of missing data (number, %) will be reported for each outcome, but no imputation will be performed to account for this missing data. Reasons for missing data will be gathered where possible.

Quantitative acceptability data including:

- rates of uptake and engagement with each component of the PERSONAL-AGILITY intervention
- adherence to the intervention (e.g., frequency, intensity, time and type of activity)

will be reported descriptively using number and percentage.

PROMS relating to intervention acceptability (the 9-item shared decision-making questionnaire) will be examined descriptively, by reporting at 12- and 24-weeks follow-up using mean (SD) and median (IQR).

## Analysis of the mixed methods process evaluation

### Quantitative analysis

Data from the structured observation tool will be analysed using descriptive statistics. Number (percentage) will be used for categorical variables and mean (standard deviation) for normally distributed continuous variables Skewed data will be reported using medians and interquartile ranges.

Other quantitative data from processes and PROMS relating to intervention implementation (the 24-hour Physical Behaviour Burden Questionnaire) will be examined descriptively, by reporting at 12- and 24-weeks follow-up using mean (SD) and median (IQR).

### Qualitative analysis

Qualitative data will be collected and analysed concurrently, to allow iterative refinement of the intervention to occur ^89^, ^115^. Reflexive thematic analysis will be used to guide qualitative data analysis ^130^. Researchers will independently familiarise themselves with the interview data by listening to the audio-recordings and reading and re-reading the transcribed data.

Reflexive thematic analysis involves six sequential but iterative stages where the research can move back and forth recursively as new insight is gathered and links between data made:

- Familiarisation: the research team will read and re-read the data to become familiar with the data, making notes and analytic observations and insights.
- Coding: Succinct labels (codes) that capture and evoke important features of the data that might be relevant to addressing the research question will be devised. These will be agreed with the research team through discussion before being applied across the dataset. Multiple rounds of coding may be undertaken and codes revised through this process.
- Generation of initial themes: Codes will be examined and collated into broader patterns of meaning (potential themes). Data relating to each candidate theme will be collated.
- Developing and reviewing themes: Candidate themes will be checked against the coded data and the entire dataset, to determine fit with the data and ability to answer the research questions. Themes will be further developed, combined, or discarded during this phase.
- Refining, defining and naming themes: TA detailed analysis of each theme will be developed, establishing the scope and focus of each. Names will be given to each theme.
- Writing up: Data will be combined together with analytic narrative before being contextualised in relation to existing literature.

The intervention programme theory, and NPT (as previously described) will provide a sensitising framework to guide analysis ^107, 106,^ alongside the recently published coding manual ^107, 106^.

## Mixed methods analysis

Following separate qualitative and quantitative data analyses for both the trial and process evaluation, all results will be integrated and compared against the progression criteria (section 9.4) to facilitate a comprehensive understanding of: ^131, 132, 115, 133^

- the acceptability of the PERSONAL-AGILITY intervention
- refinement of the initial intervention programme theory
- the feasibility of a definitive trial of this intervention
- optimisation of the definitive trial design.

Integrated analyses will be facilitated by a ‘joint display’, which combines the findings in a tabulated form. This display may be organised according to the primary feasibility outcomes and will be used to assess the ways in which the data sets agree (confirmed), complement (offered an expanded explanation) or contradict each other and outline how the qualitative results expand and contextualise the key results from the quantitative results ^133, 134^.

Following this, decision-making relating to progression to a definitive trial, will be facilitated by the ADePT (*A process for Decision-making after Pilot and Feasibility Trials)* tool, which was developed to make decision-making regarding feasibility explicit^135^. Briefly, this process includes:

- Systematically identifying potential concerns surrounding the feasibility of a definitive trial and assigning them to a specific typology (Type A: a problem only for the trial, Type B: a problem for the trial and in the real-world, Type C: a problem only for the real-world)
- Identifying the range of potential solutions alongside supporting evidence
- Assessment of the best solution available.

# Data management

## Data Flow Diagram

The below data diagram outlines the flow of data in the trial and the process evaluation. In summary, data collected at the NHS research site will be uploaded onto a secure trial database (RED Cap). Following database lock, feasibility, acceptability and qualitative and quantitative process evaluation will be conducted.


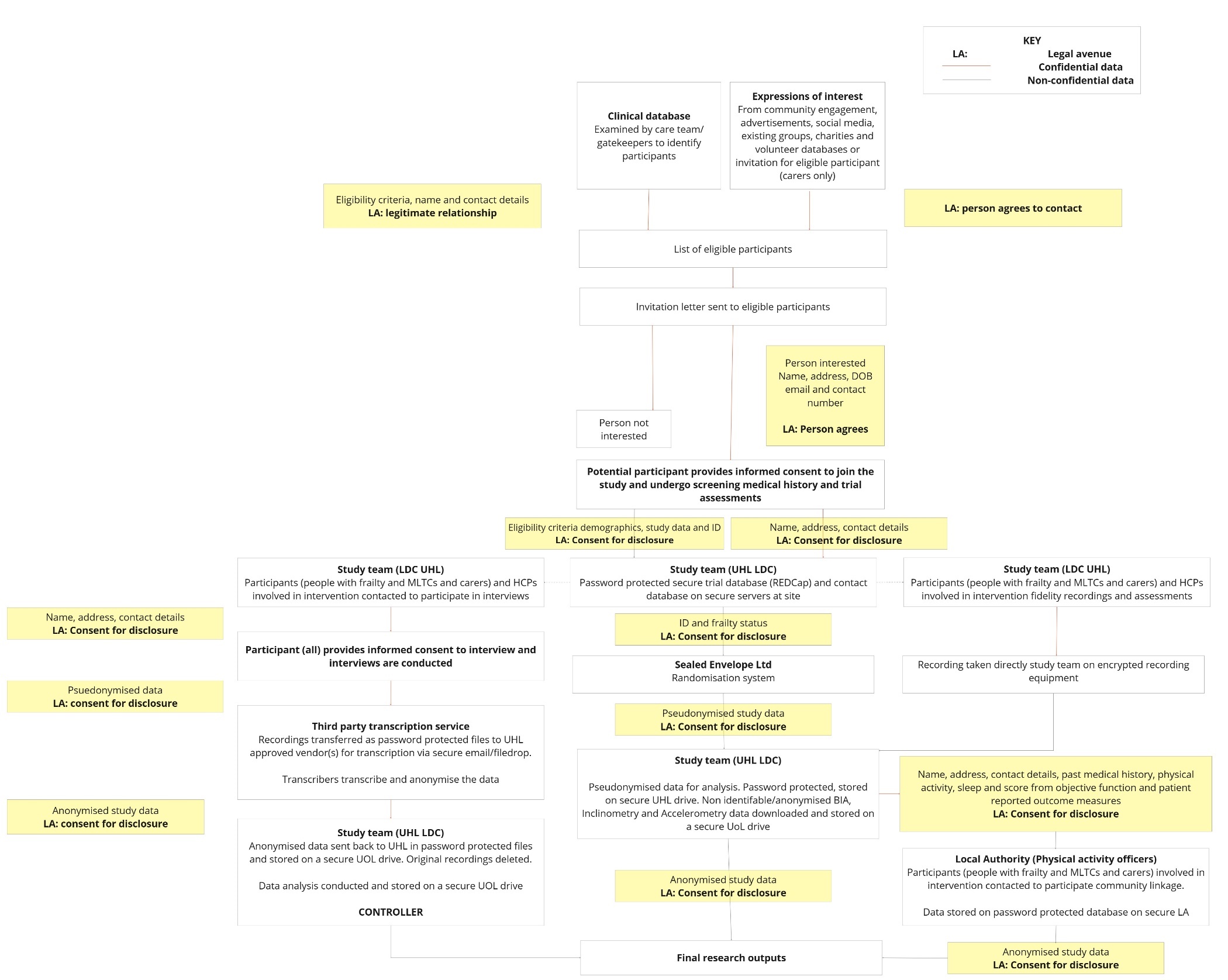


## Data collection and management

All hard and electronic copies of data collected in this study will be identified by a unique study identification code. The link between the participant and their study ID number will be retained by the host NHS organisation in a locked filing cabinet in a secure office environment and with access restricted to members of the research team. All electronic data will be password protected and accessible only by delegated members of the research team during the active phase of the study and until the data have been analysed. Paper copies such as CRFs will be stored in a locked cabinet.

All research data, whether online or paper-based, will be transcribed onto the research database (REDCAP) by a delegated member of the research team. All study documentation containing identifiable participant data will be pseudonymised and managed in accordance with ICH-GCP, UK Policy Framework for Health and Social Care Research and the most up-to-date version of the Data Protection Act 2018 and General Data Protection Regulation (GDPR). Anonymised and non-identifiable body composition, inclinometer and accelerometer data will be downloaded, and analysis facilitated, using bespoke software available via the University of Leicester, and therefore these anonymised non identifiable files will be stored on a secure University of Leicester R drive.

Where applicable, to facilitate Physical Activity Officers to identify appropriate and suitable community groups for participants, connect them with these groups, monitor their engagement with these groups and support them where needed, participant names, contact information (email, telephone number, address) and relevant health data (relevant past medical history, physical activity, sleep and score from objective function and patient reported outcome measures) will be shared between UHL and the relevant local authorities who employ the physical activity officers. This will facilitate the delivery of this component of the PERSONAL-AGILITY intervention. Data will be subject to GDPR and will be transferred between the controller and processor by secure email to a verified local authority email.

Personal identifiable information will not be included in any research database. Participant identifiable information will be destroyed 12 months after completion of the study unless participants have consented to be contacted about future research. If a participant wishes to withdraw, they will be documented as a withdrawal and their personal identifiable data will be destroyed, unless they have consented to be contacted about future research.

For the observations and qualitative interviews within the process evaluation, willingness to continue will be confirmed verbally prior to the start of the observation/interview. Audio recordings of interviews will be labelled with a study identifier and will be destroyed once transcription is complete. Data will be transferred to the transcription service using secure file transfer. Transcripts will be anonymised and securely stored six years post-study duration. Audio recordings of observations will be labelled with a study identifier and destroyed after the observational tool has been completed. Completed observational tools will include no identifiable data and will be securely stored for six years post-study duration. Qualitative data analyses will be facilitated by NVivo and therefore anonymised transcripts will be securely stored on University of Leicester R Drives.

Study data will be archived in line with UHL policy; currently six years post-study duration. Data management will be delegated to trained members of the research team and overseen by the CI. Quality control checks of the source data and data entered into the trial database will also be undertaken by the research team.

Participants who do not already use wearable technology to track their activity, sleep and sedentary behaviour will be provided with a FitBit for the duration of the intervention. In order to link Steps4health and MyHealthMapp to the FitBit, they will be required, and supported to, set up an account with FitBit. This will require them to supply their name, email address, password, date of birth, gender, height, weight. They will then be asked to sign into their account and select what elements of their data they would like to link to Steps4Health and MyHealthMapp from their FitBit.

FitBit is able to determine precise geolocation data for select features, including location from GPS signals, device sensors, Wi-Fi access points, cell tower IDs and IP addresses. This type of data is collected if the participant grant access to their location. This information is not required to participate in the intervention and participants will be informed that they can remove access using your Fitbit device or mobile device settings. Fitbit retains the data, collected by the device until the participant chooses to delete it or discontinue using Fitbit services. The participant has the right to access, manage, and delete the data collected by the FitBit. Through their account settings, they can remove specific data types or delete their account entirely, which will initiate the process of erasing their data from Fitbit's systems. Participants will be informed of this during the consent process and also at the end of the intervention, so that they may delete their FitBit account if they wish. This will not influence the data collected via MyHealthMapp and Steps4Health, which will be retained for the purposes of the study.

## RED Cap (trial database)

RED Cap is a web application which should run on any PC with a modern browser. For details and documentation about the software, please see: [https://www.project-redcap.org](https://eur03.safelinks.protection.outlook.com/?url=https%3A%2F%2Fwww.project-redcap.org%2F&data=05%7C01%7Chy162%40leicester.ac.uk%7C6a9690cb8eac475143e208dab10e0a3c%7Caebecd6a31d44b0195ce8274afe853d9%7C0%7C0%7C638016971113613524%7CUnknown%7CTWFpbGZsb3d8eyJWIjoiMC4wLjAwMDAiLCJQIjoiV2luMzIiLCJBTiI6Ik1haWwiLCJXVCI6Mn0%3D%7C3000%7C%7C%7C&sdata=1tcPcWeZZo27QIINiMV6MZ3oyCEFdq1mgAESyhuqtgM%3D&reserved=0). The application is hosted on a University of Leicester virtual 'LAMP' server. The physical servers are located at the University of Leicester main site. Servers are backed up nightly and the backups are sent to a University of Leicester remote site. Physical access to the server’s access is restricted to IT Services and Estate Staff. Access to the Operating System is restricted to the NIHR Leicester Biomedical Research Centre IT and University of Leicester Research Computing Support teams. Servers are monitored and regularly patched for security vulnerabilities. The servers are regularly penetration tested using Nessus. Connections to the server pass through a reverse proxy, that strips out requests and request content which may compromise security. Information on the University of Leicester information governance can be found at [https://bit.ly/3fqXxda](https://eur03.safelinks.protection.outlook.com/?url=https%3A%2F%2Fbit.ly%2F3fqXxda&data=05%7C01%7Chy162%40leicester.ac.uk%7C6a9690cb8eac475143e208dab10e0a3c%7Caebecd6a31d44b0195ce8274afe853d9%7C0%7C0%7C638016971113613524%7CUnknown%7CTWFpbGZsb3d8eyJWIjoiMC4wLjAwMDAiLCJQIjoiV2luMzIiLCJBTiI6Ik1haWwiLCJXVCI6Mn0%3D%7C3000%7C%7C%7C&sdata=ZN3M%2BR41rGB4%2FUGiQEfCUX4QCFlvmE95g8oYlqOtm%2FE%3D&reserved=0). Authentication is provided by the application. Users log in with a username and password. Passwords must be at least 9 characters and must consist of at least one lower-case letter, one upper-case letter, and one number. The application is visible to computers on the Internet. All communication between client computers and the application are encrypted using HTTPS.

## Access to data

Source data, study documents, and participant notes will be made available for monitoring, auditing and inspections by the appropriate regulatory authorities, the Sponsor, and NHS host organisation.

All study documentation will be retained in a secure location during the conduct of the study. Personal identifiable data will be retained by each participating site for a maximum of 12 months following the end of the study, after which it will be destroyed, unless participants have expressed an interest in being invited to the results dissemination events and/or being contacted about participation in other research. In these circumstances, personal identifiable details such as names and contact details will be retained on a password protected database until required, and then destroyed. If the participant consented to being contacted about future research, then their data will remain in the LDC volunteer’s database. All electronic data will be stored on secure network systems, to which only the relevant study personnel will have access. For the purposes of this study, the UHL will act as the Data Controller for data for both the trial, and process evaluation. NVivo (used to manage the qualitative interviews) is encrypted and only the account owner has access to and control over the data.

## Archiving

Archiving of the study data analysis, data and essential study records will be authorised by the Sponsor following submission of the end of trial report. Personal identifiable data generated by the study will be retained for the minimum time determined by the regulatory authorities following the notification of the end of the study before being destroyed. Documents will be archived in a secure location for a minimum of six years after the completion of the study, in accordance with UHL SOPs. The data will be archived at a Sponsor approved archiving facility. No study-related records, including hospital medical notes, will be destroyed unless or until the Sponsor gives authorisation to do so.

# Monitoring, order and inspection

The University Hospitals of Leicester NHS Trust, as Sponsor, operates a risk-based monitoring and audit programme, to which this study will be subject.

# Ethical and regulatory considerations

## Research Ethics Committee (REC) review and reports

Once the initial sponsor review process is complete and a sponsor reference number has been allocated, and all requested documentation has been received and checked authorisation from the UHLs Research and Innovation Office will be issued to book further review of the proposed research. The protocol, informed consent form, PIS, interview topic guides, questionnaires and any proposed advertising material will be submitted to an appropriate REC, HRA, and host institution(s) for written approval. Agreement in principle is subject to the research receiving all relevant regulatory permissions. Submission for regulatory approvals will be submitted via Integrated Research Application System (IRAS). The CI will ensure that all regulatory approvals, confirmation of capacity and capability and sponsor greenlight are in place before participants are approached. The Research and Innovations Office’s SOPs will be followed for the duration of the trial. A trial master file will be maintained for the duration of the study and will be stored for six years after the study has ended. All correspondence with the REC will be retained in the TMF.

Amendments will be submitted to the sponsor in the first instance for review and approval. The CI, in agreement with the sponsor, will then submit information to the appropriate body for them to issue approval for the amendment. Amendments will be implemented upon receiving Sponsor Green Light.

If the study is ended prematurely, the CI will notify the REC, including the reasons for the premature termination. Otherwise, the CI (or delegate) will notify the REC of the end of the study. Within one year after the end of the study, the CI (or delegate) will submit a final report with the results, including any publications/abstracts, to the REC.

This study will be conducted in full conformity with the current revision of the Declaration of Helsinki (last amended October 2000, with additional footnotes added 2002 and 2004) and the UK Policy Framework for Health and Social Care Research (2017). It will also be conducted according to ICH-GCP relevant regulations.

# Peer review

Prior to funding, this work underwent extensive external review by two independent reviewers as part of the process of obtaining external funding from the NIHR. Prior to submission the protocol has also undergone external review by the study collaborators, internal review within the Leicester Diabetes Research Centre, and the trial sponsor (UHL). Appropriate amendments have been made in response to all reviews.

# Lived experience group involvement

## Overview

A PPI/E group has been convened and will guide the delivery of the research activities. To date, eighteen diverse carer and PPI/E Representative(s) have shared their experiences of living with, or caring for someone, with MLTCs and frailty from the inception of this project. They were actively involved in preparing the funding application for this study, pre-award, and they have been involved in the development of this protocol and the outline topic guides. Of note is the need for a flexible approach to data collection, which fits around the needs of people with MLTCs and frailty and those who care for them and is cognisant of the burdens and demands already placed upon them. PPI/E members have reviewed all participant facing documentation, lay summaries and have shaped the dissemination strategy outlined below.

## PPI/E strategy

The PPI/E strategy developed has been informed by NIHR Standards for Public Involvement, and guidance on involving carers in research^136^ both of which will continue to provide a framework for expected standards^137^.

To ensure inclusivity, we will work closely with the Centre for Ethnic Health Research (CEHR) and BRC director of inclusion to implement strategies used to successfully engage with seldom heard, under-represented groups. This includes the involvement of a community engagement officer, working independently of the research team, as part of the CEHR Engagement Team and based in the community. They, together with researchers involved in the study, will attend community-based activities (events, festivals etc.), to establish and maintain partnerships, and overcome barriers to engagement. They will facilitate initial and (where needed) ongoing recruitment to PPI/E group, and support retention by maintaining contact throughout each individual’s involvement.

PPI/E meetings will be held regularly, with additional *ad hoc* meetings as required.  Representatives want to be able to opt-in and out of involvement based upon their availability and health. Where members wish to contribute, but cannot attend, they will be consulted individually. As the diverse needs and nature of this group may mean that not everyone can attend meetings, representatives will be kept informed of discussions and decisions made, and offered the opportunity to input if they wish.

Ground rules will be developed by all PPI/E representatives to clarify the expectations, roles, responsibilities and behaviours from the outset ^138^ ^139^. Methods to maintain continuing dialogue between stakeholders will also be discussed and agreed ^139^. PPI/E activities will be flexible to enable a wide range of people with differing needs and abilities to participate equally and in ways in which are meaningful and important to them. PPI/E members involved in the development of this protocol emphasised the importance of ensuring sufficient time for a PPI/E group inclusive of a diverse group with varying cultural and religious requirements and functional, cognitive, and sensory impairments to meaningfully contribute.

For all activities, we will ensure a pragmatic approach to scheduling is adopted. Meetings will be offered at a variety of times, days and in a range of formats to accommodate the needs and preferences of a diverse range of people. In-person meetings will be held in accessible locations to accommodate those with mobility issues and resources and materials adapted to those with sensory impairments. Members will be recognised for their time and expertise and reimbursed in accordance with NIHR guidance. Where required, all members will be offered training individualised to their needs and experience to develop their skills and confidence.

Building and maintaining relationships with stakeholders, including understanding personal reasons and motivations for collaboration to promote personalisation is a central tenet of co-production. This relationship building has already begun and will be maintained through the project through regular communications and social events where possible. Mutual sharing of knowledge and skills for the benefit of all involved leads to greater collaboration.^192, 193^ Reciprocity will be achieved through power sharing, joint decision making (where possible) and the provision and training and support where needed. In addition, the research team, and associated clinical and academic teams, will be encouraged to share their knowledge in ways which would benefit the PPI/E group, for example providing seminars on 24-hour physical behaviours and linking people with other networks.

## Evaluation

To promote evaluation of the PPI/E activities and their impact, all activities will be captured and evaluated using impact and reflection logs (after every activity), and the Public and Patient Engagement Evaluation Tool annually^140^. Evaluations and reflection will be used during the project to guide changes to our proposed strategy.

# Regulatory compliance

The trial will not commence until Favourable REC opinion. Before enrolling participants into the study, the CI/PI or designee will ensure that appropriate approvals are in place.

# Protocol compliance

A protocol deviation is defined as any unintended change or departure from the protocol which does not result in harm to the study participants or significantly affect the scientific value of the study. Minor deviations can occur frequently during the study. Visit window deviations will be considered minor deviations as they do not have the potential to cause harm to the participant or impact the integrity of the study. Participants unable or unwilling to complete assessments for secondary or exploratory outcomes will still be included within the study, providing that as a minimum, they are able to provide feasibility outcome data. We will document and log any missing data, but secondary or exploratory outcomes not completed will not be recorded as a protocol deviation.

Major deviations are events that cause or could cause harm to participants or others or that affect the fidelity of the research. Where deviations frequently reoccur, this may meet the criteria for a Serious Breach of GCP and will be reported in line with Sponsor SOPs. For the purposes of this regulation, a ‘serious breach’ is a breach which is likely to affect to a significant degree:

- The safety or physical or mental integrity of the participants of the study; or
- The scientific value of the study

Prospective, planned deviations or waivers to the protocol will not be allowed. Accidental protocol deviations can happen at any time. They must be adequately documented on the relevant forms and reported to the CI and Sponsor immediately. The study team will monitor and review protocol compliance. If a protocol breach occurs, then the PI will document this in adherence to the UHLs SOP Identifying and Reporting Deviations and Serious Breaches of GCP and/or the Protocol for Trials. The PI will seek advice from the sponsor as required.

# Data protection and confidentiality

The PI will have access to the trial documentation and will be the data custodian. Participants’ personal data included in study-related databases shall be treated in confidence and in compliance with ICH-GCP, the UK Policy Framework for Health and Social Care and the EU GDPR. When processing or archiving personal data, the Sponsor or its representative shall take all appropriate measures to safeguard and prevent access to this data by any unauthorised third party. All investigators and trial staff will comply with the requirements of the Data Protection Act/GDPR with regards to the collection, storage, processing and disclosure of personal information and will uphold the Act’s core principles. They will also keep up to date with organisational training in relation to information governance.

Personal identifiable information will be held only on the delegation log. Once written or recorded verbal consent has been obtained the participant will be allocated a study participant code which will be used on all documentation to anonymise the participant and avoid the use of personal identifiable data. The Trial Master File will be kept in a folder in a locked cabinet/drawer in a secured room in a secure office environment at UHL by the Chief Investigator. Storage will adhere to organisational policy on storage.

All electronic participant identifiable information will be held on a secure, password-protected database accessible only to essential personnel. All paper-based data will use the participant code and be kept in a locked filing cabinet in a secure office environment office at UHL, and will only be accessed by essential members of the research team and the minimum number of individuals necessary for quality control, and analysis. Neither hard copies nor electronic files containing personal information will be removed from the research office or stored in a non-secure manner electronically. Direct access to source data / documents will be required for study-related monitoring.

Interviews will be undertaken using an encrypted USB digital recorder. Audio recordings will be transferred to password protected files, immediately after each interview and will then be deleted immediately from audio-recorders. A back-up copy of the audio file will also be kept in a separate password protected file in case the working copy becomes corrupted. Transcription will be undertaken by approved professional transcription service providers. All electronic records/data will be coded using the anonymised participant study code. Data files (e.g., transcripts and audio recordings) will be password protected to ensure data protection during transfer to and from the researchers and transcribers and will use the allocated participant study ID rather than personal details. The passwords will be provided to the transcribers/researchers in separate email/phone messages to minimise the risk of unauthorised access to the data.

Personal identifiable data will be retained for a maximum of 12 months following the end of the study, after which it will be destroyed, unless participants have expressed an interest in being invited to dissemination events and/or being contacted about participation in other research. In these circumstances, personal identifiable details such as names and contact details will be retained on a password protected database until required, and then destroyed. If the participant consented to being contacted about future research, then their data will remain in the Leicester Diabetes Centres (LDCs) volunteer’s database.

Anonymised research data from all sources will be stored for six years after the study has ended. Long-term storage will comply with the UHL archiving SOP. The PI will be the data custodian and be responsible for ensuring they are stored securely (password protected electronic files, paper files stored in locked filing cabinets). Access to the files or use of the data will be requested through the PI. Analysis of the generated data will be undertaken by the PI or designated investigators. All collected data will be saved on secure drives at UHL.

Direct quotations from participants may be published as part of publications arising from this work, but these will be anonymous, their identity will not be revealed, and quotations used will not contain any information which may inadvertently reveal the identity of any participant.

# Indemnity

Sponsorship and insurance for the study will be provided by UHL. In the event that something does go wrong and a participant is harmed during the research and this is due to someone ‘s negligence then they may have grounds for a legal action for compensation against University of Hospitals of Leicester NHS Trust but they may have to pay their legal costs. The normal National Health Service complaints mechanisms will still be available to them (if appropriate). If a study participant wishes to make a complaint about any aspects of the way they have been treated or approached during the research project, the standard NHS complaint system will be available to them. Details of this are made available to participants within the PIS.

# Post trial care

Provisions will be put in place for post-trial access for all participants who still need an intervention identified as individually beneficial. Participants will be referred on to local services and physical activity providers which meet their needs and interests as part of the intervention. At the final review participants will also be informed that they may continue to use the tools outlined on an ongoing basis. Usual care control participants will be shown how to access Steps4Health should they wish to access this component of the programme.

# Access to final trial data set

The CI will have access to the full dataset. Direct access will be granted to authorised representatives from the Sponsor and host institutions for monitoring and/or audit of the study to ensure compliance with regulations.

# Dissemination policy

The PI will be responsible for ensuring the results of the study are disseminated through peer review journals, conference presentations and other local mechanisms. Dissemination will be supported by researchers involved in the study, local, and national infrastructure (including NIHR Leicester and Newcastle BRCs, East Midlands Applied Research Collaborative (EM-ARC), and the CEHR) and NIHR dissemination support structures (including NIHR INVOLVE). Communications will be supported by the communications leads for the University Hospitals of Leicester NHS Trust and the local NIHR Regional network, and their research partnerships.

Results will be disseminated to community groups, charities and EM-ARC PPI/E channels and social media via a range of different formats, including brief written reports, infographics, and short animated videos with translated subtitles. Ongoing review and revision of our dissemination strategy will be undertaken throughout the study, in partnership with PPI/E partners, to maximise impact and ensure relevance and accessibility to a wide range of people. Where possible, materials will be available in languages reflective of the local community to reach a diverse audience, inclusive of those with cognitive, sensory and communication impairments The public will be informed through public lectures, radio, and podcasts.

All participants will receive a written report and will have access to the range of dissemination activities outlined above.

# Authorship eligibility guidelines and any intended use of professional writers

Authorship will be determined by the PI according to contribution to the study and the International Committee of Medical Journal Editors guidelines. All publications will acknowledge the participating co-investigators, the Sponsor and the NIHR. Guidance provided by the NIHR in relation to dissemination and publication will be adhered to. Relevant reporting guidance and checklists will be reviewed prior to generating any publications to ensure they meet the standards required for submission to high quality peer reviewed journals.

# References

1. The Academy of Medical Sciences. Multimorbidity: a priority for global health research. *The Acad Med Sci*. 2018:1-127.

2. Kingston A, Robinson L, Booth H, Knapp M, Jagger C, Project M. Projections of multi-morbidity in the older population in England to 2035: estimates from the Population Ageing and Care Simulation (PACSim) model. *Age and ageing*. 2018;47(3):374-380.

3. Stafford M, Steventon A, Thorlby R, Fisher R, Turton C, Deeny S. *Briefing: Understanding the health care needs of people with multiple health conditions*. Health Foundation London; 2018.

4. Skou ST, Mair FS, Fortin M, et al. Multimorbidity. *Nature Reviews Disease Primers*. 2022;8(1):48.

5. Rosbach M, Andersen JS. Patient-experienced burden of treatment in patients with multimorbidity–a systematic review of qualitative data. *PloS one*. 2017;12(6):e0179916.

6. Clegg A, Young J, Iliffe S, Rikkert MO, Rockwood K. Frailty in elderly people. *The lancet*. 2013;381(9868):752-762.

7. Nicholson K, Griffith LE, Sohel N, Raina P. Examining early and late onset of multimorbidity in the Canadian Longitudinal Study on Aging. *Journal of the American Geriatrics Society*. 2021;69(6):1579-1591.

8. Vetrano DL, Rizzuto D, Calderón-Larrañaga A, et al. Trajectories of functional decline in older adults with neuropsychiatric and cardiovascular multimorbidity: a Swedish cohort study. *PLoS medicine*. 2018;15(3):e1002503.

9. Hanlon P, Fauré I, Corcoran N, et al. Frailty measurement, prevalence, incidence, and clinical implications in people with diabetes: a systematic review and study-level meta-analysis. *The Lancet Healthy Longevity*. 2020;1(3):e106-e116.

10. Han L, Clegg A, Doran T, Fraser L. The impact of frailty on healthcare resource use: a longitudinal analysis using the Clinical Practice Research Datalink in England. *Age and ageing*. 2019;48(5):665-671.

11. Public Health England. Wider impacts of COVID-19 on physical activity, deconditioning and falls in older adults. Public Health England London; 2021.

12. Cruz-Jentoft AJ, Sayer AA. Sarcopenia. *The Lancet*. 2019;393(10191):2636-2646.

13. Dodds RM, Granic A, Robinson SM, Sayer AA. Sarcopenia, long‐term conditions, and multimorbidity: findings from UK Biobank participants. *Journal of cachexia, sarcopenia and muscle*. 2020;11(1):62-68.

14. Taylor RS, Singh S. Personalised rehabilitation for cardiac and pulmonary patients with multimorbidity: Time for implementation? *European journal of preventive cardiology*. 2021;28(16):e19-e23.

15. Cieza A, Causey K, Kamenov K, Hanson SW, Chatterji S, Vos T. Global estimates of the need for rehabilitation based on the Global Burden of Disease study 2019: a systematic analysis for the Global Burden of Disease Study 2019. *The Lancet*. 2020;396(10267):2006-2017.

16. Holland AE, Harrison SL, Brooks D. Multimorbidity, frailty and chronic obstructive pulmonary disease: Are the challenges for pulmonary rehabilitation in the name? *Chronic Respiratory Disease*. 2016;13(4):372-382.

17. Flint KM, Stevens-Lapsley J, Forman DE. Cardiac rehabilitation in frail older adults with cardiovascular disease: a new diagnostic and treatment paradigm. *Journal of cardiopulmonary rehabilitation and prevention*. 2020;40(2):72-78.

18. de Rooij M, van der Leeden M, Cheung J, et al. Efficacy of tailored exercise therapy on physical functioning in patients with knee osteoarthritis and comorbidity: a randomized controlled trial. *Arthritis care & research*. 2017;69(6):807-816.

19. Barker K, Holland AE, Lee AL, et al. A rehabilitation programme for people with multimorbidity versus usual care: a pilot randomized controlled trial. *Journal of comorbidity*. 2018;8(1):2235042X18783918.

20. Khunti K, Highton PJ, Waheed G, et al. Promoting physical activity with self-management support for those with multimorbidity: a randomised controlled trial. *British Journal of General Practice*. 2021;71(713):e921-e930.

21. De Vries N, Van Ravensberg C, Hobbelen J, Rikkert MO, Staal J, Nijhuis-Van der Sanden M. Effects of physical exercise therapy on mobility, physical functioning, physical activity and quality of life in community-dwelling older adults with impaired mobility, physical disability and/or multi-morbidity: a meta-analysis. *Ageing research reviews*. 2012;11(1):136-149.

22. Hanlon P, Nicholl BI, Jani BD, Lee D, McQueenie R, Mair FS. Frailty and pre-frailty in middle-aged and older adults and its association with multimorbidity and mortality: a prospective analysis of 493 737 UK Biobank participants. *The Lancet Public Health*. 2018;3(7):e323-e332.

23. Yuan L, Chang M, Wang J. Abdominal obesity, body mass index and the risk of frailty in community-dwelling older adults: a systematic review and meta-analysis. *Age and Ageing*. 2021;50(4):1118-1128.

24. Tuttle LJ, Bittel DC, Bittel AJ, Sinacore DR. Early-onset physical frailty in adults with diabesity and peripheral neuropathy. *Canadian journal of diabetes*. 2018;42(5):478-483.

25. Ford JC, Ford JA. Multimorbidity: will it stand the test of time? *Age and ageing*. 2018;47(1):6-8.

26. Dekker J, Buurman BM, van der Leeden M. Exercise in people with comorbidity or multimorbidity. *Health Psychology*. 2019;38(9):822.

27. Dibben GO, Gardiner L, Young HM, et al. Evidence for exercise-based interventions across 45 different long-term conditions: an overview of systematic reviews. *EClinicalMedicine*. 2024;

28. Blodgett J. The association between sedentary behaviour, moderate-vigorous physical activity and frailty. 2014;

29. Eckerblad J, Theander K, Ekdahl A, et al. Symptom burden in community-dwelling older people with multimorbidity: a cross-sectional study. *BMC geriatrics*. 2015;15:1-9.

30. Ross R, Chaput J-P, Giangregorio LM, et al. Canadian 24-hour movement guidelines for adults aged 18–64 years and adults aged 65 years or older: an integration of physical activity, sedentary behaviour, and sleep. *Applied Physiology, Nutrition, and Metabolism*. 2020;45(10):S57-S102.

31. Davies MJ, Aroda VR, Collins BS, et al. Management of hyperglycemia in type 2 diabetes, 2022. A consensus report by the American Diabetes Association (ADA) and the European Association for the Study of Diabetes (EASD). *Diabetes care*. 2022;45(11):2753-2786.

32. Chastin SF, De Craemer M, De Cocker K, et al. How does light-intensity physical activity associate with adult cardiometabolic health and mortality? Systematic review with meta-analysis of experimental and observational studies. *British journal of sports medicine*. 2019;53(6):370-376.

33. Biswas A, Oh PI, Faulkner GE, et al. Sedentary time and its association with risk for disease incidence, mortality, and hospitalization in adults: a systematic review and meta-analysis. *Annals of internal medicine*. 2015;162(2):123-132.

34. Lee SWH, Ng KY, Chin WK. The impact of sleep amount and sleep quality on glycemic control in type 2 diabetes: a systematic review and meta-analysis. *Sleep medicine reviews*. 2017;31:91-101.

35. Pourmotabbed A, Boozari B, Babaei A, et al. Sleep and frailty risk: a systematic review and meta-analysis. *Sleep and Breathing*. 2020;24:1187-1197.

36. Kehler DS, Hay JL, Stammers AN, et al. A systematic review of the association between sedentary behaviors with frailty. *Experimental gerontology*. 2018;114:1-12.

37. Organization WH. *Global action plan on physical activity 2018-2030: more active people for a healthier world*. World Health Organization; 2019.

38. Young HM, March DS, Highton PJ, et al. Exercise for people living with frailty and receiving haemodialysis: a mixed-methods randomised controlled feasibility study. *BMJ open*. 2020;10(11):e041227.

39. Dogra S, Copeland JL, Altenburg TM, Heyland DK, Owen N, Dunstan DW. Start with reducing sedentary behavior: a stepwise approach to physical activity counseling in clinical practice. *Patient education and counseling*. 2022;105(6):1353-1361.

40. Carmona C, Crutwell J, Burnham M, Polak L. Shared decision-making: summary of NICE guidance. *bmj*. 2021;373

41. Foley N, Powell A, Francis-Devine B. House of Commons Library: Informal carers. London, UK: House of Commons Library. Available at: https …; 2021.

42. Muldrew DH, Fee A, Coates V. Impact of the COVID‐19 pandemic on family carers in the community: a scoping review. *Health & Social Care in the Community*. 2022;30(4):1275-1285.

43. Ploeg J, Garnett A, Fraser KD, et al. The complexity of caregiving for community-living older adults with multiple chronic conditions: A qualitative study. *Journal of Comorbidity*. 2020;10:2235042X20981190.

44. Price ML, Surr CA, Gough B, Ashley L. Experiences and support needs of informal caregivers of people with multimorbidity: a scoping literature review. *Psychology & health*. 2020;35(1):36-69.

45. Amer Nordin A, Mohd Hairi F, Choo WY, Hairi NN. Care recipient multimorbidity and health impacts on informal caregivers: a systematic review. *The Gerontologist*. 2019;59(5):e611-e628.

46. Spiers GF, Liddle J, Kunonga TP, et al. What are the consequences of caring for older people and what interventions are effective for supporting unpaid carers? A rapid review of systematic reviews. *BMJ open*. 2021;11(9):e046187.

47. Hippisley-Cox J, Coupland C, Pringle M, Crown N, Hammersley V. Married couples' risk of same disease: cross sectional study. *Bmj*. 2002;325(7365):636.

48. Singh K, Patel SA, Biswas S, et al. Multimorbidity in South Asian adults: prevalence, risk factors and mortality. *Journal of Public Health*. 2019;41(1):80-89.

49. Greaney ML, Kunicki ZJ, Drohan MM, Ward-Ritacco CL, Riebe D, Cohen SA. Self-reported changes in physical activity, sedentary behavior, and screen time among informal caregivers during the COVID-19 pandemic. *BMC Public Health*. 2021;21:1-9.

50. Stowell M, Spiers GF, Kunonga P, et al. Caring for Older People As a Social Determinant of Health: Findings from a Scoping Review of Observational Studies. *Journal of Long-Term Care*. 2024;

51. Kang S, Kim M, Won CW. Spousal concordance of physical frailty in older Korean couples. *International Journal of Environmental Research and Public Health*. 2020;17(12):4574.

52. Lu W-H, Chiou S-T, Chen L-K, Hsiao F-Y. Functional and mental health outcomes of the joint effects of spousal health: the potential threats of “concordant frailty”. *Journal of the American Medical Directors Association*. 2016;17(4):324-330.

53. Jackson SE, Steptoe A, Wardle J. The influence of partner’s behavior on health behavior change: the English Longitudinal Study of Ageing. *JAMA internal medicine*. 2015;175(3):385-392.

54. Polenick CA, Birditt KS, Turkelson A, Kales HC. Individual-level and couple-level discordant chronic conditions: Longitudinal links to functional disability. *Annals of Behavioral Medicine*. 2020;54(7):455-469.

55. Harada K, Masumoto K, Kondo N. Spousal concordance for objectively measured sedentary behavior and physical activity among middle-aged and older couples. *Research quarterly for exercise and sport*. 2018;89(4):440-449.

56. Pauly T, Keller J, Knoll N, et al. Moving in sync: hourly physical activity and sedentary behavior are synchronized in couples. *Annals of Behavioral Medicine*. 2020;54(1):10-21.

57. Horne J, Donald L, Gracia R, et al. Supporting adult unpaid carers via an online dancing intervention: A feasibility/acceptability study. *PLOS global public health*. 2024;4(1):e0002798.

58. Loi SM, Dow B, Ames D, et al. Physical activity in caregivers: What are the psychological benefits? *Archives of Gerontology and Geriatrics*. 2014;59(2):204-210.

59. Lambert SD, Duncan LR, Kapellas S, et al. A descriptive systematic review of physical activity interventions for caregivers: Effects on caregivers’ and care recipients’ psychosocial outcomes, physical activity levels, and physical health. *Annals of Behavioral Medicine*. 2016;50(6):907-919.

60. Doyle KL, Toepfer M, Bradfield AF, et al. Systematic review of exercise for caregiver–care recipient dyads: what is best for spousal caregivers—exercising together or not at all? *The Gerontologist*. 2021;61(6):e283-e301.

61. Epps F, To H, Liu TT, Karanjit A, Warren G. Effect of exercise training on the mental and physical well-being of caregivers for persons living with chronic illnesses: a systematic review and meta-analysis. *Journal of Applied Gerontology*. 2021;40(1):18-27.

62. Kyaw TL, Ng N, Theocharaki M, Wennberg P, Sahlen K-G. Cost-effectiveness of digital tools for behavior change interventions among people with chronic diseases: systematic review. *Interactive Journal of Medical Research*. 2023;12(1):e42396.

63. Taylor ML, Thomas EE, Vitangcol K, et al. Digital health experiences reported in chronic disease management: an umbrella review of qualitative studies. *Journal of telemedicine and telecare*. 2022;28(10):705-717.

64. Kreuter MW, Holt CL. How do people process health information? Applications in an age of individualized communication. *Current Directions in Psychological Science*. 2001;10(6):206-209.

65. Alley S, Jennings C, Persaud N, Plotnikoff RC, Horsley M, Vandelanotte C. Do personally tailored videos in a web-based physical activity intervention lead to higher attention and recall?–an eye-tracking study. *Frontiers in public health*. 2014;2:13.

66. Hamberger M, Ikonomi N, Schwab JD, et al. Interaction empowerment in mobile health: concepts, challenges, and perspectives. *JMIR mHealth and uHealth*. 2022;10(4):e32696.

67. Morgan TL, Faught E, Ross-White A, et al. Tools to guide clinical discussions on physical activity, sedentary behaviour, and/or sleep for health promotion between primary care providers and adults accessing care: a scoping review. *BMC Primary Care*. 2023;24(1):140.

68. Tighe SA, Ball K, Kensing F, Kayser L, Rawstorn JC, Maddison R. Toward a digital platform for the self-management of noncommunicable disease: systematic review of platform-like interventions. *Journal of medical Internet research*. 2020;22(10):e16774.

69. Doyle J, Murphy E, Gavin S, et al. A digital platform to support self-management of multiple chronic conditions (ProACT): findings in relation to engagement during a one-year proof-of-concept trial. *Journal of medical Internet research*. 2021;23(12):e22672.

70. O’connor S, Hanlon P, O’donnell CA, Garcia S, Glanville J, Mair FS. Understanding factors affecting patient and public engagement and recruitment to digital health interventions: a systematic review of qualitative studies. *BMC medical informatics and decision making*. 2016;16:1-15.

71. Taul-Madsen L, Kjeldsen T, Skou ST, Mechlenburg I, Dalgas U. Exercise booster sessions as a mean to maintain the effect of an exercise-intervention-a systematic review. *Physical Therapy Reviews*. 2022;27(2):103-113.

72. de Souto Barreto P. Exercise for multimorbid patients in primary care: one prescription for all? *Sports Medicine*. 2017;47:2143-2153.

73. Beauchamp MK, Lee A, Ward RF, et al. Do exercise interventions improve participation in life roles in older adults? A systematic review and meta-analysis. *Physical therapy*. 2017;97(10):964-974.

74. Parker S, Corner L, Laing K, et al. Priorities for research in multiple conditions in later life (multi-morbidity): findings from a James Lind alliance priority setting partnership. *Age and Ageing*. 2019;48(3):401-406.

75. Groves D, Karsanji U, Evans RA, et al. Predicting future health risk in COPD: differential impact of disease-specific and multi-morbidity-based risk stratification. *International Journal of Chronic Obstructive Pulmonary Disease*. 2021:1741-1754.

76. Rockwood K, Song X, MacKnight C, et al. A global clinical measure of fitness and frailty in elderly people. *Cmaj*. 2005;173(5):489-495.

77. Liguori G, Medicine ACoS. *ACSM's guidelines for exercise testing and prescription*. Lippincott Williams & Wilkins; 2020.

78. Folstein MF, Folstein SE, McHugh PR. “Mini-mental state”: a practical method for grading the cognitive state of patients for the clinician. *Journal of psychiatric research*. 1975;12(3):189-198.

79. NIHR. Payment guidance for researchers and professionals. *National Institute for Health Research, Centre for Engagement and Dissemination Accessed 30th April*. 2021;

80. Wolbring G, Lillywhite A. Equity/equality, diversity, and inclusion (EDI) in universities: the case of disabled people. *Societies*. 2021;11(2):49.

81. O'Neill J, Tabish H, Welch V, et al. Applying an equity lens to interventions: using PROGRESS ensures consideration of socially stratifying factors to illuminate inequities in health. *Journal of clinical epidemiology*. 2014;67(1):56-64.

82. Goodwin VA, Low MS, Quinn TJ, et al. Including older people in health and social care research: best practice recommendations based on the INCLUDE framework. *Age and Ageing*. 2023;52(6):afad082.

83. Nelson LA, Pennings JS, Sommer EC, Popescu F, Barkin SL. A 3-item measure of digital health care literacy: development and validation study. *JMIR Formative Research*. 2022;6(4):e36043.

84. Vandelanotte C, Duncan MJ, Plotnikoff RC, Mummery WK. Do participants' preferences for mode of delivery (text, video, or both) influence the effectiveness of a Web-based physical activity intervention? *Journal of Medical Internet Research*. 2012;14(1):e1998.

85. Frenn M, Malin S, Brown RL, et al. Changing the tide: an Internet/video exercise and low-fat diet intervention with middle-school students. *Applied nursing research*. 2005;18(1):13-21.

86. Soetens KC, Vandelanotte C, de Vries H, Mummery KW. Using online computer tailoring to promote physical activity: a randomized trial of text, video, and combined intervention delivery modes. *Journal of Health Communication*. 2014;19(12):1377-1392.

87. Lee JA. Effect of Web‐based interactive tailored health videos on users' attention, interactivity, overall evaluation, preference and engagement. *Proceedings of the American Society for Information Science and Technology*. 2011;48(1):1-3.

88. Agency for Healthcare Research and Quality. The SHARE Approach—Essential Steps of Shared Decision-making: Quick Reference Guide. 2020;

89. Moore GF, Audrey S, Barker M, et al. Process evaluation of complex interventions: Medical Research Council guidance. *bmj*. 2015;350

90. Martin W, Palazzo C, Poiraudeau S. Development and preliminary psychometrics of the exercise therapy burden questionnaire for patients with chronic conditions. *Archives of Physical Medicine and Rehabilitation*. 2017;98(11):2188-2195. e6.

91. Diabetes Care. 6. Glycemic targets: standards of medical care in diabetes—2019. *Diabetes Care*. 2019;42(Supplement 1):S61-70.

92. Innes E. Handgrip strength testing: A review of the literature. *Australian Occupational Therapy Journal*. 1999;46(3):120-140.

93. Puthoff ML. Research CornerOutcome Measures in Cardiopulmonary Physical Therapy: Short Physical Performance Battery. *Cardiopulmonary physical therapy journal*. 2008;19(1):17-22.

94. Blum L, Korner-Bitensky N. Usefulness of the Berg Balance Scale in stroke rehabilitation: a systematic review. *Physical therapy*. 2008;88(5):559-566.

95. Kudlac M, Sabol J, Kaiser K, Kane C, Phillips RS. Reliability and validity of the berg balance scale in the stroke population: a systematic review. *Physical & Occupational Therapy in Geriatrics*. 2019;37(3):196-221.

96. Burchartz A, Anedda B, Auerswald T, et al. Assessing physical behavior through accelerometry–state of the science, best practices and future directions. *Psychology of Sport and Exercise*. 2020;49:101703.

97. Edwardson CL, Maylor BD, Dawkins NP, Plekhanova T, Rowlands AV. Comparability of postural and physical activity metrics from different accelerometer brands worn on the thigh: Data harmonization possibilities. *Measurement in Physical Education and Exercise Science*. 2022;26(1):39-50.

98. Devlin NJ, Shah KK, Feng Y, Mulhern B, Van Hout B. Valuing health‐related quality of life: An EQ‐5 D‐5 L value set for E ngland. *Health economics*. 2018;27(1):7-22.

99. Brazier JE, Harper R, Jones N, et al. Validating the SF-36 health survey questionnaire: new outcome measure for primary care. *British medical journal*. 1992;305(6846):160-164.

100. Jette AM, Haley SM, Coster WJ, et al. Late life function and disability instrument: I. Development and evaluation of the disability component. *The Journals of Gerontology Series A: Biological Sciences and Medical Sciences*. 2002;57(4):M209-M216.

101. Jette AM, Haley SM, Kooyoomjian JT. Late-life FDI manual. *Boston, MA: Boston University*. 2002;73

102. Rugno FC, Carlo MMRdPD. The Palliative Outcome Scale (POS) applied to clinical practice and research: an integrative review. *Revista latino-americana de enfermagem*. 2016;24:e2764.

103. Schlosser RW. Goal attainment scaling as a clinical measurement technique in communication disorders: a critical review. *Journal of communication disorders*. 2004;37(3):217-239.

104. Yap P. Validity and reliability of the Zarit Burden Interview in assessing caregiving burden. *Ann Acad Med Singapore*. 2010;39(10):758-763.

105. Skivington K, Matthews L, Simpson SA, et al. A new framework for developing and evaluating complex interventions: update of Medical Research Council guidance. *International Journal of Nursing Studies*. 2024;

106. May CR, Mair F, Finch T, et al. Development of a theory of implementation and integration: Normalization Process Theory. *Implementation Science*. 2009;4:1-9.

107. Murray E, Treweek S, Pope C, et al. Normalisation process theory: a framework for developing, evaluating and implementing complex interventions. *BMC medicine*. 2010;8:1-11.

108. Walton H, Spector A, Williamson M, Tombor I, Michie S. Developing quality fidelity and engagement measures for complex health interventions. *British journal of health psychology*. 2020;25(1):39-60.

109. Doherr H, Christalle E, Kriston L, Härter M, Scholl I. Use of the 9-item Shared Decision Making Questionnaire (SDM-Q-9 and SDM-Q-Doc) in intervention studies—a systematic review. *PLoS one*. 2017;12(3):e0173904.

110. Malterud K, Siersma VD, Guassora AD. Sample size in qualitative interview studies: guided by information power. *Qualitative health research*. 2016;26(13):1753-1760.

111. Ho IS, Azcoaga-Lorenzo A, Akbari A, et al. Measuring multimorbidity in research: Delphi consensus study. *BMJ medicine*. 2022;1(1)

112. Ritchie J, Lewis J, Nicholls CM, Ormston R. *Qualitative research practice*. vol 757. sage London; 2003.

113. Robinson OC. Sampling in interview-based qualitative research: A theoretical and practical guide. *Qualitative research in psychology*. 2014;11(1):25-41.

114. Aiden H. Multimorbidity. *Understanding the Challenge*. 2018;

115. Creswell JW, Clark VLP. *Designing and conducting mixed methods research*. Sage publications; 2017.

116. Arain M, Campbell MJ, Cooper CL, Lancaster GA. What is a pilot or feasibility study? A review of current practice and editorial policy. *BMC medical research methodology*. 2010;10:1-7.

117. Tickle-Degnen L. Nuts and bolts of conducting feasibility studies. *The American Journal of Occupational Therapy*. 2013;67(2):171-176.

118. Arnold DM, Burns KE, Adhikari NK, Kho ME, Meade MO, Cook DJ. The design and interpretation of pilot trials in clinical research in critical care. *Critical care medicine*. 2009;37(1):S69-S74.

119. Sim J, Lewis M. The size of a pilot study for a clinical trial should be calculated in relation to considerations of precision and efficiency. *Journal of clinical epidemiology*. 2012;65(3):301-308.

120. Julious SA. Sample size of 12 per group rule of thumb for a pilot study. *Pharmaceutical Statistics: The Journal of Applied Statistics in the Pharmaceutical Industry*. 2005;4(4):287-291.

121. Thabane L, Ma J, Chu R, et al. A tutorial on pilot studies: the what, why and how. *BMC medical research methodology*. 2010;10:1-10.

122. Lancaster GA, Dodd S, Williamson PR. Design and analysis of pilot studies: recommendations for good practice. *Journal of evaluation in clinical practice*. 2004;10(2):307-312.

123. Eldridge SM, Chan CL, Campbell MJ, et al. CONSORT 2010 statement: Extension to randomised pilot and feasibility trials. BMJ, 355, i5239. 2016.

124. Eldridge SM, Lancaster GA, Campbell MJ, et al. Defining feasibility and pilot studies in preparation for randomised controlled trials: development of a conceptual framework. *PloS one*. 2016;11(3):e0150205.

125. Gamble C, Krishan A, Stocken D, et al. Guidelines for the content of statistical analysis plans in clinical trials. *Jama*. 2017;318(23):2337-2343.

126. Eldridge SM, Chan CL, Campbell MJ, et al. CONSORT 2010 statement: extension to randomised pilot and feasibility trials. *bmj*. 2016;355

127. Avery KN, Williamson PR, Gamble C, et al. Informing efficient randomised controlled trials: exploration of challenges in developing progression criteria for internal pilot studies. *BMJ open*. 2017;7(2):e013537.

128. Zhu Y, Edwards D, Mant J, Payne RA, Kiddle S. Characteristics, service use and mortality of clusters of multimorbid patients in England: a population-based study. *BMC medicine*. 2020;18:1-11.

129. Soley-Bori M, Bisquera A, Ashworth M, et al. Identifying multimorbidity clusters with the highest primary care use: 15 years of evidence from a multi-ethnic metropolitan population. *British Journal of General Practice*. 2022;72(716):e190-e198.

130. Terry G, Hayfield N, Clarke V, Braun V. Thematic analysis. *The SAGE handbook of qualitative research in psychology*. 2017;2(17-37):25.

131. Tashakkori A, Teddlie C. *Sage handbook of mixed methods in social & behavioral research*. SAGE publications; 2021.

132. Fetters MD, Curry LA, Creswell JW. Achieving integration in mixed methods designs—principles and practices. *Health services research*. 2013;48(6pt2):2134-2156.

133. Richards DA, Bazeley P, Borglin G, et al. Integrating quantitative and qualitative data and findings when undertaking randomised controlled trials. *BMJ open*. 2019;9(11):e032081.

134. O’Cathain A, Murphy E, Nicholl J. Three techniques for integrating data in mixed methods studies. *Bmj*. 2010;341

135. Bugge C, Williams B, Hagen S, et al. A process for Decision-making after Pilot and feasibility Trials (ADePT): development following a feasibility study of a complex intervention for pelvic organ prolapse. *Trials*. 2013;14:1-13.

136. Staley K, Elliott J, Stewart D, Wilson R. Who should I involve in my research and why? Patients, carers or the public? *Research Involvement and Engagement*. 2021;7(1):41.

137. McCoy MS, Jongsma KR, Friesen P, et al. National Standards for Public Involvement in Research: missing the forest for the trees. *Journal of Medical Ethics*. 2018;44(12):801-804.

138. Leask CF, Sandlund M, Skelton DA, et al. Framework, principles and recommendations for utilising participatory methodologies in the co-creation and evaluation of public health interventions. *Research involvement and engagement*. 2019;5:1-16.

139. NIHR. Guidance on co-producing a research project. INVOLVE. <https://www>. invo. org. uk/wpcontent/uploads/2019/04 …; 2018.

140. Garratt A, Sagen J, Børøsund E, et al. The public and patient engagement evaluation tool: forward-backwards translation and cultural adaption to Norwegian. *BMC musculoskeletal disorders*. 2022;23(1):556.

# Appendices

**Appendix A: The Clinical Frailty Scale**


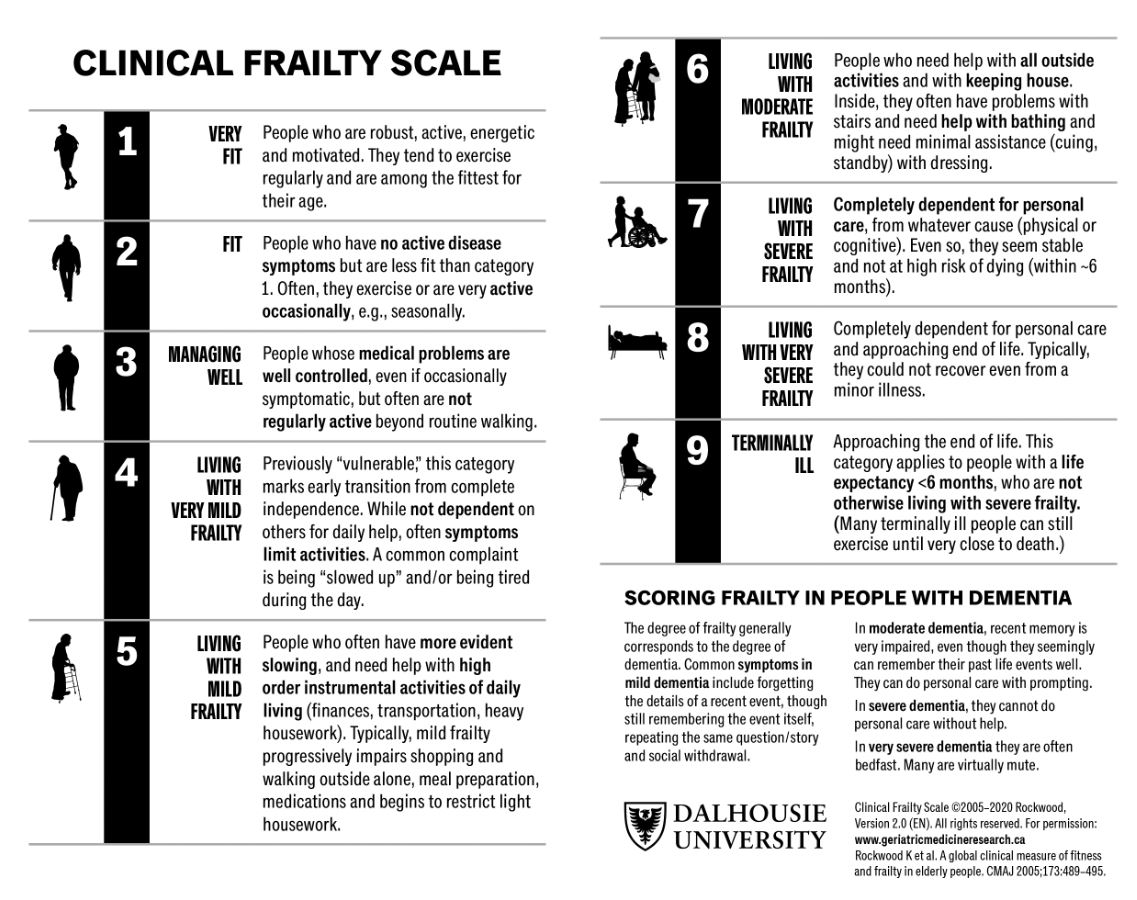


**Appendix B: Data collection form for physical activity officer and community provider linkage processes**

| 1. **Participant details** | |
| --- | --- |
| Participant study ID |  |
| Date of appointment |  |
| 1. **Attendance at Group / Activity sessions** | |
| Name of Group(s) / Activity(s) referred to |  |
| Did the participant attend the first session?  (Note: Only need to complete at first review) | - Yes - No   If no, please indicate reason(s): |
| How many sessions did the participant **attend in total** |  |
| Did the carer attend any sessions? | - Yes - No   If yes, how many sessions? |
| Did PA Officer attend any sessions with the participant? | - Yes - No   If yes, how many sessions? |
| Were any adjustments required to enable the participant to attend and participate?  (e.g. psychological, social or physical needs) | - Yes - No   If yes, please provide adjustment(s):  Was the group / activity able to make this adjustment?   - Yes - No |
| 1. **Community Group / Activity Information** | |
| Group / Activity name |  |
| Group / Activity leader contact details | Name:  Email address:  OR  Contact number: |
| Primary location of Group / Activity |  |
| Environment description  (e.g. an outdoor park, cafe, gym, library, community centre etc.) |  |
| Aim or purpose of Group / Activity |  |
| Description of content  (e.g. walking, yoga, strength training, discussing, crafting) |  |
| Target population of Group / Activity  (e.g. older adults, women, beginners, religious group, families etc.) |  |
| Duration of sessions |  |
| Frequency of sessions  (e.g. 2x/week, weekly, bi-weekly, monthly etc.) |  |
| How did the participant(s) travel to group or activity  (How safe is the route to the group or activity? E.g. is it a well populated area and well-lit?) |  |
| Cost of sessions  (e.g. cost to attend, cost of travel and parking) |  |

**Appendix C. Draft local authority profile questionnaire**

| Local and community context | |
| --- | --- |
| Area Name |  |
| Number of patients living with MLTCs and frailty in area |  |
| Deprivation score of area |  |
| Any known barriers of linkage or uptake to groups/services due to deprivation in area  (e.g. low transport access, digital exclusion, lack of groups/services) |  |
| Languages commonly spoken in community (other than English) |  |
| Ethnicities |  |
| Services/groups/activities characteristics | |
| Approximate number of known services/groups/activities in area related to 24-hour movement behaviours |  |
| Approximate number of available services/groups/activities targeted towards populations | Older adults:  Younger adults:  Beginners:  Intermediate/advanced:  Non-English speaking:  Religious groups:  Women only:  Families:  Low mobility:  Long-term condition specific:  Carers only:  Other (please specify): |
| Number of cost-free or subsidised services and community groups related to 24-hour movement behaviours (for users) |  |
| Number of services/groups/activities which offer financial incentives (for Active Together/GP practice referrals) |  |
| Number of services/groups/activities where sessions are offered outside of usual working hours (9am-5pm) |  |
| Number of services/groups/activities where sessions are offered ONLY DURING usual working hours (9am-5pm) |  |
| Other comments availability/accessibility of services/groups/activities in this geographic area that influence linkage and uptake |  |

**Appendix D: Topic Guide: People living with Multiple Long-Term Conditions.**

**Guide for interviewer**

- Introduce yourself and your role
- Briefly explain the purpose of the interview
- Remind participant that the interviews will be anonymised and kept confidential
- Ask permission to audio record the interview
- Ask if participant has any questions before we begin the interview
- Begin recording

**Motivations to take part and being approached for study**

- Where did you hear about the trial?
- How were you approached to take part in the study? (How did you find this? Is there any way this could be improved?)
- Can you tell me about why you were interested in participating in the study? (What part(s) of the trial were you most interested in and why? Have you been involved in other research?)

**Information sheet, consent decision making and approach to consent**

- Can you tell us about your experiences of being recruited into the trial?
  - Information sheet
  - Leaflets
  - Communication
  - Consent
  - Suggestions for improvements/ refinements to all of the above

**Outcome measures**

- How did you find completing the outcome measures as part of the trial?
  - Questionnaires
  - Functional measures
  - Body composition
  - Challenges/ difficulties for any of these
  - Burden of these – content, volume of measures, number of timepoints
- How do you understand the purpose of these outcome measures?
- Which of the outcomes felt most important or meaningful to you?
- Were there any outcomes which didn’t feel relevant or important to you?
- If we ran this trial again, can you think of anything else that we should be measuring as part of the trial?

**Acceptability of intervention**

- What was your experience of PERSONAL-AGILITY overall?
- In your own words, can you describe the purpose of the intervention? (Did all parts of the intervention make sense to you?)
- What did you think about the format of the PERSONAL-AGILITY intervention being a mixture of online and in-person appointments?
- What were your experience of using the different resources for the trial?:
  - MyHealthMap
  - Steps4Health
  - personalised videos
  - Personal decision aid (was the information balanced? Did it help you to decide or did you already know what behaviour to focus on?)
  - Guide for two [if applicable]
- Do you think the intervention helped you make shared decisions with your healthcare professional?
- Is there any way your healthcare professional could have improved how they delivered the intervention?
- [Optional, for dyads] How did you find the involvement of your carers/family members in the intervention?
  - How did you work together through the intervention, if at all?
  - Did this impact your relationship with them in any way?
  - Do you think the intervention had any benefits for you both?
- [Optional for single participants] You had the option in this trial to involve a care or a significant other, why did you decide not to do this?
- How did you find your appointments with a physical activity officer/ as part of the trial?
- Did they refer you to any groups or other services?
- What was your experience of attending the community services OR why did you choose not to attend any community services
  - Challenges/ barriers
  - Facilitators
- Were there any parts of PERSONAL-AGILITY that worked well for you?
- Were there any parts of the intervention that you think could be improved?

**Impact of intervention**

- How much impact did the study have on you?
  - Physical activity
  - Sitting time
  - Sleep
  - Wellbeing
  - Independence/ everyday tasks
  - For all of the above, prompt for examples.
- Were there any other ways your life has been impacted by the intervention?
- Did anything negative happen as a result of the intervention?
  - Burden of the intervention

**Barriers and facilitators to engagement**

- Did you experience any barriers to engaging in the intervention? (Examples)
- Did anything facilitate or help you engage in the intervention? (Examples)
- Did anything happen outside of the trial which affected your ability to engage in the intervention?
- Is there anything else you would like to tell us about the intervention?

**Appendix E: Topic Guide: Carers and relatives**

**Guide for interviewer**

- Introduce yourself and your role
- Briefly explain the purpose of the interview
- Remind participant that the interviews will be anonymised and kept confidential
- Ask permission to audio record the interview
- Ask if participant has any questions before we begin the interview
- Begin recording

**Motivations to take part and being approached for study**

- How were you approached to take part in the study? (Did your relative that you care for mention it to you? Would you have preferred to find out about it a different way?)
- Can you tell me about why you were interested in participating in the study? (What part(s) of the trial were you most interested in and why? Have you been involved in other research?)

**Information sheet, consent decision making and approach to consent**

- Can you tell us about your experiences of being recruited into the trial?
  - Information sheet
  - Leaflets
  - Communication
  - Consent
  - Suggestions for improvements/ refinements to all of the above

**Outcome measures**

- How did you find completing the outcome measures as part of the trial?
  - Questionnaires
  - Functional measures
  - Body composition
  - Challenges/ difficulties for any of these
  - Burden of these – content, volume of measures, number of timepoints
- How do you understand the purpose of these outcome measures?
- Which of the outcomes felt most important or meaningful to you?
- Were there any outcomes which didn’t feel relevant or important to you?
- If we ran this trial again, can you think of anything else that we should be measuring as part of the trial?

**Acceptability of intervention**

- What was your experience of PERSONAL-AGILITY overall?
- In your own words, can you describe the purpose of the intervention? (Did all parts of the intervention make sense to you?)
- What did you think about the format of the PERSONAL-AGILITY intervention being a mixture of online and in-person appointments?
- What were your experience of using the different resources for the trial?:
  - MyHealthMap
  - Steps4Health
  - personalised videos
  - Personal decision aid (was the information balanced? Did it help you to decide or did you already know what behaviour to focus on?)
  - Guide for two
- Do you think the intervention helped you make shared decisions with your healthcare professional?
- Is there any way your healthcare professional could have improved how they delivered the intervention?
- [Optional, for dyads] How did you find the involvement of your carers/family members in the intervention?
  - How did you work together through the intervention, if at all?
  - Did this impact your relationship with them in any way?
  - Do you think the intervention had any benefits for you both?
- How did you find your appointments with a physical activity officer/ as part of the trial?
- Did they refer you to any groups or other services?
- What was your experience of attending the community services OR why did you choose not to attend any community services
  - Challenges/ barriers
  - Facilitators
- Were there any parts of PERSONAL-AGILITY that worked well for you?
- Were there any parts of the intervention that you think could be improved?

**Impact of intervention**

- How much impact did the study have on you?
  - Physical activity
  - Sitting time
  - Sleep
  - Wellbeing
  - Independence/ everyday tasks
  - Caring responsibilities/ role
  - For all of the above, prompt for examples.
- Were there any other ways your life has been impacted by the intervention?
- Did anything negative happen as a result of the intervention?
  - Burden of the intervention
  - Caring responsibilities/ role

**Barriers and facilitators to engagement**

- Did you experience any barriers to engaging in the intervention? (Examples)
- Did anything facilitate or help you engage in the intervention? (Examples)
- Did anything happen outside of the trial which affected your ability to engage in the intervention?
- Is there anything else you would like to tell us about the intervention

**Appendix F: Topic Guide for Physical activity officers**

**Guide for interviewer**

- Introduce yourself and your role
- Briefly explain the purpose of the interview
- Remind participant that the interviews will be anonymised and kept confidential
- Ask permission to audio record the interview
- Ask if participant has any questions before we begin the interview
- Begin recording

**Introduction**

- Can you tell me about your role?
- Could you tell me about why you were interested in being involved in this trial? (What information were you provided about the trial? Were there any elements in particular you were interested in? Did you have any concerns before the trial began?

**Perceptions of PERSONAL-AGILITY**

- How did you find delivering the PERSONAL-AGILITY intervention? (How did you deliver the intervention with patients and their carers? Was this together or separately?)
- Did you experience any participant resistance to the intervention?
- What were your experiences and feelings about working with participants and carers as a dyad?
- How did you find working with the trial team?
  - Communication
  - Handovers
- online systems for the intervention and the study (My health Mapp, Steps for Health)Tell us about your caseload and how you managed this
  - Complexity
  - number
- What were your experiences of linking participants in the study to community groups in their area
  - Challenges/ barriers (individual and community level)
  - Facilitators (individual and community level)
- What were your experiences of accessing/ referring people into the community groups/ working with community providers?
  - Challenges/ barriers
  - Facilitators
- Did you observe any benefits of the intervention? If so what are these?
  - For participants
  - for carers
  - for yourself/ your role
- Where there any negative impacts you observed as a result of the intervention?
  - Burden (participants, carers, themselves)
- Can you think of any way the intervention needs to be adapted or modified? (From a provider point of view? From a patient point of view?)

**Contextual barriers and facilitators**

- Did anything facilitate or help you to implement the intervention? (Any further training or courses, advice from colleagues, personal experience?)
- Did anything happen outside of the trial which affected your ability to implement the intervention? (Has there been any system level or practical factors which influenced how it was implemented?)
- If we were to run this trial more widely, in a greater number of areas/ regions what changes might you suggest?

**Appendix G: Topic Guide: Community Providers.**

**Guide for interviewer**

- Introduce yourself and your role
- Briefly explain the purpose of the interview
- Remind participant that the interviews will be anonymised and kept confidential
- Ask permission to audio record the interview
- Ask if participant has any questions before we begin the interview
- Begin recording

**Introduction**

- Can you tell me about your role?
  - Background
  - Training
  - Knowledge of long-term conditions and/ or MLTCs
  - Knowledge of frailty
- Can you tell me about the group/service that you run?
  - Aims
  - Target population
  - Did they set it up, how did they become involved

**Physical activity officer interaction**

- Tell us about your experiences of interacting with the physical activity officer
  - Communication
  - Referral
  - Suggestions for improvement

**Experiences of supporting the participant**

- Tell us about your experience of supporting the participant
  - Accessibility
  - Needs
  - Did you have to make any adjustments so they could participate?
  - Atttitudes to making adjustments
  - Challenges (including challenges to making adjustments)
  - Suggestions for improvements
  - Thoughts on carer involvement (if applicable)
- What were your perceptions of how they got on in the group/service
  - Challenges
  - Facilitators
  - Level of enjoyment
  - Level of interaction with others in the group/ service
- What were your impressions of the impact of coming to the group/ service for the participant?
- Where there any drawbacks or negative impacts?
- Where there any benefits or drawbacks for you as a provider?
- If we were to implement this linkage approach more widely, do you have any suggestions or advice on how we could do this?
  - Challenges forseen
  - What might help/ overcome these?
