# Supplementary figures and images for: Maintaining function and participation through tailored 24-hour movement behaviours for people living with multiple long-term conditions and frailty (The PERSONAL-AGILITY study): Protocol for a randomised controlled feasibility trial
Source: PLoS One. 2026 May 18;21(5):e0348372. doi: 10.1371/journal.pone.0348372 (PMC13183243; doi:10.1371/journal.pone.0348372)

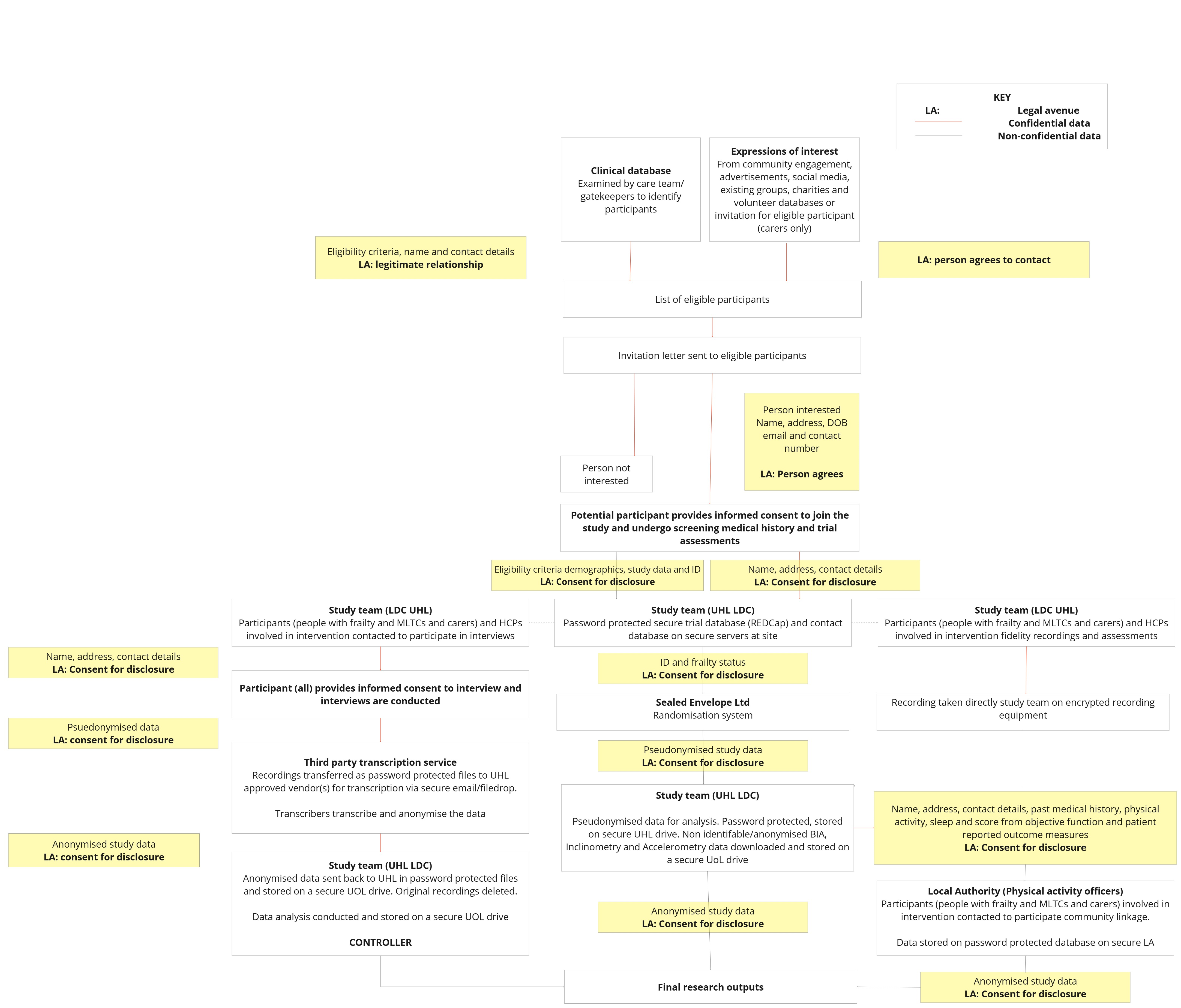

Supplement: S1 Fig — Abbreviations: LA, Legal Avenue; LDC, Leicester Diabetes Centre; UHL, University Hospitals of Leicester; MLTC, Multiple long-term conditions; HCP, Health care professional; UOL, University of Leicester. (TIF) [file pone.0348372.s003.tif]
